# Machine Learning-based Framework Develops a Tumor Thrombus Coagulation Signature in Multicenter Cohorts for Renal Cancer

**DOI:** 10.7150/ijbs.94555

**Published:** 2024-07-01

**Authors:** Tao Feng, Yue Wang, Wei Zhang, Tingting Cai, Xi Tian, Jiaqi Su, Zihao Zhang, Shengfeng Zheng, Shiqi Ye, Bo Dai, Ziliang Wang, Yiping Zhu, Hailiang Zhang, Kun Chang, Dingwei Ye

**Affiliations:** 1 Qingdao Institute, School of Life Medicine, Department of Urology, Fudan University Shanghai Cancer Center, Fudan University, Qingdao, 266500, China.; 2 Department of Urology, State Key Laboratory of Genetic Engineering, Collaborative Innovation Center for Genetics and Development, School of Life Sciences, Fudan University Shanghai Cancer Center, Fudan University, Shanghai, 200433, China.; 3 Department of Urology, Fudan University Shanghai Cancer Center, No. 270 Dong'an Road, Shanghai, 200032, People's Republic of China.; 4 Central Laboratory, Shanghai Municipal Hospital of Traditional Chinese Medicine, Shanghai University of Traditional Chinese Medicine, 274 Middle Zhijiang Road, Shanghai 200071, China.

**Keywords:** Machine learning, Tumor thrombus, renal cell carcinoma, immunotherapy, CYP51A1

## Abstract

**Background:** Renal cell carcinoma (RCC) is frequently accompanied by tumor thrombus in the venous system with an extremely dismal prognosis. The current Tumor Node Metastasis (TNM) stage and Mayo clinical classification do not appropriately identify preference-sensitive treatment. Therefore, there is an urgent need to develop a better ideal model for precision medicine.

**Methods:** In this study, we developed a coagulation tumor thrombus signature for RCC with 10 machine-learning algorithms (101 combinations) based on a novel computational framework using multiple independent cohorts.

**Results:** The established tumor thrombus coagulation-related risk stratification (TTCRRS) signature comprises 10 prognostic coagulation-related genes (CRGs). This signature could predict survival outcomes in public and in-house protein cohorts and showed high performance compared to 129 published signatures. Additionally, the TTCRRS signature was significantly related to some immune landscapes, immunotherapy response, and chemotherapy. Furthermore, we also screened out hub genes, transcription factors, and small compounds based on the TTCRRS signature. Meanwhile, CYP51A1 can regulate the proliferation and migration properties of RCC.

**Conclusions:** The TTCRRS signature can complement the traditional anatomic TNM staging system and Mayo clinical stratification and provide clinicians with more therapeutic options.

## Introduction

In 2020, renal cell carcinoma (RCC) was recognized as originating from the epithelial cells of the urinary tubules, affecting approximately 431,288 newly diagnosed patients worldwide and resulting in a staggering death toll of 179,368 1. Among the malignancies, RCC patients exhibit a heightened vulnerability to venous thromboembolic complications 2 (VTE), with a distinctive clinical hallmark being the potential migration of the tumor to the renal vein or inferior vena cava, thereby giving rise to the formation of a venous tumor thrombus (TT) 3,4. According to statistics, TT incidence accounts for approximately 15% of RCC cases 5. The prognosis for RCC patients with TT is extremely poor, with a median survival dropping to just 5 months and a 1-year disease-specific survival (DSS) rate of only 29% in the absence of treatment 6.

RCC-TT presents a complex pathogenesis characterized by significant heterogeneity. Coagulation can be seen as a response to hemorrhage resulting from blood vessel wall disruption and intravascular coagulation or activated with increased blood plasma and vascular permeability leading to extravascular coagulation 7. Additionally, multiple studies have underscored the pivotal role of platelets, VEGF, and their signal transduction pathways in TT formation 8-11. In recent years, the tumor microenvironment (TME) has gained increasing recognition for its involvement in tumor coagulation, as demonstrated by pan-cancer analyses 12. Despite traditional staging systems such as Tumor-Node-Metastasis (TNM) 13 and Mayo clinical classification of TT in RCC 14 dominating clinical decision-making and therapeutic management in clinical practice, their limitations in comprehensively assessing molecular biological characteristics may impede the determination of optimal clinical strategies 15. With the rapid advancements in genomics and bioinformatics, various biomarkers in RCC patients with TT (RCC-TT) have been identified 16,17. However, limited data availability, inappropriate utilization of machine-learning methods, and insufficient validation of large, diverse patient cohorts hinder the clinical applications of these biomarkers 18-20. It would seem, therefore, that further construction of a robust RCC-TT model is urgently needed to be fully implemented into routine clinical practice.

In this study, our aim was to develop and validate a tumor thrombosis coagulation-related risk signature (TTCRRS) tailored specifically for renal cell carcinoma (RCC) patients. By leveraging a comprehensive set of coagulation-related genes (CRGs) and employing a sophisticated ensemble of machine learning algorithms, we meticulously constructed and refined the TTCRRS using data from 16 independent cohorts. Notably, our TTCRRS demonstrated remarkable robustness, exhibiting exceptional predictive performance for crucial clinical outcomes, including survival prognosis, immunotherapy response, and drug efficacy. By furnishing up-to-date and comprehensive information, our novel signatures hold immense potential to inform and empower healthcare professionals, enabling them to optimize precision treatment approaches within the RCC domain. This, in turn, facilitates personalized and targeted interventions, ultimately leading to improved patient outcomes.

## Methods

### Collection and processing of publicly available data

A total of 1573 RCC patients were gathered from 13 datasets from The Cancer Genome Atlas (TCGA), Gene Expression Omnibus (GEO), CM-025, and E-MTAB-1980. Among these datasets, 5 datasets (TCGA, ICGC, GSE167573, CM-025, E-MTAB-1980) with adequate information on survival outcomes were utilized to construct and validate our signature. Furthermore, to comprehensively explore the impact of chemo-immunotherapy, we incorporated an additional five drug-related datasets (IMvigor210, GSE91061, GSE78220, GSE135222, and GSE35640). Of them, The IMvigor210 immunotherapy cohort comprises 348 patients diagnosed with bladder urothelial carcinoma who received treatment with anti-PD-L1 agents. This cohort data was sourced from a published study utilizing the R package “IMvigor210CoreBiologies” 21. In addition, the GSE91061 cohort was retrieved from pre- and post-treatment tumor samples taken from melanoma patients who received anti-PD-1 and anti-CTLA4 therapy 22. The VTE-cohorts dataset (GSE48000) was utilized for the assessment of coagulation-related gene (CRG) expression levels. To gain insights into the role of protein expression levels, we leveraged the Clinical Proteomic Tumor Analysis Consortium (CPTAC) cohort, encompassing 232 tumor samples and 232 normal samples, along with the Proteomic Cohort from Fudan University Shanghai Cancer Center (FUSCC), consisting of 232 tumor samples and 232 paired normal samples. These cohorts served as invaluable resources for investigating the relationship between the TTCRRS and protein expression levels.

TCGA raw read counts were converted to transcripts per kilobase million (TPM) from UCSC Xena (https://xenabrowser.net/) and log2 transformed. For TCGA samples, all data were aligned and annotated on the GENCODE Homo sapiens GRCh38 (hg38) reference genome. The robust multichip average (RMA) algorithm implemented in the R package affy was used to process and normalize the raw data 23. Meanwhile, we used annotation files available in GPL platforms to convert probes into gene symbols. Meta-cohort was combined and normalized (removed the batch effect) the ICGC, GSE167573, CM-025, and E-MTAB-1980 datasets with the function ComBat 24 from the “sva” package 25. Furthermore, the protein expression profiles of RCC were collected from the CPTAC database, and the details of our FUSCC cohort were reported in a previous study 26. Table S1 summarizes the baselines for the 16 enrolled datasets.

Herein, From the KEGG database (https://www.genome.jp/kegg/), complement and coagulation cascades (hsa04610) and platelet activation (hsa04611) were acquired. Identification of genes associated with TT in patients with RCC, we referred to the research conducted by Ma et al 17, which reported genes exhibiting a higher mutation frequency in RCC patients with TT compared to RCC patients without TT. Our study defined genes in coagulation pathways and higher mutation frequency in tumor thrombus RCC patients as tumor thrombosis coagulation related genes (TTCRGs). These genes were designated as TTCRGs in our study, amounting to a total of 1,971 genes. By focusing on genes within the coagulation pathways and exhibiting higher mutation frequencies in tumor thrombus RCC patients, we aimed to capture crucial molecular factors involved in the coagulation process within the context of RCC with tumor thrombus.

### Somatic mutation and copy number alteration analysis

Genetic alterations in TTCRGs can be classified as somatic copy number variations (SCNAs) or somatic mutations. a better understanding of TTCRGs from their genomic characteristics to gain, we examined the frequency of somatic mutations and SCNAs, and their correlation with patient prognosis in different groups. All the above data as well as the entire clinical information were downloaded from cBioPortal (https://www.cbioportal.org) 27. Analysis and visualization of mutation characteristics were performed using the R package “maftools” 28, depicting the mutation frequency of TTCRGs through an OncoPrint plot. In the kidney renal clear cell carcinoma datasets, we selected the genomic profiles of mutation and putative copy-number alterations from GISTIC to investigate the genomic characteristics between the altered and unaltered groups. Within the GDC GISTIC copy number dataset obtained from cBioPortal, we designated the values of 2 and -2 as amplification and deep deletion, respectively. A survival analysis was used to investigate the effect of mutations on patient outcomes in patients with and without DNA alteration (SCNA versus mutations).

### Subtypes identification of TTCRGs

To identify distinct med on TTCRGs expression in RCC patients, we employed three feature-based methods: Consensus clustering (CC) 29, Consensus nonnegative matrix factorization (CNMF) 30, Combined similarity network fusion and CC (SNF-CC) 31 in the R package “CancerSubtypes” 31 were executed by repeating 500 times. Subsequently, the optimal number of clusters was determined using cumulative distribution function curves (CDF), silhouette plots, consensus score matrices and survival analyses. We used R package GSVA's 32 single sample gene set enrichment analysis (ssGSEA) to analyze 28 different immune cells to determine their relative infiltration in each RCC patient 33. In addition, the stability and robustness of the ssGSEA results were further validated to ensure their reliability, we performed an ensemble approach, incorporating 6 alternative algorithms (TIMER 34, quanTIseq 35, MCP-counter 36, xCell 37, EPIC 38 and ESTIMATE 39) with default parameters. To unravel the intricate interplay between the identified coagulation subtypes and diverse clinicopathological features, encompassing grade, stage, and patient status were generated by the Sankey diagram using ggalluvial R package.

### The immune-related features of TTC subtypes based on enrichment analysis

In the R package GSVA, normalized enrichment scores (NES) are calculated for pathways using gene set variation analysis (GSVA) 32, enabling a comprehensive exploration of biological functions within the TTC clusters, and were displayed in NES heatmap for RCC patients. Then, the differentially expressed genes (DEGs) obtained by the limma R package 40 between two TTC subtypes were applied to execute the procedure of the gene set enrichment analysis (GSEA) with aid of the R package clusterProfiler 41 using two MeSH terms (gendoo 42 and gene2pubmed 43) as the reference gene set. To investigate the immune characteristics of the TTCRGs, a boxplot analysis was performed to compare the T cell stimulators and the gene expression of major histocompatibility complexes between different clusters. In addition, the tumor microenvironment (TME) composition within the two TCC clusters was assessed using the MCP-counter tool 36, which provides robust quantification of 8 immune cell types (CD8+ T cells, T cells, natural killer cells, cytotoxic lymphocytes, monocytic lineage, B cell lineage, myeloid dendritic cells, and neutrophils) and two stromal cell populations (fibroblasts and endothelial cells) across diverse cohorts. Tumor tissue was examined for the presence of immune and stromal cells, as well as the level of DNA methylation of tumor-infiltrating lymphocytes (MeTILs) using the R package ESTIMATE 39, in accordance with established protocols outlined in the literature 44. Ultimately, the pRRophetic R package employed a ridge regression method to predict the semi-inhibitory concentration (IC50) values, primarily utilized in chemotherapeutic drugs, for each RCC patient. This analysis aimed to compare the effectiveness of these drugs among different TTC subtypes 45,46.

### Weight correlation network analysis (WGCNA)

To further capture genes with highly relevant associations with the hypercoagulable state of TTCRGs, we applied two VTE cohorts to execute the WGCNA procedure with the R package WGCNA 47. Based on the scale-free network criteria, a soft threshold was selected. Then, Topological overlap matrix (TOM) was generated from adjacency matrix to reflect the topological information of a network ad genes were hierarchically clustered with dissimilarity (1-TOM) of the topological overlap. By using dynamic tree cutting, the key module was identified. After that, a module with the highest correlation to VTE was selected as the module to further study to recognize significant coagulation modules associated with VTE.

### The signature generated from machine learning-based integrative approaches

We integrated 10 machine learning algorithms and 101 algorithm combinations according to Liu's research to develop TTCRRS with high accuracy and stability 48. Our integrative algorithms included generalized boosted regression modeling (GBM), Least absolute shrinkage and selection operator (LASSO), elastic network (Enet), random survival forest (RSF), ridge, stepwise Cox, partial least squares regression for Cox (plsRcox), CoxBoost, survival support vector machine (survival-SVM) and supervised principal components (SuperPC). There were several algorithms that had features selection functionalities, including stepwise Cox, Lasso, RSF and CoxBoost. The RSF model, executed through the random ForestSRC package, optimized parameters (ntree and mtry) via grid-search with LOOCV. Lasso, Enet, and Ridge facilitated by the glmnet package, determined the regularization parameter (λ) through LOOCV and set the L1-L2 trade-off parameter (α) within the interval of 0-1 (step 0.1). The Stepwise Cox model, integrated within the survival package, conducted an iterative stepwise search based on the Akaike Information Criterion (AIC), exploring both forward and backward directions for feature selection. CoxBoost, facilitated by the CoxBoost package, employed componentwise likelihood-based boosting, optimizing the penalty term via LOOCV (optimCoxBoostPenalty function) and the boosting steps using cv.CoxBoost routine. The plsRcox model, operationalized through the plsRcox package, utilized cv.plsRcox to ascertain the optimal number of components and further utilized the plsRcox function for fitting a generalized linear model based on partial least squares regression. SuperPC, harnessed from the superpc package, applied superpc.cv for supervised principal component analysis and feature threshold estimation, with a special consideration for the 'pre-validation' approach when fitting Cox models to smaller validation datasets. The GBM model, accessed via the gbm function within the superpc package, fine-tuned the number of trees by minimizing cross-validation error through cv.gbm. Lastly, the survival-SVM model, leveraging the survivalsvm package, implemented a regression-based approach, accounting for censoring while formulating inequality constraints within the support vector problem.

The formulation of the TTCRRS ensued through a sequential methodology: (a) Identification of prognostically significant TTCRGs within the TCGA-KIRC cohort was accomplished via Univariate Cox regression analysis; (b) Subsequently, leveraging these pivotal TTCRGs, an exhaustive exploration involving 101 algorithmic permutations was conducted to fashion predictive models within the TCGA-KIRC cohort, employing the leave-one-out cross-validation (LOOCV) framework; (c) Rigorous validation of all resultant models ensued across four distinct validation datasets (CM-025, E-MTAB-1980, ICGC, and GSE167573); (d) Further refinement led to the determination of the optimal model, predicated upon a comprehensive comparison of Harrell's concordance index (C-index) across all validation datasets, thereby delineating the model manifesting the highest average C-index as the pinnacle.

### Immunotherapeutic and other therapeutic benefits

In exploring the immune-related characteristics of TTCRRS, the urothelial cancer dataset (IMvigor210) and the melanoma datasets (GSE91061, GSE78220) were stratified into four distinct treatment effects: stable disease (SD), progressive disease (PD), partial response (PR) and complete response (CR). Additionally, in the other melanoma dataset (GSE35640), patient cohorts were dichotomized into two distinct groups: non-responders (NR) and responders (R). Utilizing the expression profiles from both datasets, we computed the TTCRRS for each patient, subsequently delving into its implications on both prognosis and the efficacy of immunotherapy. To further explore the chemosensitivity of patients in different TTCRRS subtypes, we employed the pRRophetic R package 45, with this package, we were able to forecast the semi-inhibitory concentration (IC50) values—a widely recognized metric indicative of the efficacy of chemotherapeutic drugs—for everyone within the TCGA cohort. By comparing these IC50 values among different TTCRRS subtypes, we aimed to gain insights into the varying chemosensitivity patterns associated with TTCRRS.

### Nomogram construction and validation

To further popularize TTCRRS, we incorporated age, grade, stage, and TTCRRS in the TCGA cohort using the R package “rms” 49. Time-dependent receiver operating characteristic (time-ROC) analysis and C-index assessments were conducted to gauge the superior predictive accuracy and discriminatory capacity of the nomogram in contrast to other factors. This was executed leveraging the R packages “timeROC” 50 and “survcomp” 51. The calibration curve and decision curve analysis (DCA) were plotted by the “rms” package and “rmda” package 52 to reflect the consistency between the predicted endpoint events and the authentic outcomes at 1 year, 3 years, and 5 years. To assess the reliability of our nomogram, we evaluated the performance of the nomogram using another independent cohort.

### Analysis of CMAPs and docking of molecules

The Connectivity Map (CMAP), an open resource, leverages multiple gene expression profiles to establish connections among genes, small molecules, and diseases 53. In this process, Differential Expression Genes (DEGs) between high-TTCRRS and low-TTCRRS groups were scrutinized to identify molecules associated with our model. Subsequently, scores for each drug molecule were computed. Following this, molecular docking simulations were executed using the Lamarckian genetic algorithm to explore the relationship between CYP51A1 and these small molecules. The protein crystal structure of CYP51A1 was obtained from the RCSB Protein Data Bank (https://www.rcsb.org/), while the three-dimensional structures of all target compounds were sourced from the PubChem database (https://pubchem.ncbi.nlm.nih.gov/) in MOL2 format. Eventually, the molecular docking was performed utilizing AutoDock Vina and the outcomes were visualized through the Pymol 2.1 software.

### Culture and transfection of cells

The 786O cell line, representing human clear cell renal cell carcinoma (ccRCC), underwent cultivation and maintenance in RPMI-1640 medium (HyClone, Logan, UT, USA), complemented with 10% fetal bovine serum (FBS, Gibco, Carlsbad, CA, USA) and 1% penicillin-streptomycin solution, adhering to standard conditions of incubation at 37 °C in a 5% CO2 atmosphere. In our quest to elucidate the functional intricacies of CYP51A1 within ccRCC, we employed precise transfection methodologies to finely modulate CYP51A1 expression in 786O cells. This encompassed transfections with either negative control small interfering RNA (si-con) or distinct siRNA variants (siRNA-1/siRNA-2/siRNA-3), specifically targeting CYP51A1 for precise knockdown. Furthermore, cellular transfections incorporated both an empty overexpression vector and CYP51A1-overexpression plasmids (CYP51A*1*-OE). The transfection procedures meticulously adhered to Lipofectamine 3000 reagent (Invitrogen) protocols, meticulously executed for optimized efficiency. Through this rigorously controlled experimental paradigm, we orchestrated a deliberate modulation of CYP51A1 expression levels, affording us a comprehensive and nuanced exploration into its multifaceted functional ramifications within the intricate landscape of ccRCC.

### Extraction of total mRNA and real-time quantitative PCR

The isolation of total RNA sequences was achieved through the utilization of TRIzol reagent (Invitrogen, Carlsbad, CA) from both transfected and control cell samples. qRT-PCR reactions were conducted in triplicate using SYBR® Premix Ex Taq™ (Takara), following the manufacturer's recommended protocols. The primers specific to CYP51A1 were employed in this process, comprising forward primer 5'-GAAACGCAGACAGTCTCAAGA-3' and reverse primer 5'-ACGCCCATCCTTGTATGTAGC-3'. Post-qRT-PCR, the relative quantity of *CYP51A1* expression was determined through the 2-ΔΔCt calculation method, normalizing against GAPDH utilized as the internal standard.

### Isolation of proteins and western blot analysis

The extraction of proteins from 786O cells involved the use of RIPA lysis buffer (Beyotime Biotechnology Shanghai, China), followed by concentration utilizing the bicinchoninic acid protein assay kit (Beyotime Biotechnology, Shanghai, China). For protein analysis, samples underwent separation via electrophoresis on 6% or 10% SDS gels and were subsequently transferred to methanol-activated polyvinylidene fluoride (PVDF) membranes. These membranes underwent blocking with 5% bovine serum albumin (BSA) for 1 hour at room temperature and were then incubated overnight at 4 °C with primary antibodies, including anti-CYP51A1 (1:1000, AB210792, Abcam), anti-GAPDH primary antibody (1:5000, 4000-1-Ig, proteintech), anti-P105/P50 (1:1000, 13586, Cell Signaling Technology), anti-P65 (1:1000, 8242, Cell Signaling Technology), and anti-P-P65 (1:1000, 3033, Cell Signaling Technology). Following three washes with TBST, membranes were subjected to incubation with secondary antibody goat anti-rabbit IgG conjugated with HRP (1:5000, 96714, Cell Signaling Technology) at room temperature for 60 minutes. Subsequently, after three additional washes with TBST for 10 minutes each, the protein bands were visualized using ECL-plus™ western blotting chemiluminescence kits (BD Biosciences, NJ, USA).

### Assay for Cell Counting Kit-8 (CCK-8)

A seeding density of 1 × 10^3 786O cells per well was maintained in individual wells of 96-well plates. To gauge cell proliferation, the CCK-8 assay (Dojindo, Kumamoto, Japan) was employed, following the manufacturer's provided protocols, over a span of five consecutive days starting from day 1. In essence, 10 μL of CCK-8 solution was introduced into each well, followed by a 1-hour incubation period. Subsequently, the optical density (OD) of each well was measured at 450 nm using a spectrophotometer. Rigorous triplicate experimentation was undertaken to ensure robust statistical validation of the results.

### Transwell assay

A cell suspension containing 2 × 10^4 cells in 200 μL of FBS-free medium was seeded into the upper compartment of Boyden Transwell chambers (8 μm pore size, 24-well format; Corning Co., New York, NY, USA), which were then placed into a 24-well plate. The lower chamber was filled with culture medium supplemented with 10% FBS, serving as a chemoattractant. Subsequently, cells were allowed to migrate for 48 hours at 37 °C. Post-incubation, the cells residing on the upper surface of the membrane were meticulously wiped off, while those on the lower surface were fixed using a 4% paraformaldehyde solution. Staining with 0.1% crystal violet facilitated visualization and quantification of cells that had successfully traversed the membrane, enabling assessment of their migratory capacity.

### Assay for wound healing

ccRCC cells were evaluated for their migration ability in a wound-healing assay. The 786O cells were seeded into 6-well plates at 90% confluence after 48 h of transfection. Incubation and monolayer formation were performed on the cells. Using a 200 l Eppendorf tip, a wound was created by gently and mechanically scratching the cell monolayer. Following 24 hours of incubation, observations of the 6-well plates were made using a light microscope (400×; Nikon N-E; Nikon Corporation, Tokyo, Japan). Changes in scratch closure were quantified and compared among experimental groups utilizing ImageJ software, providing a comprehensive assessment of migration dynamics.

### Multiplex immunohistochemistry

Tissue samples, comprising normal tissues, RCC tissues, RCC-TT tissues, alongside associated clinicopathologic data from 197 patients, were obtained from the Fudan University Cancer Hospital and subsequently utilized to construct a tissue microarray. To unravel the relationship between the Tumor Microenvironment (TME), particularly focusing on CD8+ and CD4+ T cells, and the expression patterns of CYP51A1, we conducted multiplex immunohistochemistry (mIHC) utilizing anti-human CD4 (Abcam, ab133616), CD8 (Abcam, ab217344), and CYP51A1 (Abcam, AB210792) antibodies. This robust approach enabled a comprehensive exploration of the intricate interplay between immune cell populations and the signaling pathways of CYP51A1 within the TME.

### *In vivo* xenograft assay

All experimental procedures underwent prior approval by the Ethics Review Committee for Animal Experimentation at Fudan University. In this study, a cohort of 107 786O cells, categorized into si-CYP51A1, OE-CYP51A1, or control groups, were suspended in 100μl of PBS buffer and orthotopically implanted into the flanks of nude mice. After a 4-week observation period, humane euthanasia was performed on the mice, followed by the meticulous dissection and subsequent weighing of the *in vivo* solid tumors to enable comprehensive analysis.

### Chromatin immunoprecipitation (CHIP)

The CHIP assay was executed in accordance with the prescribed procedures delineated within the user manual for the EZ-Magna G Chromatin Immunoprecipitation kit (Millipore). Quantitative assessment of occupancy levels was performed via PCR analysis, comparing samples that underwent precipitation with a specific antibody against those subjected to precipitation with control immunoglobulin G (12-371; Millipore). The evaluation involved the utilization of specific primers, as outlined and specified in the corresponding SFigure 19.

### Statistical analysis

All data processing, visualizing, and statistical analysis were carried out using R 4.2.1 and Python 3.8.5. Continuous variables were compared using Student's t-test or Wilcoxon rank-sum test and the chi-squared test was adopted to compare categorical variables. Pearson's correlation was utilized to calculate correlations between two continuous variables. Samples were divided into two groups based on the optimal cut-off value of TTCRRS using the R package “survminer”. The Kaplan-Meier survival curves were established by the R package “survminer” and the difference in prognosis between the two groups was estimated by the two-sided log-rank test. Besides, the ComparedC package was performed to compare the C-indices of different clinical variables. The time-dependent AUC of survival variables was generated by the timeROC package. All the P-values were two-sided, and the results were regarded as statistically significant when the P-values were less than 0.05.

## Results

### TTCRG somatic alteration landscape

**Figure 1:**
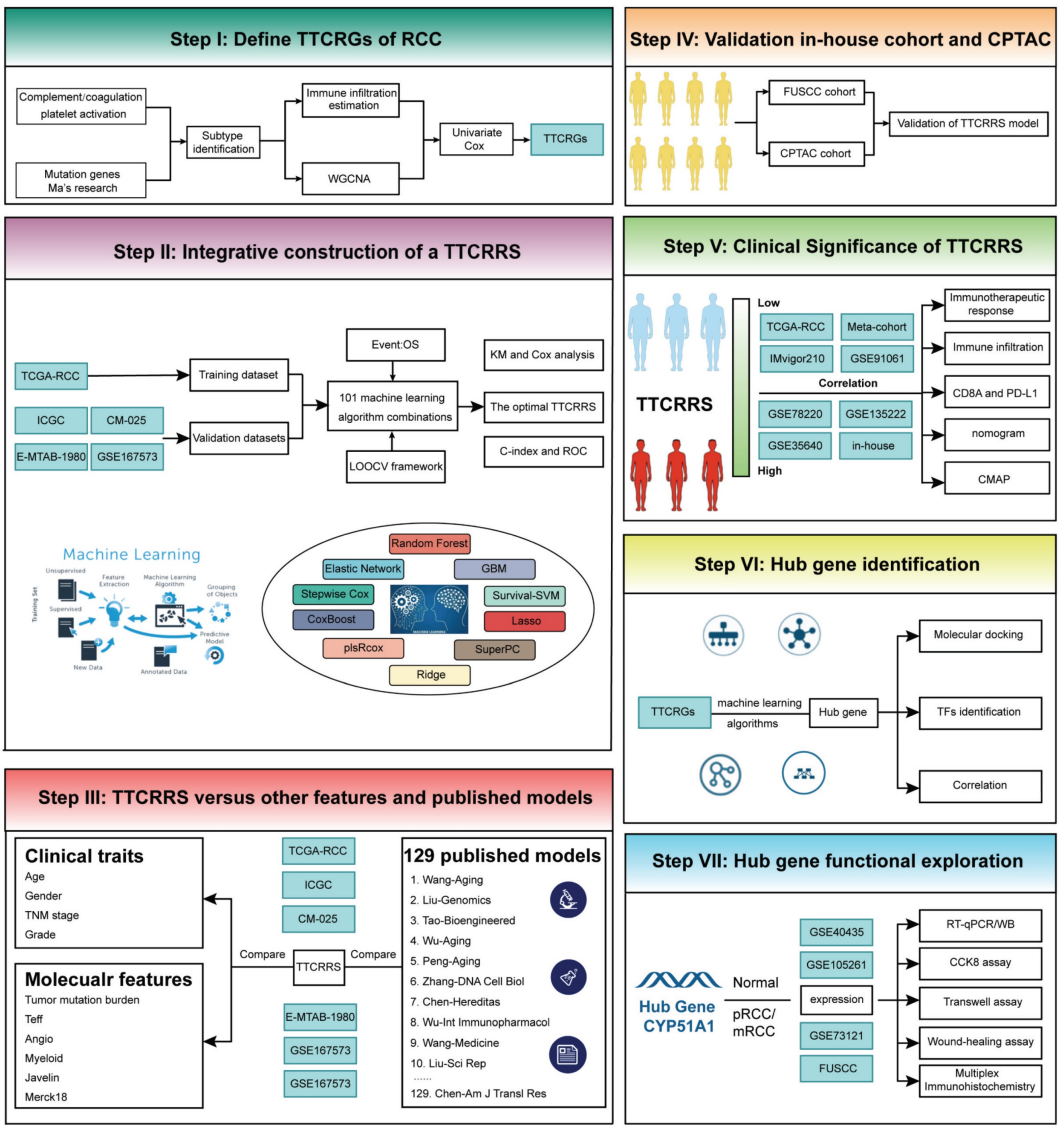
The workflow of our study.

**Figure 2:**
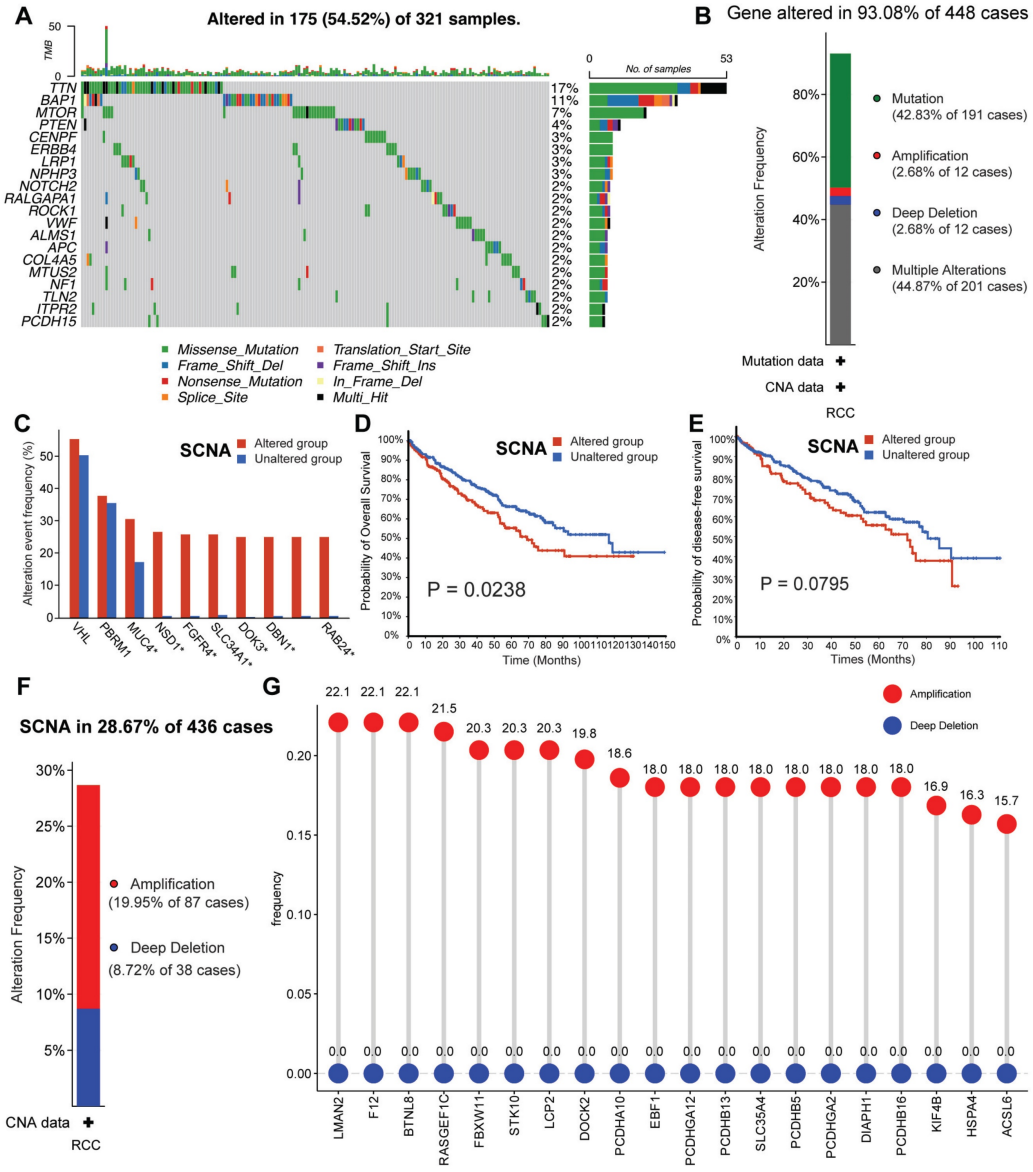
The genomic alterations of CRGs in RCC. (A) OncoPrint depicts the mutation profile of the top 20 frequently mutated genes. Each row represents a gene, and each column represents a patient. (B) Gene alteration frequency of RCC patients in TCGA cohort. (C) Histogram of the proportion of different SCNA mutation groups in RCC. (D-E) RCC patients' overall survival and relapse-free survival between SCNA altered and unaltered groups. (F) Histogram of the proportion of different SCNA types in RCC. (G) The lollipop chart displays the SCNA proportion of CRGs.

Our study's comprehensive design has been thoughtfully delineated in Figure 1 for readers' convenience. To elucidate the intricate genomic attributes of TTCRGs in RCC, an investigation into the SCNA and mutation frequency was undertaken utilizing data from 321 KIRC patients in the TCGA cohort. In examining these TTCRGs, it was observed that approximately 54.52% of patients exhibited DNA mutations within TTCRGs, showcasing an overall DNA mutation prevalence ranging from 2% to 17% (Figure 2A, B).

Predominant mutation styles of TTCRGs included Missense_Mutation, Frame_Shift_Del, Nonsense_Mutation, Translation_Start_Site. Notably, the top 10 TTCRGs with the highest mutation frequencies were *TTN* (17%), *BAP1* (11%), *MTOR* (7%), *PTEN* (4%), *CENPF* (3%), *ERBB4* (3%), *LRP1* (3%), *NPHP3* (3%), *NOTCH* (2%), and *RALGAPA1* (2%) (Figure 2A, Figure S1A). Kaplan-Meier survival analysis uncovered those patients with altered SCNA faced a poorer prognosis compared to those with unaltered SCNA (Figure 2D-E). However, statistical significance was not observed between high and low mutation groups (Figure S1C-D). Event frequencies pertaining to alteration in the top 10 TTCRGs based on mutation and SCNA data were depicted in Figure 2C and Figure S1B, respectively. Interestingly, within TTCRGs, the primary alteration types identified in the SCNA dataset were amplification and deep deletion (Figure 2F). Moreover, specific TTCRGs displayed distinct alteration patterns, with some genes showcasing amplification without deep deletions (*LMAN2*, *F12*, *BTNL8*, *RASGEF1C*,* FBXW11*,* STK10*, *LCP2*, and *DOCK*), while others exhibited deep deletions without amplifications (*ITPR1*, *BAP1*, *BRPF1*, and *ROBO2*) (Figure 2G). In conclusion, it was inferred that dysregulation and unfavorable prognosis of TTCRGs in RCC primarily stemmed from SCNA.

### Development, validation, and immune landscape of TTC subtypes

The tumor immune microenvironment constitutes a multifaceted ecosystem pivotal in both cancer progression and the response to immune therapies 54. In recent years, immunotherapy checkpoint inhibitors (ICI) have become a key component of treatment for many types of cancer. PD-1 blockade has significant transformative implications for the treatment of advanced ccRCC, and anti-PD-1 therapy is now a standard care option in both frontline and refractory treatment settings. As is well known, unlike other solid tumors, RCC is characterized by increased infiltration of immune cells and increased angiogenesis 55. In RCC, immune cells may experience specific metabolic dysregulation, characterized by impaired glucose uptake, glycolysis, and mitochondrial function 56. These metabolic impairments restrict immune cell activation and cannot be restored by PD-1 axis inhibition 56. Additionally, immune cells infiltrating RCC exhibit high heterogeneity in their activation status and cytotoxic potential. Therefore, regardless of the overall abundance of immune cells, the presence of specific subsets (such as stem-like TCF1+ or PD-1+ TIM-3- LAG-3- CD8+ T cell subsets) may be crucial for eliciting effective anti-tumor responses 57-58. Renal cancer thrombus, as a unique state, may exhibit different levels of immune cell infiltration compared to primary renal cancer. This could be attributed to distinct microenvironments within renal cancer thrombi compared to the primary tumor, leading to alterations in immune cell response and distribution. Thus, to further explore the immune cell infiltration between TCC subtypes, we first use some algorithms to appropriately subgroup in different cohorts.

**Figure 3:**
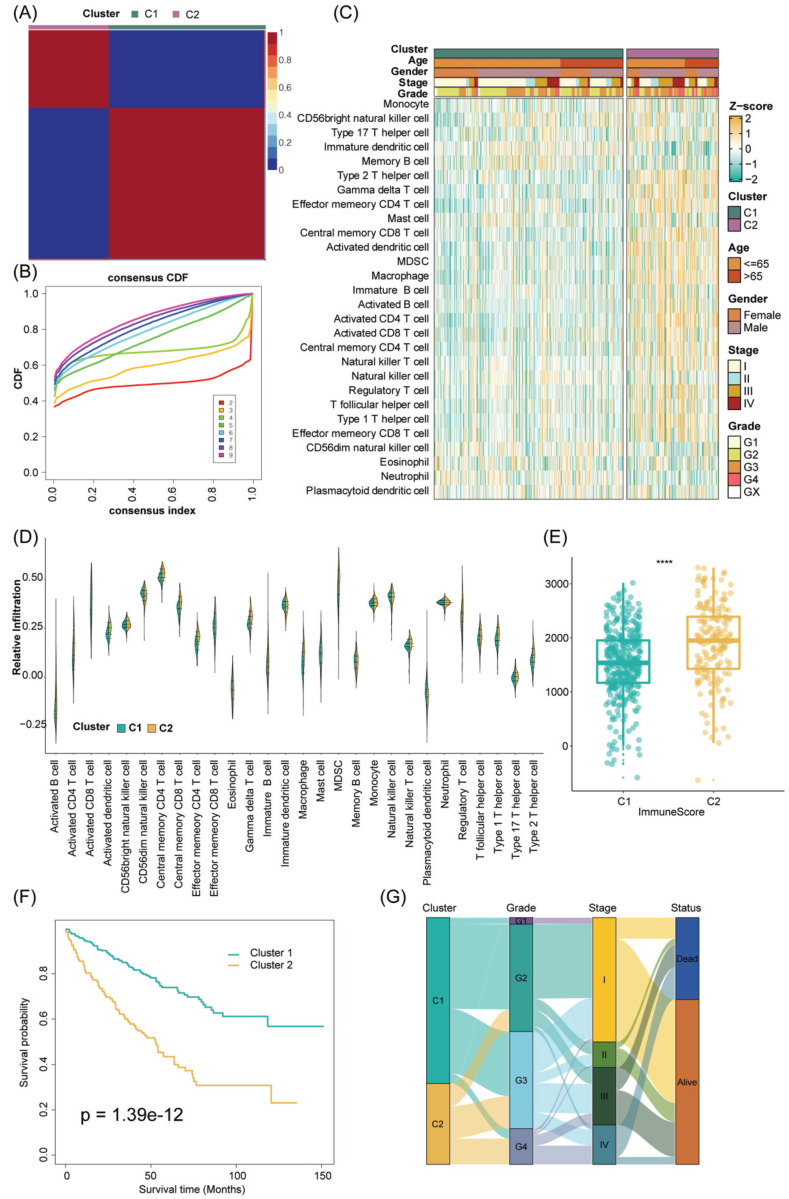
Consensus nonnegative matrix factorization of TTCRGs in RCC. (A) The consensus score matrix of all samples in the TCGA cohort for k = 2. A higher consensus score between two samples indicates they are more likely to be assigned to the same cluster in different iterations. (B) The CDF curves of the consensus matrix for each k (2-9) using the CNMF algorithm. (C) The heatmap shows the infiltration abundance of 28 immune cells calculated by ssGSEA between two clusters. Some clinical characteristics (TTC cluster, age, gender, stage and grade) were utilized as patient annotations. (D) The violin plot displays the distribution of 28 immune cell infiltration between two TTC clusters. (E) The box plot reveals the distribution of immune score inferred by the ESTIMATE algorithm between two TTC clusters. (F) Kaplan-Meier curve for OS between two TTC clusters in the TCGA cohort. (G) The alluvial diagram shows the changes in TTC clusters, grade, stage and status. **** means P < 0.0001.

In addition, to ensure the consistency and validity of our subtyping methodology, three clustering methods (CC, CNMF, and SNF-CC) were rigorously utilized to perform this procedure and their performance of them was evaluated with the following three criteria: (1) The significance of differences in survival profiles among subtypes can be assessed by a log-rank test of Kaplan-Meier curves; (2) Average silhouette width (ASW), which could reflect the coherence of clusters, to estimate whether samples are more parallel to subtypes; (3) clustering heatmap was generated to visualize the clustering effects of samples. The consensus clustering CDF curves in the three methods both indicated that the best number of clusters was 2 (Figure 3B, Figure S2E-H). Our results indicated that CNMF presented comprehensive advantages where cluster 1 consists of 352 patients and cluster 2 has 180 patients. All three clustering algorithms could clearly distinguish patients with survival information between two clusters (CNMF: P value = 1.39e-12, CC: P value = 4.08e-10, SNFCC: P value = 2.47e-08) (Figure 3F, Figure S2F-I). Then, for the ASW, the CNMF method outperforms the other two methods (CNMF = 0.99; CC = 0.66; SNFCC = 0.76). The figures, particularly Figure S2A-C, exhibit distinct demarcations between color blocks, each representing individual patients. Notably, the CC method illustrates pronounced block characteristics, while the SNFCC method maintains a moderate level of similarity within a cluster but tends to lack precision in defining boundaries (Figure 3A, Figure S2D-G). Remarkably, the two clusters demonstrated significant disparities in immune infiltration levels, notably with C2 exhibiting higher immune cell infiltration compared to C1 (Figure 3C, D). Hence, C1 was categorized as “tumor thrombosis immune cold” tumors, whereas C2 was deemed “tumor thrombosis immune hot” tumors. To ensure the integrity and consistency of the clusters, multiple algorithms (TIMER, quanTIseq, MCP-counter, xCell, EPIC) and diverse cohorts (ICGC, E-MTAB-1980, and CM-025) were employed to validate the robustness and stability of the ssGSEA results (Figure 3C-F, 4A-F). Furthermore, Figure 3G depicted the relationship between the two clusters and various clinical features, offering further insights into their distinct characteristics and potential clinical implications.

The stability and robustness of the subtypes (C1 and C2) were further verified by assessing the landscape of immune cell infiltration across different cohorts (ICGC, E-MTAB-1980, CM-025). This assessment involved the utilization of MCP-counter results to define the composition of the Tumor Microenvironment (TME) in RCC. The TME composition was characterized using MCP-counter Z-scores, and the expression levels of predictors associated with the functional orientation in the immune TME were compared between the two subtypes.

This comparison was conducted using Benjamini-Hochberg correction of two-sided Kruskal-Wallis tests' P-values. This rigorous analysis aimed to elucidate and validate the consistency of immune cell infiltration patterns across diverse cohorts and reaffirm the distinctions observed between the identified subtypes. Consistent with the observation from the TCGA results, the TME composition and immune infiltrates differ significantly between the two subtypes (C2 cluster, was characterized by the high expression of immune cells and immune-checkpoint-related genes) (Figure S3A-C). Analogously, tumor microenvironment deconvolution revealed that immunocyte infiltration was notably higher in C2 than C1 subtype (Figure S3D). Intriguingly, we also found DNA methylation of tumor-infiltrating lymphocytes (MeTILs) was also higher in C2 subtype compared with C1 subtypes, as depicted in the immune profile heatmap.

In a detailed investigation of pathway enrichment analysis between the two TTC clusters within the TCGA cohort, GSVA procedures revealed distinct expression patterns across 20 signaling pathways sourced from the KEGG database. Cluster 2 notably exhibited pronounced enrichment in cancer cell signal regulatory pathways such as the JAK-STAT signaling pathway, VEGF signaling pathway, MAPK signaling pathway, and p53 signaling pathway. Additionally, immune and inflammatory pathways including the T-cell receptor signaling pathway, B-cell receptor signaling pathway, nod-like receptor signaling pathway, and toll-like receptor signaling pathway showcased heightened enrichment in Cluster 2 (Figure S4A). Furthermore, GSEA analyses were conducted to delve deeper into the biological and clinical variances between the C1 and C2 clusters. These analyses revealed that DEGs between the clusters significantly manifested in pathways such as the IL-17 signaling pathway, NF-kappa B signaling pathway, Complement and coagulation cascades, and p53 signaling pathway (Figure S4B). Utilizing MeSH terms as the reference gene set in GSEA, DEGs were notably enriched in categories like Immunoglobulin G, Inflammation, Interleukin-10 receptor alpha subunit, and Immunologic receptors (Figure S4C, D). Subsequently, an exploration into the correlation between distinct clusters and histocompatibility and T-cell stimulators was undertaken. The findings unveiled higher expression levels of T-cell stimulators within the C2 cluster, whereas an inverse trend was observed in the major histocompatibility complex between the two clusters (Figure S4E, F). These intricate observations shed light on potential mechanisms underlying the differential biological and clinical characteristics between the identified clusters.

Subsequently, our exploration extended to evaluating potential associations between TTC subtypes and various pivotal indicators, including the TMB score, immune checkpoint molecules, TIDE score, and responsiveness to chemotherapeutic drugs. Notably, the expression levels of PD-1, PD-L1, CTLA4, LAG3, TIGIT, and HAVCR2 were markedly higher in the C2 subtype compared to the C1 subtype. Additionally, both the TMB score and TIDE score were notably elevated in the C2 subtype. These differences in gene expression between the two TTC subtypes were statistically significant according to the Wilcoxon test (P < 0.05) (Figure S5A-C). Moreover, when scrutinizing the response to specific chemotherapeutic drugs, distinct trends were observed. Patients in the C2 subtype exhibited significantly increased estimated IC50 values for Sorafenib, Gemcitabine, Vinorelbine, and Vorinostat (P < 0.05). Conversely, a reverse trend was observed for Gefitinib, Sunitinib, Cisplatin, and Vinblastine, where the estimated IC50 values were notably lower in C2 subtype patients compared to those in the C1 subtype (P < 0.05) (Figure S5D). These findings underscore the potential influence of TTC subtypes on immune characteristics and response to specific chemotherapeutic agents, offering insights into personalized therapeutic strategies.

### TTCRGs generated from WGCNA

In our Weighted Gene Co-expression Network Analysis (WGCNA), we determined the soft threshold β as 18, resulting in a scale-free R2 of 0.78 within the GSE48000 cohort. This parameter choice facilitated the construction of a scale-free network (Figure S6B). The sample dendrogram and trait heatmap of the VTE cohort were exhibited in Figure S6A. Subsequently, we identified a total of 14 gene modules represented by different colors within the GSE48000 cohorts, enabling cluster analysis (Figure S6C, D). Further scrutiny led to the selection of blue modules, demonstrating the highest correlation in the module-trait relationships, aiming to uncover genes associated with hypercoagulability (Figure S7A). Through scatterplots within the blue and cyan modules, we observed a high correlation coefficient of 0.64 between gene significance (GS) and module membership (MM), signifying the robustness and superior quality of our model construction (Figure S7B, P < 1e-200).

Conducting Wilcoxon test comparisons on genes within these pivotal modules in GSE48000, we identified statistically significant differences between normal patients and VTE patients (P = 0.00054 for GSE48000), signifying potential implications of these genes in the hypercoagulable state (Figure S7C). These findings signify a potential association between specific gene modules and the hypercoagulability observed in VTE patients, shedding light on underlying molecular mechanisms.

### Integrative construction of a TTCRRS using machine-learning

**Figure 5:**
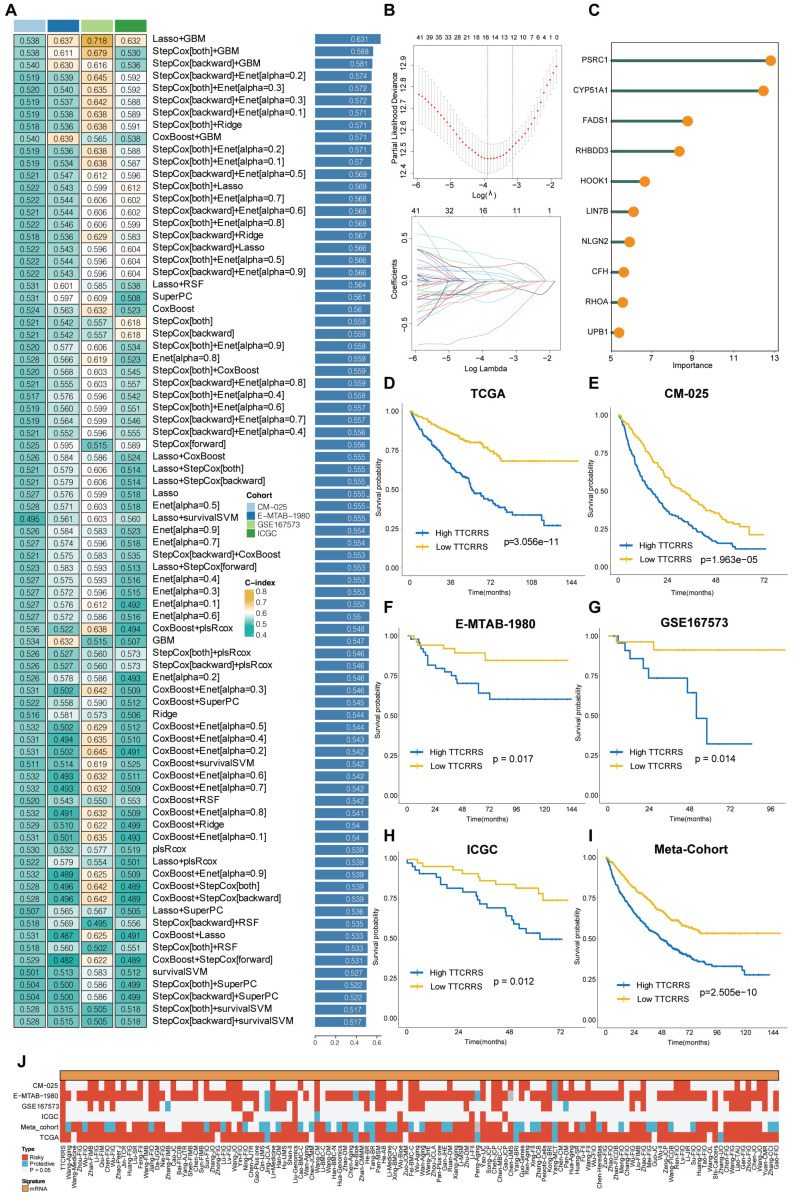
Development and validation of our TTCRRS by machine-learning integrative procedure. (A) 101 random combinations of 10 machine-learning algorithms by a 10-fold cross-validation framework. The C-index of each model was further calculated across all validation cohorts (ICGC, GSE167573, CM-025 and E-MTAB-1980). (B) The LASSO regression lambda filter. The optimal λ was generated when the partial likelihood of deviance reached the minimum value and the corresponding LASSO coefficients of each TTCRG were also obtained. (C) The coefficients of the top 10 TTCRGs were further filtered via the GBM machine-learning algorithm. (D-I) Kaplan-Meier curves of OS between patients with high-TTCRRS signature and low-TTCRRS signature in the TCGA-KIRC cohort, CM-025 cohort, E-MTAB-1980 cohort, GSE167573 cohort, ICGC and Meta-cohort. (J) Univariate Cox regression analysis of TTCRRS signature and other 129 signatures in multiple cohorts.

Our study embarked on the development of a prognostic TTCRRS by initially performing univariate Cox regression analysis for Overall Survival (OS), which led to the identification of 45 crucial TTCRGs within the TCGA cohort (Figure S7D). Subsequently, these 45 TTCRGs were integrated into a machine learning-based framework that employed 101 random combinations with 10 algorithms based on a Leave-One-Out Cross-Validation (LOOCV) methodology within the TCGA-KIRC cohort. This process aimed to derive the most robust TTCRRS, optimizing for the highest C-index across multiple validation cohorts (CM-025, EMTAB-1980, GSE167573, ICGC) (Figure 5A). Evidently, the optimal model, yielding a superior C-index of 0.631 across all validation cohorts, emerged through the combination of the LASSO and GBM algorithms (Figure 5A) and the coefficients of GBM was displayed in Table S2. The LASSO regression method identified the optimal sparseness parameter λ, minimizing the partial likelihood deviance through 10-fold cross-validation with 1000 repeats (Figure 5B). Subsequently, the GBM algorithm was leveraged to determine hyperparameter values essential for constructing a high-performance TTCRRS, leading to the identification of a final set of 10 TTCRGs (Figure 5C). To stratify patients based on risk, the corresponding equation is as following:



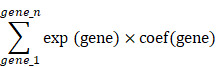



This facilitated the segregation of patients into high- and low-risk groups using the optimal cutoff value in all cohorts (Figure S8A-F). Remarkably, the high-risk group consistently exhibited poorer Overall Survival (OS) compared to the low-risk group across multiple cohorts, including the TCGA-KIRC cohort, four validation cohorts, and the Meta-cohort (a combination of all samples) (all P < 0.05) (Figure 5D-I). These findings underscore the predictive potential and clinical relevance of the derived TTCRRS in stratifying RCC patients based on survival outcomes.

The predictive accuracy of our TTCRRS model was assessed through the average Area Under the Curve (AUC) values for 1-year, 3-year, and 5-year Overall Survival (OS) across diverse cohorts. In the TCGA-KIRC cohort, the average AUC values were 0.749, 0.718, and 0.737 for 1-year, 3-year, and 5-year OS, respectively. Conversely, the ICGC cohort displayed lower values at 0.522, 0.646, and 0.646 for the same OS intervals. The CM-025 cohort yielded AUC values of 0.664, 0.617, and 0.617, while the E-MTAB-1980 cohort showed values of 0.704, 0.792, and 0.792. In the GSE167573 cohort, the AUC values were 0.693, 0.884, and 0.884, whereas the Meta-cohort presented values of 0.671, 0.635, and 0.635 (Figure S9A). Concurrently, the Harrell's C-index was calculated across these cohorts, demonstrating values of 0.702 (95%CI: 0.611-0.780), 0.664 (95%CI: 0.459-0.821), 0.596 (95%CI: 0.511-0.675), 0.753 (95%CI: 0.519-0.896), 0.784 (95%CI: 0.378-0.956), and 0.614 (95%CI: 0.558-0.667) in the respective cohorts (Figure S9B). Moreover, comparisons were made with the C-index of clinical characteristics displayed in Figure S9C. These indices collectively emphasize the stability and robustness of TTCRRS as a predictor across multiple independent cohorts, suggesting its potential clinical utility as a reliable prognostic tool.

### Comparison of TTCRRS in RCC

**Figure 6:**
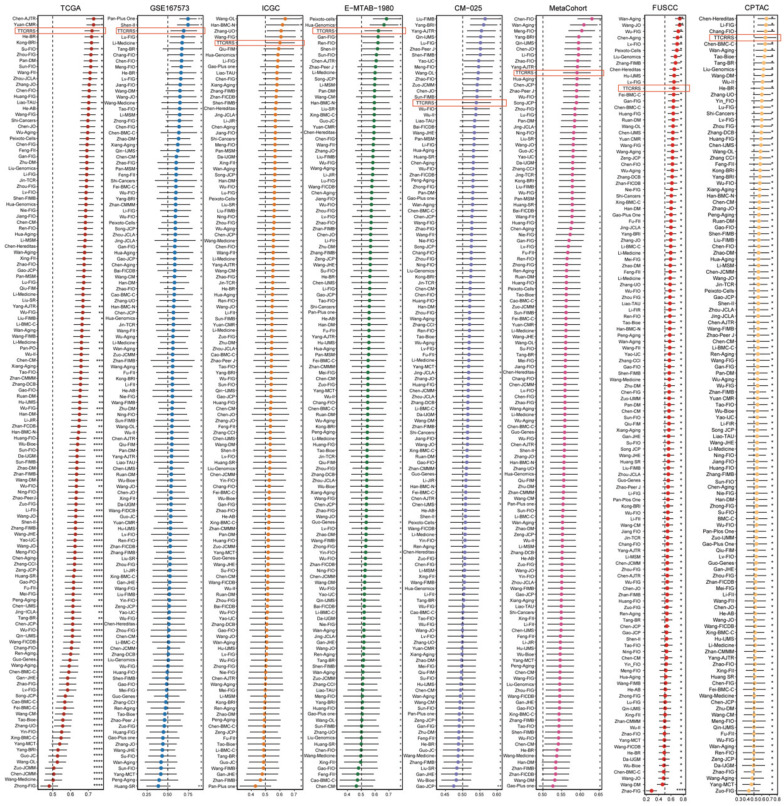
C-indexes of TTCRRS and 129 published signatures in training cohorts (TCGA-KIRC, GSE167573, ICGC, E-MTAB-1980, CM-025, Meta cohort) and test cohorts (FUSCC and CPATC cohort).

In recent years, advancements in next-generation sequencing technologies have led to the discovery of numerous gene expression-based signatures using machine learning approaches. In our study, we compiled a comprehensive comparison involving 129 signatures from published literature spanning the last three decades (Table S3). These signatures were intricately linked to various biological processes, encompassing immune infiltration, DNA methylation, metabolism, autophagy, tumor microenvironment, epithelial-mesenchymal transition, pyroptosis, hypoxia, glycolysis, immunotherapy response, telomere biology, ferroptosis, and necroptosis. Through univariate Cox regression analyses across all datasets for each signature from the published literature, our model, the TTCRRS, consistently demonstrated a significant correlation with prognosis in all datasets (Figure 5J). This comprehensive assessment further validates the robustness and reliability of our model in predicting patient outcomes. Furthermore, when comparing the Harrell's C-index of TTCRRS with other models, our TTCRRS consistently outperformed most of the other models across all cohorts (Figure 6) (Table S4).

This observation underscores the superior performance of the TTCRRS in prognostication when compared to numerous established models from existing literature, highlighting its potential as a highly effective prognostic tool across diverse datasets.

### TTCRRS validated in proteomics cohorts

**Figure 7:**
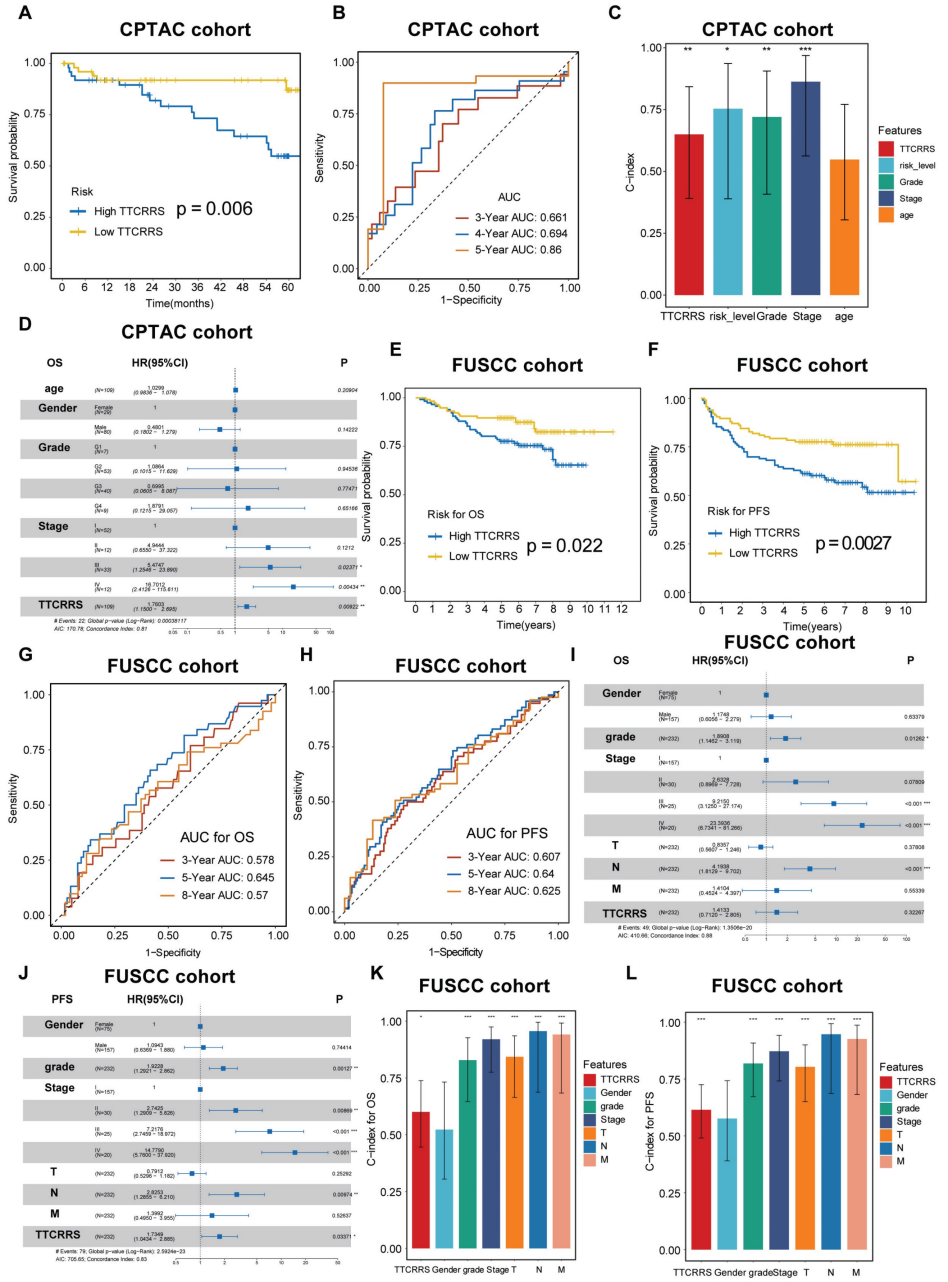
Validation of TTCRRS signatures in the protein cohorts (CPTAC and FUSCC cohort). (A) Kaplan-Meier curve of OS between high TTCRRS and low TTCRRS in the CPTAC cohort. (B) Time-dependent ROC analysis for predicting OS at 1, 3 and 5 years in the CPTAC cohort. (C) The predictive performance of TTCRRS when compared with traditional clinical variables to predict OS at the protein level is based on the CPTAC cohort. (D) The forest plot illustrates the Multivariate Cox regression analysis of OS in the CPTAC cohort. (E-F) Kaplan-Meier curve of OS and PFS between high TTCRRS and low TTCRRS in our in-house cohort. (G-H) Time-dependent ROC analysis for predicting OS and PFS at 3, 5 and 8 years in our in-house cohort. (I-J) The forest plot illustrates the Multivariate Cox regression analysis of OS and PFS in our in-house cohort. (K-L) The predictive performance of TTCRRS when compared with traditional clinical variables to predict OS and PFS at the protein level is based on our in-house cohort.

To further test the performance of our model in protein expression, we used two proteomics databases (CPTAC and FUSCC cohorts) to calculate TTCRRS. Kaplan-Meier analysis in CPTAC and FUSCC cohorts showed that patients with the low TTCRRS subtype had a much better prognosis than those with the high TTCRRS subtype (Figure 7A, E, F). The AUCs of the TTCRRS for OS were 0.661 at 3 years, 0.694 at 4 years, and 0.86 at 5 years for the CPTAC cohort (Figure 7B). Consistently, the AUCs of the TTCRRS for the FUSCC cohort (in-house cohort) were 0.578 at 3 years, 0.645 at 5 years, and 0.57 at 8 years with OS prognosis (Figure 7G), and 0.607 at 3 years, 0.64 at 5 years, and 0.625 at 8 years with PFS prognosis (Figure 7H). Similarly, the C-index for OS in the CPTAC cohort reached 0.649 (0.391-0.843) (Figure 7C), and in the FUSCC cohort, the C-index of OS and PFS was 0.600 (0.446-0.738) and 0.614 (0.490-0.725) (Figure 7K-L). In addition, we conducted multivariate Cox regression analyses to determine whether the TTCRRS was a clinically independent prognostic factor for RCC patients in proteomics cohorts. The results in the forest plot were visualized in (Figure 7D, I, J) implied that the TTCRRS could be served as an independent factor based on the expression of protein data. Collectively, although there was some degree of the predictive power of TTCRRS in proteomics data, obviously, our model had a superior accuracy in transcriptomic data.

### Implications of TTCRRS for immunotherapy, chemotherapy, and biological mechanisms

**Figure 8:**
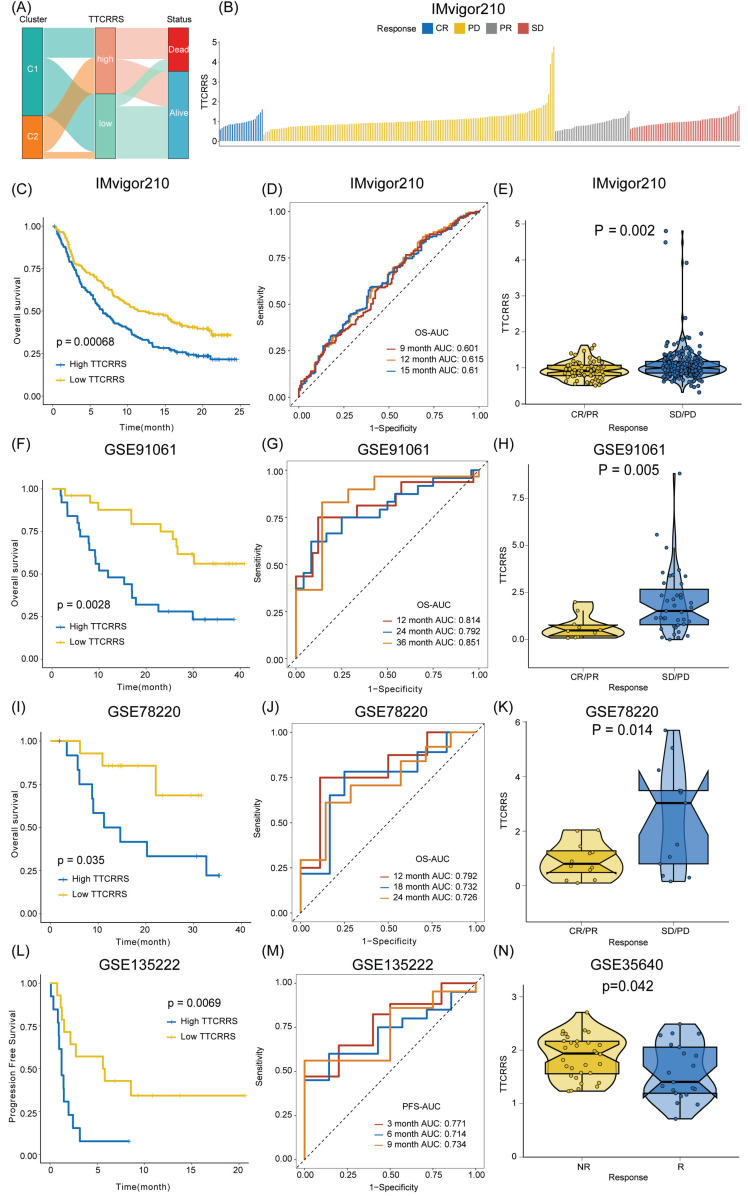
Immune landscape of the TTCRRS signatures. (A) Sankey plot visualized the relationships among the TTC clusters, TTCRRS and survival status. (B) Waterfall plot illustrates the distribution of TTCRRS for patients exhibiting different immunotherapeutic responses in the IMvigor210 cohort. (C, F, I) Kaplan-Meier curve analysis of OS between high TTCRRS and low TTCRRS in the IMvigor210, GSE91061, and GSE78220 cohorts. (D, G, J) Time-dependent ROC analysis for predicting OS in the IMvigor210, GSE91061, and GSE78220 cohorts. (E, H, K, N) Boxplot displays the TTCRRS signature in patients with different immunotherapy responses in the IMvigor210, GSE91061, GSE78220, and GSE35640 cohorts. (L) Kaplan-Meier curve analysis of PFS between high TTCRRS and low TTCRRS in the GSE135222 cohort. (M) Time-dependent ROC analysis for predicting PFS in the GSE135222 cohort. Significance: * < 0.05, ** < 0.01, *** < 0.001.

**Figure 9:**
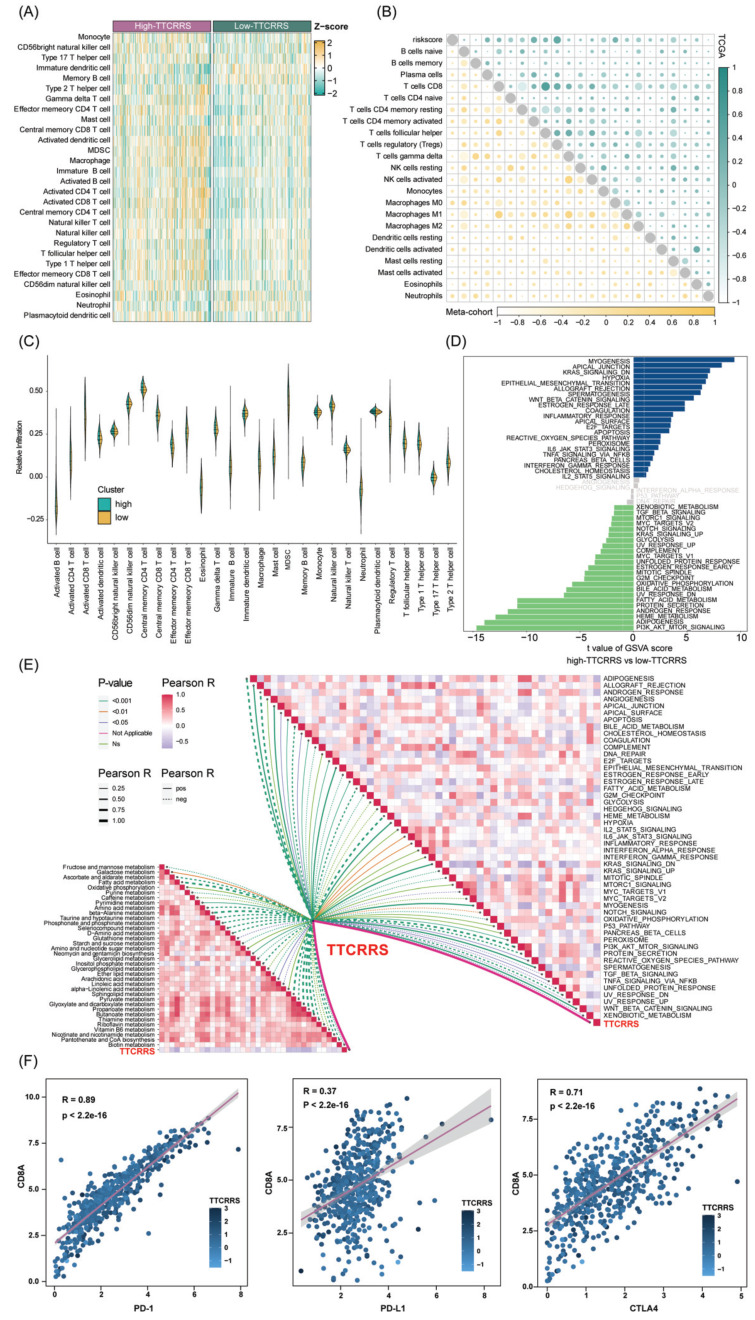
Function annotation of the TTCRRS signature based on the TCGA cohort. (A) The relationship between TTCRRS and 28 immune cell infiltrations. (B) Chorogram plot depicts the Pearson r value between TTCRRS and immune cell infiltrations in TCGA and Meta cohorts. (C) Violin plot shows the relationship between 28 immune cell infiltrations and the TTCRRS subtype. (D) Difference in pathway activities scored per patient by GSVA between high- and low-TTCRRS. Shown are t values from a linear model. (E) Butterfly plot illustrates the correlation between the TTCRRS and metabolic pathways, the enrichment pathways based on GSVA of GO and KEGG terms. (F) Scatterplots between CD8A and PD0-1, PD-L1, and CTLA4 with the TTCRRS were shown in the TCGA-KIRC.

Accumulating evidence has revealed that cancer immunotherapy has made striking progress, and revolutionized the paradigms of cancer therapy, which has been recognized as a promising therapeutic frontier 59-63. Herein, we further assessed the predictive value of TTCRRS for immunotherapy response. However, published cohorts of RCC patients who received immunotherapy were limited. Instead, the urothelial cancer cohort (IMvigor210) that received anti-PD-L1 therapy, the malignant melanoma cohort (GSE91061) that received anti-PD-1 and anti-CTLA4 therapy, the pre-treatment melanomas cohort (GSE78220) undergoing anti-PD-1 checkpoint inhibition therapy, the advanced non-small cell lung carcinoma cohort (GSE135222) that treated with anti-PD-1/PD-L1, and the metastatic melanoma cohort (GSE35640) that received antigen-specific cancer immunotherapy were enrolled. The Sankey plot displayed the correlation of TTCRRS, cluster, and survival status. The high-risk group possesses a higher proportion of patients with mortality outcomes than those alive (Figure 8A). With the same formula, patients in these cohorts were also stratified into high or low risk subtypes in the light of the optimal cutoff generated from the training and validation cohorts, respectively. Kaplan-Meier analysis demonstrated that patients with the high-risk subtype had a much worse prognosis than those with the low-risk subtype in the IMvigor210 cohort (Figure 8C), the GSE91061 cohort (Figure 8F), the GSE78220 cohort (Figure 8I) and the GSE135222 cohort (Figure 8L) (all P < 0.05). Meanwhile, the AUCs of our model for survival prognosis were 0.601 at 9 months, 0.615 at 12 months, and 0.61 months at 18 months for the IMvigor210 cohort (Figure 8D), 0.814 at 12 months, 0.792 at 24 months, and 0.851 at 36 months for the GSE91061 cohort (Figure 8G), 0.792 at 12 months, 0.732 at 18 months, and 0.726 at 24 months for the GSE78220 cohort (Figure 8J), 0.771 at 3 months, 0.714 at 6 months, and 0.734 at 9 months (Figure 8M). For each cohort with response to immunotherapy, attention must be paid to the boxplots, which can be seen that the TTCRRS values were significantly elevated in patients with SD or PD when compared with those with CR or PR (P = 0.002 for IMvigor210 cohort, P = 0.005 for GSE91061cohort, P =0.014 for GSE78220 cohort) (Figure 9E, H, K). Of note, patients in the GSE35640 cohort with low risk were more likely to respond to antigen-specific cancer immunotherapy (Figure 8N). The predictive value of the TTCRRS values to immunotherapy was also verified by the waterfall plots in the IMvigor210 cohort (Figure 9B). By comparing the IC50 values between two TCCRRS subtypes, we realized that patients in high-risk subtype were sensitive to Gefitinib (P=4.1e-08), Sunitinib (P<2e-16) and Vinblastine (P < 2e-16), whereas the low-risk subtype was sensitive to Gemitabine (P=1.4e-05), Sorafenib (P=5.3e-10) and Vinorelbine (P=1.2e-06) (Figure S10).

We then investigate the differences in the immune characteristics between the high and low risk subtypes. Cell infiltration analysis showed a remarkably positive correlation between TTCRRS and immune infiltration abundance in both the TCGA-RCC and Meta-cohort (Figure 9A, B, Figure S11A). As a labeled molecule in killer T cells, there was a predominant association between *CD8A* and *PD-1*, *PD-L1* and *CTLA4* in the TCGA cohort and Meta cohort (TCGA cohort: r = 0.89 with PD-1, r = 0.37 with PD-L1, r = 0.71 with CTLA4, all P value < 2.2e-16; Meta cohort: r = 0.95 with PD-1, r = 0.47 with PD-L1, r = 0.64 with CTLA4, all P value < 2.2e-16) (Figure 9F, Figure S11B). Likewise, the scatter plot of TTCRRS and *CD8A, PD-1, CTLA4* also illustrated a positive correlation (*CD8A*: r = 0.13, P = 2.51e-03; *CTLA4*: r = 0.26, P = 1.26e-09; *PD-1*: r = 0.24, P = 1.78e-08) (Figure S12A, C, D). Interestingly, a negative correlation between TTCRRS and PD-L1 was found in Figure S12B (r = -0.12, P = 5.07e-03). We also investigated some signatures that can activate immune responses 64-69, the expression levels of *CXCR3, CCL5, LAG3, IFNG, TIGIT* and *ZNF683* were higher in the high-risk subtype compared with the low-risk subtype (all P < 0.05) (Figure S12E-J). Of note, strong positive associations were also found between TTCRRS values and these signatures indicated that the increased TTCRRS values were correlated with the increased antitumor immune response (Figure S12K-P).

Meanwhile, the distribution of tumor immune cell infiltrations by these two TTCRRS subtypes was visualized in the boxplot (Figure 9C, Figure S11C). The high-risk subtype had significantly higher proportions of activated dendritic cell, natural killer cell, CD4 T cell, CD8 T cell, B cell et al. To better understand immune cell infiltration between two subtypes, we collected 181 immune cell signatures from diverse research through an extensive literature search 70-74 (Table S5). Similarly, we also found that high-risk patients tended to have more immune cell signatures, which is consistent with the above results (Figure S13-16). Above funding implied that patients with high-risk owned more backup resources for immunotherapy.

GSVA was performed to elucidate which pathways were enriched and depleted between two subtypes. (Figure 9D, Figure S11D) highlighted those related genes highly expressed in the high-risk subtype showed significant enrichment in multiple cancer-related pathways such as IL6-JAK-STAT3 signaling, Wnt-β-catenin signaling, Kras signaling and IL2-STAT5 signaling based on the TCGA cohort and Meta cohort. Subsequently, some immunotherapy-predicted pathways were performed to investigate the correlations between TTCRRS and the predicted immune checkpoint blockade (ICB) response signatures. According to the results of the TCGA cohort and Meta cohort, we found TTCRRS was positively correlated with the enrichment scores for most immunotherapy-predicted positive signatures (Figure 9E, Figure S11E). As expected, the association between TTCRRS and enrichment pathways based on GSVA algorithm was also visualized in (Figure 9E, Figure S11E).

### TTCRRS for nomogram construction

**Figure 10:**
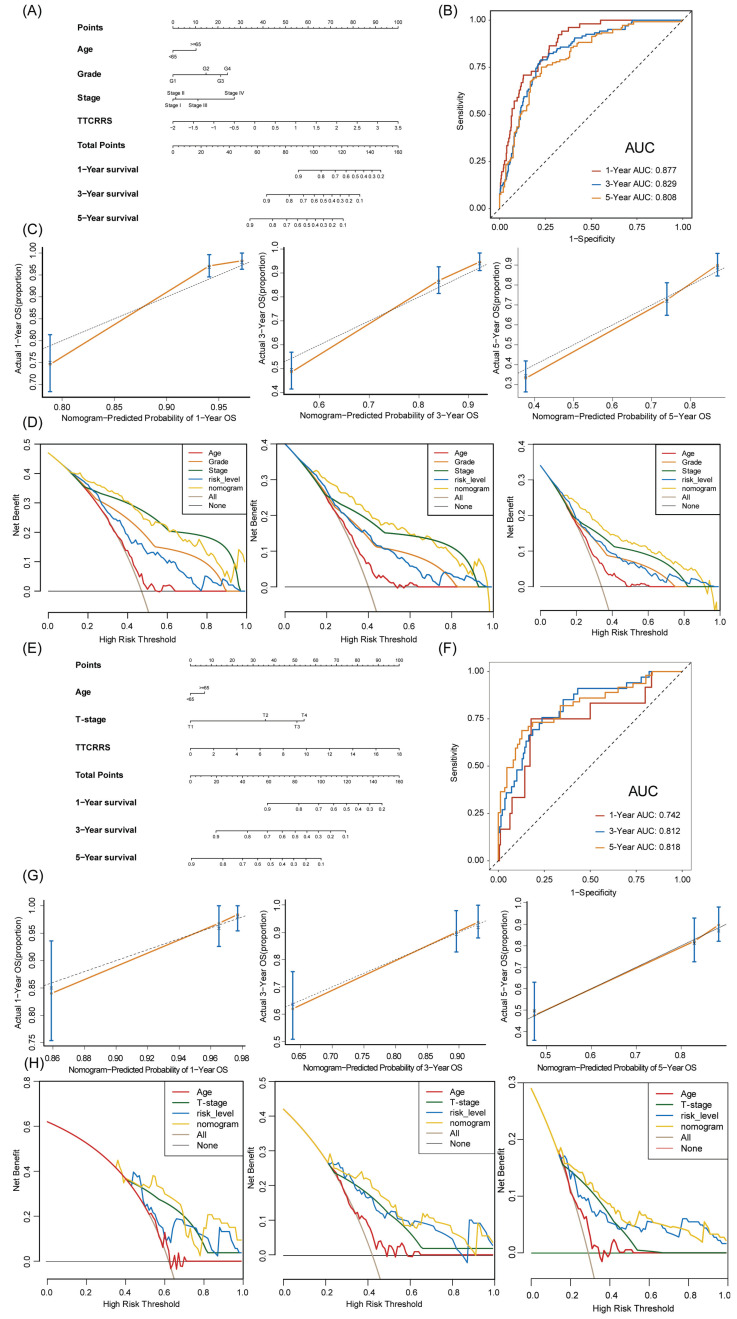
Nomogram development and validation based on TTCRRS. (A, E) Nomogram for predicting probabilities of RCC patients with 1-, 3-, and 5-year OS outcomes in the training (TCGA) and validation cohorts (Meta-cohort). (B, F) Time-dependent ROC curve at 1, 3 and 5 years in the training cohort and validation cohort. (C, G) The calibration plots for predicting RCC patients with 1, 3, and 5-year OS in the training and validation cohorts. The nomogram's ideal performance is shown by the dashed lines. (D, H) The decision curve analysis of nomogram and other clinical factors for 1, 3, and 5-year risk. Black line represents the hypothesis that no patient died after 1-, 3-, and 5-years.

To provide doctors with a quantitative tool to predict the prognosis of RCC patients with tumor thrombosis, a nomogram that integrated TTCRRS and other clinical information was constructed and validated in the training cohort (TCGA dataset) and validation cohort (Meta dataset). Then, a TTCRRS for patients was calculated to predict an individual's 1-, 3-, and 5- overall survival based on the nomogram. It is noteworthy that the TTCRRS was found to contribute the most risk points when compared with other clinical characteristics (Figure 10A, E). The AUC of our nomogram model in the training cohort and validation cohort for predicting 1-, 3- and 5-year were 0.877, 0.829, 0.808, 0.742, 0.812, 0.818, respectively (Figure 10B, F). The C-indices of TTCRRS in the two cohorts both showed the optimum performance of the nomogram (TCGA cohort: 0.792, 95%CI: 0.766-0.827; Meta cohort: 0.780, 95%CI: 0.717-0.844). Besides, the calibration curves of the training cohort and validation cohort were illustrated in (Figure 10C, G). The calibration curves fitted well with the ideal curves (the idealized 45° line) in the training cohort and validation cohort, especially for the calibration curves of 5-year overall survival. Furthermore, the nomogram displayed a higher net benefit for decision curves than other clinical factors to predict the overall survival probability (Figure 10D, H).

### Identification of hub gene and TF, CMAP analysis and molecular docking

**Figure 11:**
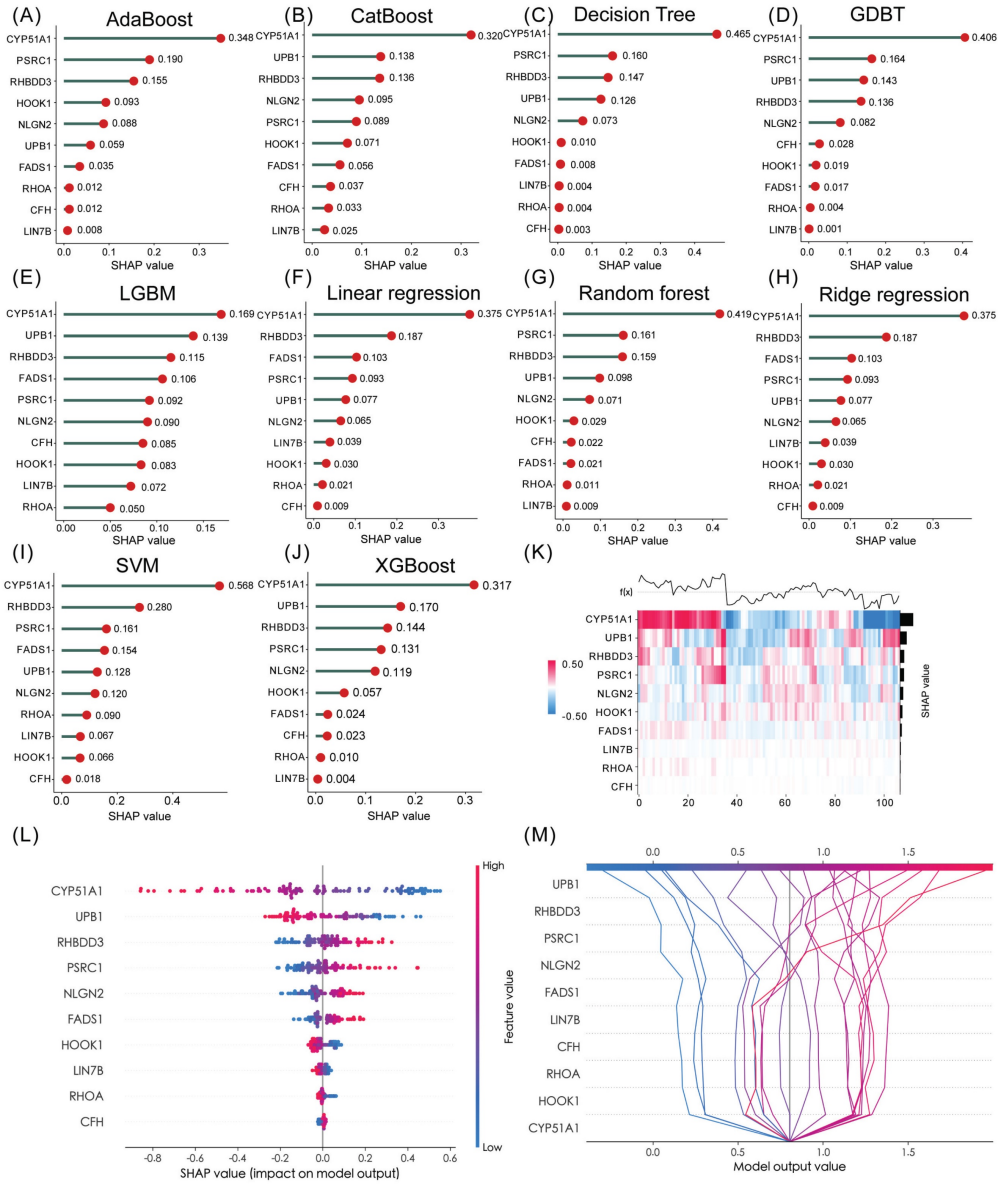
Hub gene identification via 10 machine-learning algorithms. (A-J) Feature importance ranking within our TTCRRS signature using 10 machine-learning algorithms (AdaBoost, CatBoost, Decision Tree, GDBT, LGBM, Linear regression, random forest, Ridge regression, SVM and XGBoost). (K) A heatmap plot of the SHAP values for the top 10 features in the training cohort. (L) The summary plot shows how a feature's effect on the output changes with its own value. For each feature, high values are shown in red and low values are in blue. For example, it appears that CYP51A1 is positively and negatively correlated with the log odds of TTCRRS. Features are ordered on the y-axis by their average SHAP importance value across the three classes. (M) SHAP decision plot for TTCRRS shows how the model's prediction was made for a single observation. Each line represents the log odds for a single class. The features are on the y-axis and sorted by the average SHAP value for that observation. The lines intercept the top x-axis at their final log odds value. The maximum log odds value class is used as the model's output.

In the realm of predictive modeling, machine learning methods have gained significant attention due to their ability to handle diverse features. To gain a deeper understanding of the features employed by our predictive model, we utilized SHAP (Shapley Additive Explanation) values, a Python-based “model interpretation” package, that can be used to interpret the output of a machine-learning model and quantify the contribution of each feature to the model's prediction results. To this end, we determined the optimal features for 10 machine-learning algorithms (Adaboost, Catboost, Decision Tree, GDBT, LGBM, Linear regression, Random Forest, Ridge regression, SVM and XGBoost) and ranking approaches. Figure S17A-J and Table S6 showed the accuracy of 10 machine-learning algorithms. Among them, SVM emerged as the best algorithm for our TTCRRS model, exhibiting excellent prediction performance. When examining the ten TTCRGs, the SHAP values derived from the model interpretation using the ten machine learning algorithms demonstrated that all these features had a positive impact on the predictions. Notably, CYP51A1 emerged as the feature with the most substantial contribution to the predictive model (Figure 11A-J). Using supervised clustering based on the SHAP value, samples with the same model output were clustered together, for example, samples with a global effect on CYP51A1 were pooled together (Figure 11K). Figure 11L showed the resulting feature-baseda SHAP summary plot and the top score with the highest SHAP feature importance was CYP51A1. In the SHAP decision plot (Figure 11M), CYP51A1 possessed the lowest error rate in the predictive model.

As a part of our effort to identify potential small molecules related to our TTCRRS, we performed a CMAP analysis. Then, the Table S7 below lists ten small-molecule drugs with highly significant correlations. Next, ten machine-learning algorithms docked these 10 compounds with the top scores for SHAP features (CYP51A1) (Table S8). Based on their binding energy of -5 kcal/mol, both compounds showed a high affinity for CYP51A1. In addition, the top five high-affinity compounds combined with CYP51A1 were also visualized in 3-D (Figure S19A).

To examine transcription factors that might affect CYP51A1 gene transcription in further detail, we examined factors of potential molecular networks that may affect CYP51A1 expression (Figure S18A). Subsequently, we figured out whether some significant correlation exists between these TFs and CYP51A1. Most TFs exhibited a strong correlation with CYP51A1 (BRD4: r = 0.37, P < 2.2e-16; SMAD2: r = 0.58, P < 2.2e-16; POLR2A: r = 0.59, P < 2.2e-16; CHD7: r = 0.44, P < 2.2e-16; SUZ12: r = 0.62, P < 2.2e-16; TRRAP: r = 0.68, P < 2.2e-16 (Figure S19B). After that, to investigate the biological function of CYP51A1, the single-cell database “CancerSEA” was adopted. Functional relevance analysis revealed a positive correlation between CYP51A1 expression and hypoxia (r = 0.56, P < 0.001) (Figure S18B, C).

### CYP51A1 regulates the proliferation and migration properties of RCC *in vitro* and *in vivo*

**Figure 12:**
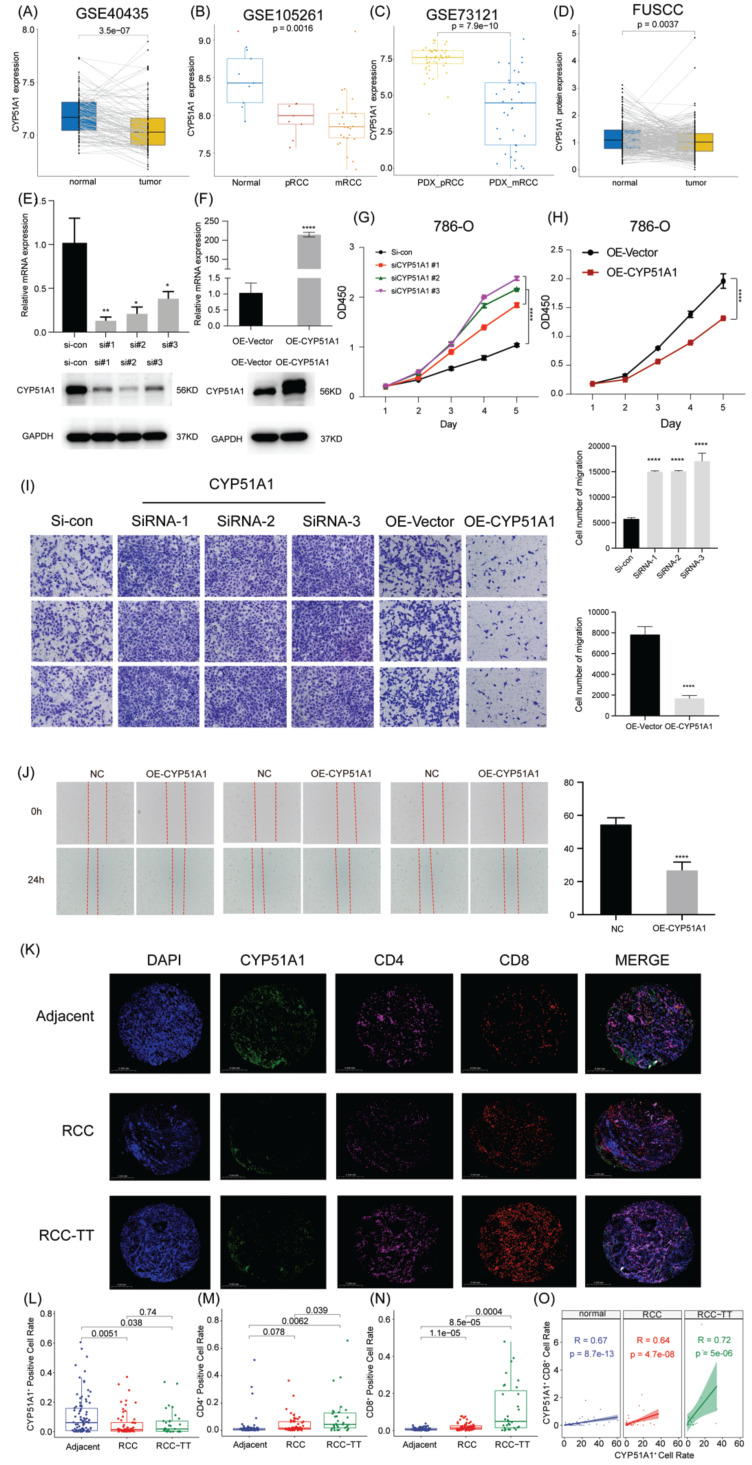
CYP51A1 inhibits the proliferation and migration capacities of ccRCC cells. (A) CYP51A1 expression profiling of 101 pairs of ccRCC patients based on the GSE40435 cohort. (B) CYP51A1 expression of normal, primary ccRCC and metastatic ccRCC patients based on the transcriptome profiling GSE105261 cohort. (C) CYP51A1 expression profiling of PDX-pRCC and PDX-mRCC based on the GSE73121 cohort. (D) The difference in CYP51A1 protein levels between normal and tumor tissues. (E-F) Western blotting and RT-qPCR assay of CYP51A1 expression in transfected 786O cell lines. (G-H) CCK-8 assays showed the effect of CYP51A1 knockdown (g) and overexpression (h) on the proliferation of 786O cells. (I) Representative images of the migration assays performed using 786O cells. Scale bars, 100 μm. (J) *In vitro* scratch migration assay in transfected 786O cell lines. (K) The expression of CYP51A1, CD4 and CD8 molecules in tissue detected by multiplex immunohistochemistry. (L-N) The quantification of CYP51A1, CD4 and CD8 signals. (O) The correlation of CYP51A1 and CD8 molecules. Significance: * < 0.05, ** < 0.01, **** < 0.0001.

Subsequently, we explored the expression pattern of CYP51A1 across different tissues. We found the expression of CYP51A1 was downregulated in tumor tissues compared with normal tissues (P = 3.5e-07) (Figure 12A). These findings were consistent at the protein level as well, as demonstrated in the FUSCC cohort (P = 0.0037) (Figure 12D). Furthermore, when comparing the expression levels of CYP51A1 among normal tissues, primary RCC (pRCC), and metastatic RCC (mRCC) samples, we observed that CYP51A1 exhibited the highest expression in normal tissues, followed by pRCC and mRCC (Figure 12B). Of note, within tumor samples, the expression level of CYP51A1 in mRCC was relatively diminished compared to pRCC (GSE105261: P=0.0016; GSE73121: P=7.9e-10) (Figure 12B, C). The meticulous analysis of CYP51A1 expression modulation in 786O cells following transfection with siRNA or overexpression plasmids underscores its significant impact on cellular behavior. The evident decrease in both CYP51A1 mRNA and protein levels post-siRNA transfection establishes a clear relationship between CYP51A1 knockdown and reduced gene expression. Conversely, the marked upregulation of CYP51A1 mRNA and protein in the overexpression-transfected group highlights the effectiveness of this technique in altering the cellular abundance of CYP51A1 (Figure 12E, F). The functional implications of these alterations were further explored through a CCK-8 assay, revealing a substantial increase in cell proliferation upon CYP51A1 downregulation and a contrasting decrease in cell proliferation upon CYP51A1 overexpression when compared to control group cells (P < 0.0001) (Figure 12G, H). These findings vividly demonstrate the pivotal role of CYP51A1 in modulating cell proliferation, emphasizing its potential as a regulator of cellular behavior in RCC.

**Figure 13:**
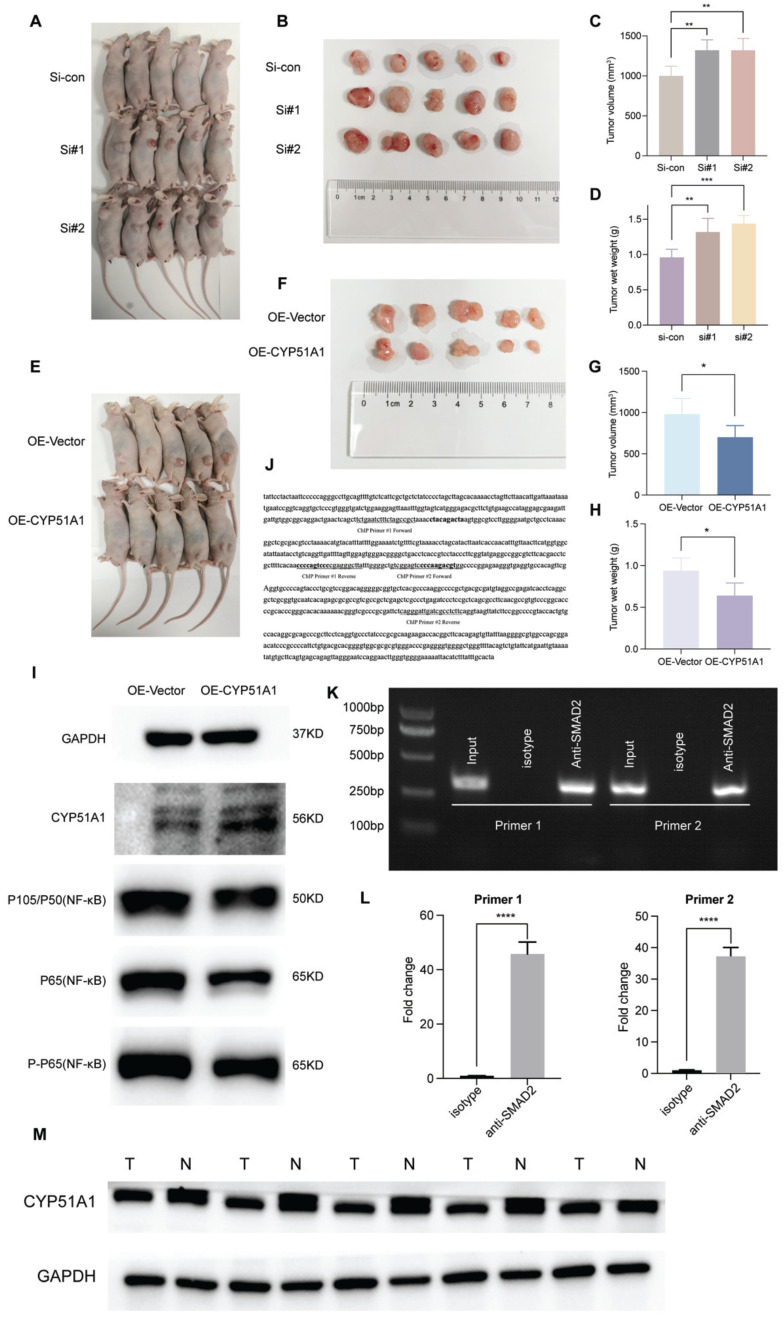
The regulatory mechanisms upstream and downstream of CYP51A1. (A-H) Significant difference in volumes and wet weights of tumor xenografts under different groups. (I) Western blotting analysis of NF-κB signaling pathway of CYP51A1. (J) The binding site was predicted in bold sequences using matinspector software (Genomatix, Munich, Germany). (K) The binding site was amplified by the same specific primers using PCR in the ChIP assay. (L) The quantitative data of CHIP assay. (M) Western blotting analysis of CYP51A1 in 5 pairs of renal cancer tumor samples and adjacent normal samples. The data are analyzed by Student's t-test (two-tailed) and presented as mean ± SD. **P < 0.01, ***P < 0.001, and ****P < 0.0001.

When compared with the control group, tumors in the si-CYP51A1 group grew significantly faster (P < 0.01) and their tumor wet weights increased significantly (P < 0.01) (Figure 13A-D). Additionally, in comparison to the OE-vector group, both tumor volume (P<0.05) and weight (P<0.05) were notably reduced in the OE-CYP51A1 group (Figure 13E-H). Moreover, transwell assays exhibited a significant increase in the migration capacity of 786O cell lines upon CYP51A1 depletion, whereas CYP51A1 overexpression led to decreased migration ability (Figure 12I). Further supporting these findings, the wound healing assay indicated significantly reduced scratch closure in the CYP51A1-overexpression-transfected group compared to the normal control group after 24 hours (P < 0.0001) (Figure 12J). These comprehensive results emphasize the multi-faceted influence of CYP51A1 on RCC, portraying its involvement in tumor growth, migration, and wound healing dynamics. CHIP assays were conducted to determine whether SMAD2 binds to the same region of the CYP51A1 promoter as CYP51A1, concluding that SMAD2 may serve as TF to regulate CYP51A1. Figure 13J-L shows a remarkable enrichment of this promoter fragment with specific antibodies against SMAD2, but not isotype IgG (PCR amplification with primers shown here) (Figure 13J). In addition, we also detected changes in signaling pathways downstream (NF-κB signaling pathway) of CYP51A1. Compared with the OE-Vector group, the protein expression of P105/P50, P65 and P-P65 was decreased in the OE-CYP51A1 group. Additionally, this elucidates that CYP51A1 can suppress tumor proliferation by modulating the NF-KB pathway (Figure 13I).

To elucidate the role of CYP51A1 in immune escape within the tumor microenvironment, we conducted multiplex immunohistochemistry on RCC, RCC-TT, and para-tumor tissues to assess CYP51A1 expression across various cell types in these tissues. Our observations revealed significantly lower CYP51A1 expression in both RCC and RCC-TT tissues compared to adjacent tissues. Interestingly, no substantial difference was noted in CYP51A1 expression between RCC and RCC-TT tissues (Figure 12K, L). In contrast, quantification of immunofluorescence signals demonstrated that RCC-TT tissues exhibited the highest infiltration of CD4/CD8+ T cells, while adjacent tissues displayed the lowest CD4/CD8+ T cell infiltration (Figure 12M, N). Moreover, Figure 12O illustrates a strong correlation between CYP51A1 expression and CD8. Notably, as depicted in Figure 12K, CYP51A1 was co-expressed with both CD4 and CD8 molecules, suggesting a close association between CYP51A1 expression and the infiltration of CD4+ and CD8+ T cells. These findings collectively underscore the pivotal role of CYP51A1 as a mediator in fostering immune activation within the RCC microenvironment.

## Discussion

Over the last decades, compelling epidemiological evidence has unequivocally demonstrated a relentless upswing, approximately 2% per annum, in the incidence and mortality rates of renal cancer worldwide 1,75,76. In addition, it is well-established that tumor patients frequently encounter a constellation of aberrant coagulation complications such as hyper-thrombotic activity or coagulation disorders, with the relationship between VTE and cancer long recognized 77. Notably, studies consistently report that the risk of VTE development in cancer patients is approximately 4- to 7-fold higher than in individuals without cancer 78-80. Further investigations have revealed that within the subset of renal cell carcinoma (RCC) patients exhibiting a hypercoagulable state, approximately 15% manifest the invasion of tumor thrombus (TT) into the inferior vena cava (IVC), with a minute proportion progressing to the extent of TT extension into the right cardiac chambers 5,81. Remarkably, among these cases, nearly 44% demonstrate vein thrombus extension, while the fortuitous occurrence of thrombus extension into the right atrium is observed in only 1-4% of cases 82,83. Significantly, the degree of TT invasion serves as a pivotal determinant of both the stage classification and the subsequent prognosis of cancer. Specifically, the Mayo clinical classification categorizes TT extension into four distinct levels: level I TT, which involves extension into the renal vein and its branches (stage pT3a); level II TT, characterized by invasion into the IVC up to the hepatic veins (pT3b); level III TT, approximating the diaphragm (pT3b); and level IV TT, representing migration into the heart (pT3c) 84. However, to the best of our knowledge, despite these developments, the current clinical approach for treatment decision-making and the formulation of surveillance strategies in RCC predominantly relies on the well-established American Joint Committee on Cancer (AJCC) TNM staging system. It is worth noting that although the Mayo clinical classification of TT extension can be applied to RCC patients with TT, certain limitations persist in accurately predicting clinical outcomes within the same stage owing to the inherenteterogeneity of the disease.

In the era of precision medicine, the treatment landscape for RCC has undergone a remarkable transformation with the advent of molecular targeted therapies and immunotherapies. The existing staging system and Mayo clinical classification of TT fall short of meeting the pressing need for clinicians to accurately assess prognosis and predict treatment response in RCC patients with TT. Consequently, there is an urgent demand for an improved and personalized assessment framework that can be readily implemented in clinical practice, enabling the identification of patients at high risk of refractory disease and poor survival outcomes. While several transcriptional signatures associated with tumor metastasis and thrombosis have been designed in the past 85-87, there remains a dearth of comprehensive bioinformatic and machine-learning analyses aimed at enhancing accuracy and applicability across a broad patient population. To bridge this critical research gap, our study endeavors to elucidate the intricate relationship between TTCRGs and both prognosis and therapeutic benefits, thus paving the way for more precise and effective management strategies in RCC patients with TT.

In this study, to obtain the pathways of RCC-TT and coagulation as entirely as possible, two data sources, the global landscape data of somatic alterations of the Chinese RCC-TT population in Ma's research combined with coagulation-related pathways (hsa04610: Complement and coagulation cascades and hsa04610: Platelet activation) retrieved from the KEGG pathway database, were applied to define TTCRGs. Interestingly, we found that TTCRGs harbored high frequencies of copy number alterations with a poor prognosis. Notably, these copy number alterations were also responsible for the expression imbalance of TTCRGs. Specifically, genes such as LAMN2, F12, and BTNL8 were found to be highly amplified rather than deeply deleted, and their overexpression was closely linked to worse prognosis, suggesting their potential as driver genes in RCC development. By employing the CNMF clustering method, we stratified RCC patients into two distinct clusters. Further analysis of associated clinical characteristics revealed that cluster 2, characterized by a poor survival rate, consisted of more advanced and malignant RCC cases compared to cluster 1. Consistent with these findings, GSEA and GSVA analyses demonstrated that cluster 2 was significantly enriched in immune signaling pathways, including the T cell receptor signaling pathway, B cell receptor signaling pathway, primary immunodeficiency, and immunogenic receptors. These results were in line with the conclusions drawn from different clustering algorithms applied to cluster 2, further supporting the association of this cluster with immune-related processes in RCC-TT.

Here, to account for the impact of venous thrombus on RCC-TT, the WGCNA algorithm was applied to identify TTCRGs. Subsequently, under the LOOCV framework, 10 popular feature extraction methods 101 kinds of combinations, which had high recognition accuracy, could be carried out to construct a TTCRRS signature using multiple well-established public RCC patients' cohorts. Notably, among these algorithms, LASSO, RSF, CoxBoost, and StepCox played a pivotal role in reducing dimensionality and screening variables. By employing a random combination mode, we were able to simplify and streamline the final model, enhancing its translational potential. Ultimately, the combination of LASSO and GBM, which had the largest average C-index (0.631) in the validation cohorts, was defined as the optimal model. Kaplan Meier analysis, ROC curve and C-index all exhibited our TTCRRS had high accuracy and stable performance in several independent cohorts, which had the potential to greatly facilitate medical practice. Additionally, we expanded our investigation by collecting and analyzing 129 published RCC signatures, encompassing numerous combinations of functional genes. However, it is important to note that most of these signatures have not been thoroughly validated nor applied in clinical settings for evaluating the prognosis of RCC-TT patients 88-90. Of note, most signatures performed better on their own training cohorts and a few external cohorts but performed weakly on other new cohorts 91,92, which might be due to awful generalization ability from overfitting. Interestingly, the results of our univariate Cox regression analysis characterized that only our TTCRRS presents excellent prognostic significance in almost all cohorts, indicating it has a superior extrapolation possibility. Although our signature is not as accurate as the TNM staging system and pathological grade, the TTCRRS could be a supplementary tool to generalize for clinical applications. Nevertheless, it is important to acknowledge that the stability and robustness of the TTCRRS signature in the proteome were relatively weaker compared to its performance in the transcriptome, delineating that the TTCRRS signature is most likely applicable to transcriptomics.

Cancer immunotherapy has brought about a paradigm shift in the treatment of solid tumors, revolutionizing traditional approaches 63,93. Next, we further explored the immune landscape between high and low TTCRRS groups. Compared with the low-TTCRRS group, the high-TTCRRS group had a superior abundance of immune cell infiltrations, such as activated CD4^+^ T cell, activated CD8^+^ T cell, memory CD4^+^ T cell, memory CD8^+^ T cell, and these effector cells might enhance the anti-tumor immunity, bring better immunotherapy effectiveness. And, more importantly, we found the expression of some signatures in immune pathway activation were dramatically elevated in patients with high TTCRRS. The field of precision medicine emphasizes the need for early identification of patients who are responsive to different treatment regimens, tailored to their individual needs. Thus, we also selected some chemotherapeutic and targeted drugs between high and low TTCRRS group patients. To sum up, these findings implied that our TTCRRS signatures hold the potential to guide the identification of therapy-sensitive RCC-TT patients who may benefit from first-line immunotherapy, as well as chemotherapeutic and targeted therapies. This has significant implications for personalized medicine and underscores the importance of leveraging the predictive power of the TTCRRS signatures in clinical decision-making.

To enable clinicians to predict the prognosis of RCC-TT patients based on the quantification of the TTCRRS signature, we developed a nomogram that incorporated both the TTCRRS and subtype information. Through rigorous evaluation using ROC curve analysis, calibration curves, and decision curves, the nomogram demonstrated excellent predictive ability. Afterward, we used 10 machine-learning approaches to select the optimal feature subset from our TTCRRS signatures. Then, to gain further insights into the underlying regulatory mechanisms, we analyzed the TFs possibly binding to the promoter region of the top features (CYP51A1) selected by the machine-learning method with Cistrome DB Kit, screening out several TFs (BRD4, SMAD2, CHD7, POLR2A, TRRAP), which was positively correlated with the expression of CYP51A1. Ultimately, the single-cell database CancerSEA was leveraged to predict the functional implications of the most important feature. We found a positive correlation between the high expresson of CYP51A1 with hypoxia 94.

Functioning as a vital member of the cytochrome P450 superfamily of enzymes primarily localized in the endoplasmic reticulum of diverse tissues, including the liver and adrenal glands, CYP51A1, also known as lanosterol 14-alpha demethylase, plays a pivotal role in the intricate biosynthesis of cholesterol 95. In addition, according to the druggable genome from Ensembl v.73 redefined by Finan et al's criterion, CYP51A1 is a Tier 1 target protein (This tier encompasses the targets of approved drugs as well as drugs in various stages of clinical development) 96 (Table S9). They are also the target protein of small molecule and biotherapeutic drugs identified using manually curated efficacy target information from release 17 of the ChEMBL database 97. However, to the best of our knowledge, the association between CYP51A1 and RCC remains a subject that has not received extensive scrutiny and comprehension. There is an indication that the regulation of CYP51A1 function may bear considerable implications in the therapeutic strategies for oncological pathologies 98. The accumulation of cholesterol represents a pervasive hallmark within cancerous tissue, and emerging research 99 has unequivocally elucidated its pivotal involvement in the pathogenesis of various malignancies, including breast, bladder, and colorectal cancer, among others. In addition, kidney cancer cells, as well as other tumor cells, exhibit an increased demand for elevated cholesterol levels to support essential processes such as cell membrane biogenesis and other functional requisites compared to normal cells. Based on our experimental findings, we have conjectured that the downregulation of CYP51A1 in renal cancer tissues triggers an escalation in endogenous cholesterol biosynthesis through the NF-κB signaling pathway, thereby instigating tumor progression.

## Conclusions

In conclusion, by utilizing advanced bioinformatics techniques and innovative machine-learning methods, we have successfully developed a robust and versatile signature based on the expression of 10 TTCRGs. This signature holds immense potential in evaluating the prognosis and therapeutic benefits for RCC-TT patients. By providing valuable insights into the molecular characteristics of RCC-TT, the TTCRRS signature serves as a valuable supplementary tool to optimize the clinical decision-making process and guide individualized treatment approaches. Specifically, it can aid in optimizing adjuvant chemotherapy and anticancer immunotherapy strategies for RCC-TT patients, ultimately leading to improved treatment outcomes and personalized care.

## Supplementary Material

Supplementary figures.

Supplementary tables.

## Figures and Tables

**Figure 4:**
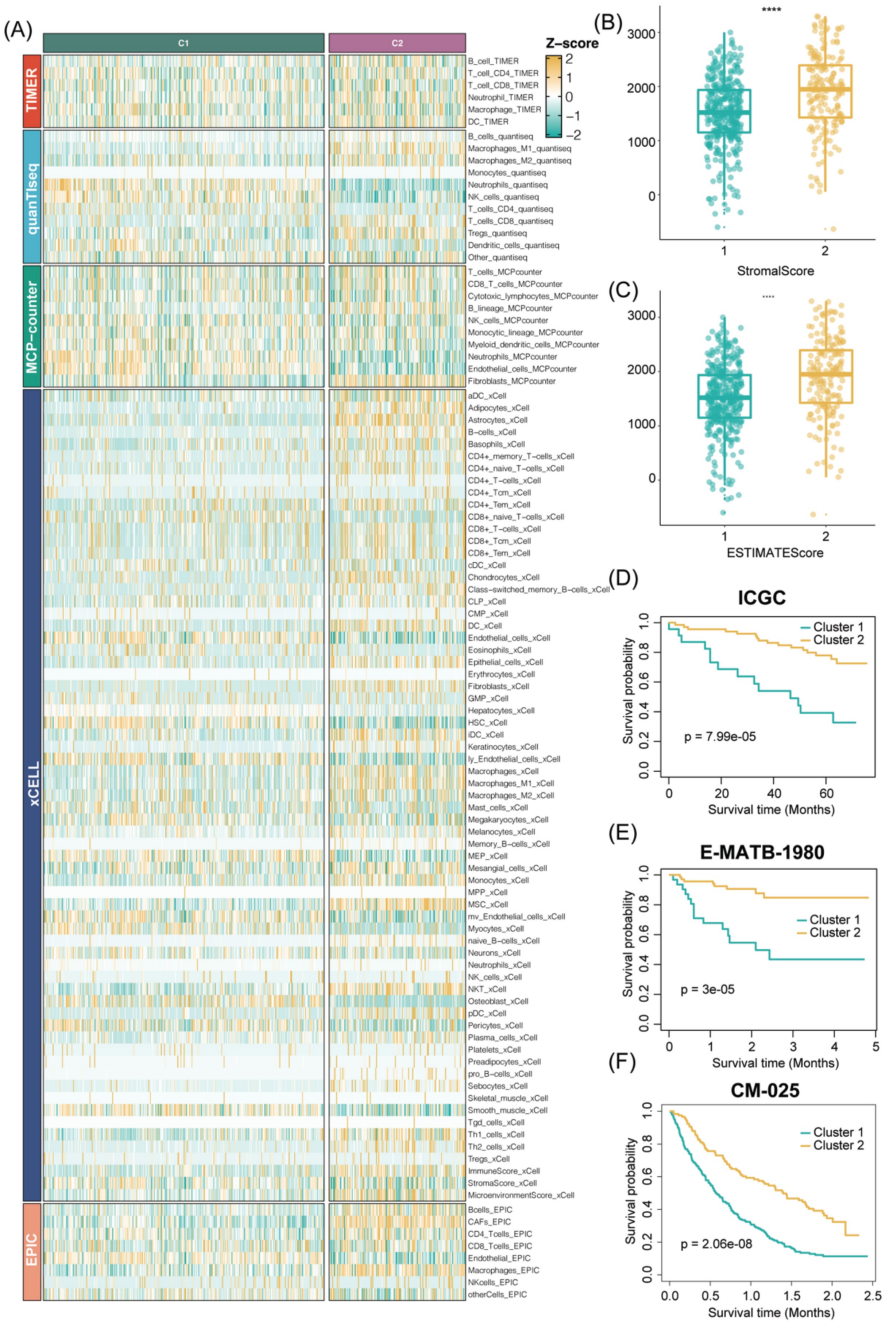
Validation of our TTC clusters criteria. (|A) Five other algorithms (TIMER, quanTIseq, MCP-counter, xCell and EPIC) further verified the robustness and stability of our ssGSEA results. (B-C) The box plot reveals the distribution of stromal scores and ESTIMATE scores inferred by the ESTIMATE algorithm between two TTC clusters. (D-F) Kaplan-Meier curve for OS between two TTC clusters in the other 3 independent cohorts (ICGC, E-MTAB-1980, CM-025). **** means P < 0.0001.

## Abbreviations

RCC: Renal cell carcinoma
TNM: Tumor Node Metastasis
TTCRRS: tumor thrombus coagulation-related risk stratification
CRGs: coagulation-related genes
VTE: venous thromboembolism
tumor thrombus: TT
DSS: disease-specific survival
RCC-TT: RCC patients with TT
TCGA: The Cancer Genome Atlas
GEO: Gene Expression Omnibus
CPTAC: Clinical Proteomic Tumor Analysis Consortium
FUSCC: Fudan University Shanghai Cancer Center
TPM: transcript per kilobase million
RMA: robust multichip average
CNA: copy number variations
CC: Consensus clustering
CNMF: Consensus nonnegative matrix factorization
SNF-CC: Combined similarity network fusion and CC
CDF: cumulative distribution function
ssGSEA: single sample gene set enrichment analysis
NES: normalized enrichment score
GSVA: gene set variation analysis
DEGs: differentially expressed genes
GSEA: gene set enrichment analysis
TME: tumor microenvironment
MeTIL: methylation of tumor-infiltrating lymphocyte
IC50: semi-inhibitory concentration
WGCNA: Weight correlation network analysis
TOM: topological overlap matrix
LASSO: Least absolute shrinkage and selection operator
GBM: generalized boosted regression modeling
RSF: random survival forest
Enet: elastic network
plsRcox: partial least squares regression for Cox
SuperPC: supervised principal components
survival-SVM: survival support vector machine
LOOCV: leave-one-out cross-validation
C-index: concordance index
PD: progressive disease
SD: stable disease
CR: complete response
PR: partial response
R: responder
NR: non-responder
time-ROC: Time-dependent receiver operating characteristic
DCA: decision curve analysis
CMAP: The Connectivity map
ICB: immune checkpoint blockade
SHAP: Shapley Additive Explanation
TFs: Transcription factors
IVC: inferior vena cava

## References

[1] Sung H, Ferlay J, Siegel RL (2021). Global Cancer Statistics 2020: GLOBOCAN Estimates of Incidence and Mortality Worldwide for 36 Cancers in 185 Countries. CA Cancer J Clin.

[2] Serrano PE, Parpia S, Linkins LA (2018). Venous Thromboembolic Events Following Major Pelvic and Abdominal Surgeries for Cancer: A Prospective Cohort Study. Ann Surg Oncol.

[3] Ciancio G, Soloway M (2005). Resection of the abdominal inferior vena cava for complicated renal cell carcinoma with tumour thrombus. BJU Int.

[4] Zisman A, Wieder JA, Pantuck AJ (2003). Renal cell carcinoma with tumor thrombus extension: biology, role of nephrectomy and response to immunotherapy. J Urol.

[5] Psutka SP, Leibovich BC (2015). Management of inferior vena cava tumor thrombus in locally advanced renal cell carcinoma. Ther Adv Urol.

[6] Reese AC, Whitson JM, Meng MV (2013). Natural history of untreated renal cell carcinoma with venous tumor thrombus. Urol Oncol.

[7] Dvorak HF (2019). Tumors: Wounds That Do Not Heal-A Historical Perspective with a Focus on the Fundamental Roles of Increased Vascular Permeability and Clotting. Semin Thromb Hemost.

[8] Adam F, Kauskot A, Rosa JP (2008). Mitogen-activated protein kinases in hemostasis and thrombosis. J Thromb Haemost.

[9] Evans CE, Grover SP, Saha P (2014). Suppression of angiogenic response in local vein wall is associated with reduced thrombus resolution. Thromb Res.

[10] Govind Babu K, Bhat GR (2016). Cancer-associated thrombotic microangiopathy. Ecancermedicalscience.

[11] Posch F, Thaler J, Zlabinger GJ (2016). Soluble Vascular Endothelial Growth Factor (sVEGF) and the Risk of Venous Thromboembolism in Patients with Cancer: Results from the Vienna Cancer and Thrombosis Study (CATS). Clin Cancer Res.

[12] Saidak Z, Soudet S, Lottin M (2021). A pan-cancer analysis of the human tumor coagulome and its link to the tumor immune microenvironment. Cancer Immunol Immunother.

[13] Nambirajan T, Jeschke S, Al-Zahrani H (2004). Prospective, randomized controlled study: transperitoneal laparoscopic versus retroperitoneoscopic radical nephrectomy. Urology.

[14] Blute ML, Leibovich BC, Lohse CM (2004). The Mayo Clinic experience with surgical management, complications and outcome for patients with renal cell carcinoma and venous tumour thrombus. BJU Int.

[15] Paner GP, Stadler WM, Hansel DE (2018). Updates in the Eighth Edition of the Tumor-Node-Metastasis Staging Classification for Urologic Cancers. Eur Urol.

[16] Shi Y, Zhang Q, Bi H (2022). Decoding the multicellular ecosystem of vena caval tumor thrombus in clear cell renal cell carcinoma by single-cell RNA sequencing. Genome Biol.

[17] Wang XM, Lu Y, Song YM (2020). Integrative genomic study of Chinese clear cell renal cell carcinoma reveals features associated with thrombus. Nat Commun.

[18] Bian Z, Fan R, Xie L (2022). A Novel Cuproptosis-Related Prognostic Gene Signature and Validation of Differential Expression in Clear Cell Renal Cell Carcinoma. Genes (Basel).

[19] Smith CC, Beckermann KE, Bortone DS (2018). Endogenous retroviral signatures predict immunotherapy response in clear cell renal cell carcinoma. J Clin Invest.

[20] Wistuba-Hamprecht K, Gouttefangeas C, Weide B (2020). Immune Signatures and Survival of Patients With Metastatic Melanoma, Renal Cancer, and Breast Cancer. Front Immunol.

[21] Necchi A, Joseph RW, Loriot Y (2017). Atezolizumab in platinum-treated locally advanced or metastatic urothelial carcinoma: post-progression outcomes from the phase II IMvigor210 study. Ann Oncol.

[22] Riaz N, Havel JJ, Makarov V (2017). Tumor and Microenvironment Evolution during Immunotherapy with Nivolumab. Cell.

[23] Irizarry RA, Bolstad BM, Collin F (2003). Summaries of Affymetrix GeneChip probe level data. Nucleic Acids Res.

[24] Johnson WE, Li C, Rabinovic A (2007). Adjusting batch effects in microarray expression data using empirical Bayes methods. Biostatistics.

[25] Leek JT, Johnson WE, Parker HS (2012). The sva package for removing batch effects and other unwanted variation in high-throughput experiments. Bioinformatics.

[26] Qu Y, Feng J, Wu X (2022). A proteogenomic analysis of clear cell renal cell carcinoma in a Chinese population. Nat Commun.

[27] Gao J, Aksoy BA, Dogrusoz U (2013). Integrative analysis of complex cancer genomics and clinical profiles using the cBioPortal. Sci Signal.

[28] Mayakonda A, Lin DC, Assenov Y (2018). Maftools: efficient and comprehensive analysis of somatic variants in cancer. Genome Res.

[29] Coleman S, Kirk PDW, Wallace C (2022). Consensus clustering for Bayesian mixture models. BMC Bioinformatics.

[30] Brunet JP, Tamayo P, Golub TR (2004). Metagenes and molecular pattern discovery using matrix factorization. Proc Natl Acad Sci U S A.

[31] Xu T, Le TD, Liu L (2017). CancerSubtypes: an R/Bioconductor package for molecular cancer subtype identification, validation and visualization. Bioinformatics.

[32] Hänzelmann S, Castelo R, Guinney J (2013). GSVA: gene set variation analysis for microarray and RNA-seq data. BMC Bioinformatics.

[33] Charoentong P, Finotello F, Angelova M (2017). Pan-cancer Immunogenomic Analyses Reveal Genotype-Immunophenotype Relationships and Predictors of Response to Checkpoint Blockade. Cell Rep.

[34] Li T, Fan J, Wang B (2017). TIMER: A Web Server for Comprehensive Analysis of Tumor-Infiltrating Immune Cells. Cancer Res.

[35] Plattner C, Finotello F, Rieder D (2020). Deconvoluting tumor-infiltrating immune cells from RNA-seq data using quanTIseq. Methods Enzymol.

[36] Becht E, Giraldo NA, Lacroix L (2016). Estimating the population abundance of tissue-infiltrating immune and stromal cell populations using gene expression. Genome Biol.

[37] Aran D, Hu Z, Butte AJ (2017). xCell: digitally portraying the tissue cellular heterogeneity landscape. Genome Biol.

[38] Racle J, Gfeller D (2020). EPIC: A Tool to Estimate the Proportions of Different Cell Types from Bulk Gene Expression Data. Methods Mol Biol.

[39] Yoshihara K, Shahmoradgoli M, Martínez E (2013). Inferring tumour purity and stromal and immune cell admixture from expression data. Nat Commun.

[40] Ritchie ME, Phipson B, Wu D (2015). limma powers differential expression analyses for RNA-sequencing and microarray studies. Nucleic Acids Res.

[41] Yu G, Wang LG, Han Y (2012). clusterProfiler: an R package for comparing biological themes among gene clusters. OMICS.

[42] Nakazato T, Bono H, Matsuda H (2009). Gendoo: functional profiling of gene and disease features using MeSH vocabulary. Nucleic Acids Res.

[43] Maglott D, Ostell J, Pruitt KD (2011). Entrez Gene: gene-centered information at NCBI. Nucleic Acids Res.

[44] Jeschke J, Bizet M, Desmedt C (2017). DNA methylation-based immune response signature improves patient diagnosis in multiple cancers. J Clin Invest.

[45] Geeleher P, Cox N, Huang RS (2014). pRRophetic: an R package for prediction of clinical chemotherapeutic response from tumor gene expression levels. PLoS One.

[46] Geeleher P, Cox NJ, Huang RS (2014). Clinical drug response can be predicted using baseline gene expression levels and *in vitro* drug sensitivity in cell lines. Genome Biol.

[47] Langfelder P, Horvath S (2008). WGCNA: an R package for weighted correlation network analysis. BMC Bioinformatics.

[48] Liu Z, Liu L, Weng S (2022). Machine learning-based integration develops an immune-derived lncRNA signature for improving outcomes in colorectal cancer. Nat Commun.

[49] Xu D, Wang Y, Liu X (2021). Development and clinical validation of a novel 9-gene prognostic model based on multi-omics in pancreatic adenocarcinoma. Pharmacol Res.

[50] Blanche P, Dartigues JF, Jacqmin-Gadda H (2013). Estimating and comparing time-dependent areas under receiver operating characteristic curves for censored event times with competing risks. Stat Med.

[51] Schröder MS, Culhane AC, Quackenbush J (2011). survcomp: an R/Bioconductor package for performance assessment and comparison of survival models. Bioinformatics.

[52] Kerr KF, Brown MD, Zhu K (2016). Assessing the Clinical Impact of Risk Prediction Models With Decision Curves: Guidance for Correct Interpretation and Appropriate Use. J Clin Oncol.

[53] Lamb J, Crawford ED, Peck D (2006). The Connectivity Map: using gene-expression signatures to connect small molecules, genes, and disease. Science.

[54] Binnewies M, Roberts EW, Kersten K, Chan V, Fearon DF, Merad M, Coussens LM, Gabrilovich DI, Ostrand-Rosenberg S, Hedrick CC (2018). Understanding the tumor immune microenvironment (TIME) for effective therapy. Nat Med.

[55] Thorsson V, Gibbs DL, Brown SD (2018). The Immune Landscape of Cancer. Immunity.

[56] Siska PJ, Beckermann KE, Mason FM (2017). Mitochondrial dysregulation and glycolytic insufficiency functionally impair CD8 T cells infiltrating human renal cell carcinoma. JCI Insight.

[57] Giraldo NA, Becht E, Vano Y (2017). Tumor-Infiltrating and Peripheral Blood T-cell Immunophenotypes Predict Early Relapse in Localized Clear Cell Renal Cell Carcinoma. Clin Cancer Res.

[58] Chevrier S, Levine JH, Zanotelli VRT (2017). An Immune Atlas of Clear Cell Renal Cell Carcinoma. Cell.

[59] Lemoine R, Velge-Roussel F, Herr F (2010). Interferon gamma licensing of human dendritic cells in T-helper-independent CD8+ alloimmunity. Blood.

[60] Aoun F, Rassy EE, Assi T (2017). Advances in urothelial bladder cancer immunotherapy, dawn of a new age of treatment. Immunotherapy.

[61] Hoos A (2016). Development of immuno-oncology drugs - from CTLA4 to PD1 to the next generations. Nat Rev Drug Discov.

[62] Couzin-Frankel J (2013). Breakthrough of the year 2013. Cancer immunotherapy. Science.

[63] Kamal Y, Cheng C, Frost HR (2018). Predictors of disease aggressiveness influence outcome from immunotherapy treatment in renal clear cell carcinoma. Oncoimmunology.

[64] Böttcher JP, Bonavita E, Chakravarty P (2018). NK Cells Stimulate Recruitment of cDC1 into the Tumor Microenvironment Promoting Cancer Immune Control. Cell.

[65] Chauvin JM, Zarour HM (2020). TIGIT in cancer immunotherapy. J Immunother Cancer.

[66] Fuertes MB, Kacha AK, Kline J (2011). Host type I IFN signals are required for antitumor CD8+ T cell responses through CD8{alpha}+ dendritic cells. J Exp Med.

[67] Rotte A, Sahasranaman S, Budha N (2021). Targeting TIGIT for Immunotherapy of Cancer: Update on Clinical Development. Biomedicines.

[68] Ruffo E, Wu RC, Bruno TC (2019). Lymphocyte-activation gene 3 (LAG3): The next immune checkpoint receptor. Semin Immunol.

[69] Zheng L, Qin S, Si W (2021). Pan-cancer single-cell landscape of tumor-infiltrating T cells. Science.

[70] Bhattacharya S, Andorf S, Gomes L (2014). ImmPort: disseminating data to the public for the future of immunology. Immunol Res.

[71] Bindea G, Mlecnik B, Tosolini M (2013). Spatiotemporal dynamics of intratumoral immune cells reveal the immune landscape in human cancer. Immunity.

[72] Miao YR, Zhang Q, Lei Q (2020). ImmuCellAI: A Unique Method for Comprehensive T-Cell Subsets Abundance Prediction and its Application in Cancer Immunotherapy. Adv Sci (Weinh).

[73] Newman AM, Liu CL, Green MR (2015). Robust enumeration of cell subsets from tissue expression profiles. Nat Methods.

[74] Nirmal AJ, Regan T, Shih BB (2018). Immune Cell Gene Signatures for Profiling the Microenvironment of Solid Tumors. Cancer Immunol Res.

[75] Capitanio U, Bensalah K, Bex A (2019). Epidemiology of Renal Cell Carcinoma. Eur Urol.

[76] Ferlay J, Colombet M, Soerjomataram I (2018). Cancer incidence and mortality patterns in Europe: Estimates for 40 countries and 25 major cancers in 2018. Eur J Cancer.

[77] Clark K (1998). The risk of a diagnosis of cancer after primary deep venous thrombosis or pulmonary embolism. J Insur Med.

[78] Chew HK, Wun T, Harvey D (2006). Incidence of venous thromboembolism and its effect on survival among patients with common cancers. Arch Intern Med.

[79] Kok VC (2017). Bidirectional risk between venous thromboembolism and cancer in East Asian patients: synthesis of evidence from recent population-based epidemiological studies. Cancer Manag Res.

[80] Timp JF, Braekkan SK, Versteeg HH (2013). Epidemiology of cancer-associated venous thrombosis. Blood.

[81] Noguchi K, Hori D, Nomura Y (2012). Renal cell carcinoma with tumor-thrombus extension into the right ventricle. Ann Vasc Dis.

[82] Ciancio G, Manoharan M, Katkoori D (2010). Long-term survival in patients undergoing radical nephrectomy and inferior vena cava thrombectomy: single-center experience. Eur Urol.

[83] Laguna MP, Algaba F, Cadeddu J (2014). Clinical Research Office of the Endourological Society Renal Mass Study. Current patterns of presentation and treatment of renal masses: a clinical research office of the endourological society prospective study. J Endourol.

[84] Martínez-Salamanca JI, Huang WC, Millán I (2011). International Renal Cell Carcinoma-Venous Thrombus Consortium. Prognostic impact of the 2009 UICC/AJCC TNM staging system for renal cell carcinoma with venous extension. Eur Urol.

[85] Hogg PJ, Owensby DA, Mosher DF (1993). Thrombospondin is a tight-binding competitive inhibitor of neutrophil elastase. J Biol Chem.

[86] Sol N, Wurdinger T (2017). Platelet RNA signatures for the detection of cancer. Cancer Metastasis Rev.

[87] Warsow G, Hübschmann D, Kleinheinz K (2018). Genomic features of renal cell carcinoma with venous tumor thrombus. Sci Rep.

[88] Peng Q, Shen Y, Fu K (2021). Artificial intelligence prediction model for overall survival of clear cell renal cell carcinoma based on a 21-gene molecular prognostic score system. Aging (Albany NY).

[89] Tao Z, Zhang E, Li L (2021). A united risk model of 11 immune-related gene pairs and clinical stage for prediction of overall survival in clear cell renal cell carcinoma patients. Bioengineered.

[90] Wu Y, Wei X, Feng H (2020). An eleven metabolic gene signature-based prognostic model for clear cell renal cell carcinoma. Aging (Albany NY).

[91] Chen H, Xie J, Jin P (2020). Assessment of hazard immune-related genes and tumor immune infiltrations in renal cell carcinoma. Am J Transl Res.

[92] Yuan B, Li F, Li Y (2020). Construction of a 13-Gene Signature as a Novel Prognostic Marker for Patients with Clear Cell Renal Cell Carcinoma and the Role of XCR1 in Cell Proliferation. Cancer Manag Res.

[93] Tang J, Shalabi A, Hubbard-Lucey VM (2018). Comprehensive analysis of the clinical immuno-oncology landscape. Ann Oncol.

[94] Gupta N, Zhao YY, Evans CE (2019). The stimulation of thrombosis by hypoxia. Thromb Res.

[95] Kaluzhskiy L, Ershov P, Yablokov E (2021). Human Lanosterol 14-Alpha Demethylase (CYP51A1) Is a Putative Target for Natural Flavonoid Luteolin 7,3'-Disulfate. Molecules.

[96] Finan C, Gaulton A, Kruger FA (2017). The druggable genome and support for target identification and validation in drug development. Sci Transl Med.

[97] Gaulton A, Bellis LJ, Bento AP (2012). ChEMBL: a large-scale bioactivity database for drug discovery. Nucleic Acids Res.

[98] Martínez-Botas J, Ferruelo AJ, Suárez Y (1998). Induction of apoptosis in p53-null HL-60 cells by inhibition of lanosterol 14-alpha demethylase. Biochimie.

[99] Hao B, Peng X, Bi B (2019). Preoperative serum high-density lipoprotein cholesterol as a predictor of poor survival in patients with clear cell renal cell cancer. Int J Biol Markers.

