# Supplementary material for: Machine Learning-based Framework Develops a Tumor Thrombus Coagulation Signature in Multicenter Cohorts for Renal Cancer
Source: Int J Biol Sci. 2024 Jul 1;20(9):3590–620. doi: 10.7150/ijbs.94555 (PMC11234220; doi:10.7150/ijbs.94555)
Supplement: Supplementary file 1 — Supplementary figures. [file ijbsv20p3590s1.pdf]

Supplemental Figures:

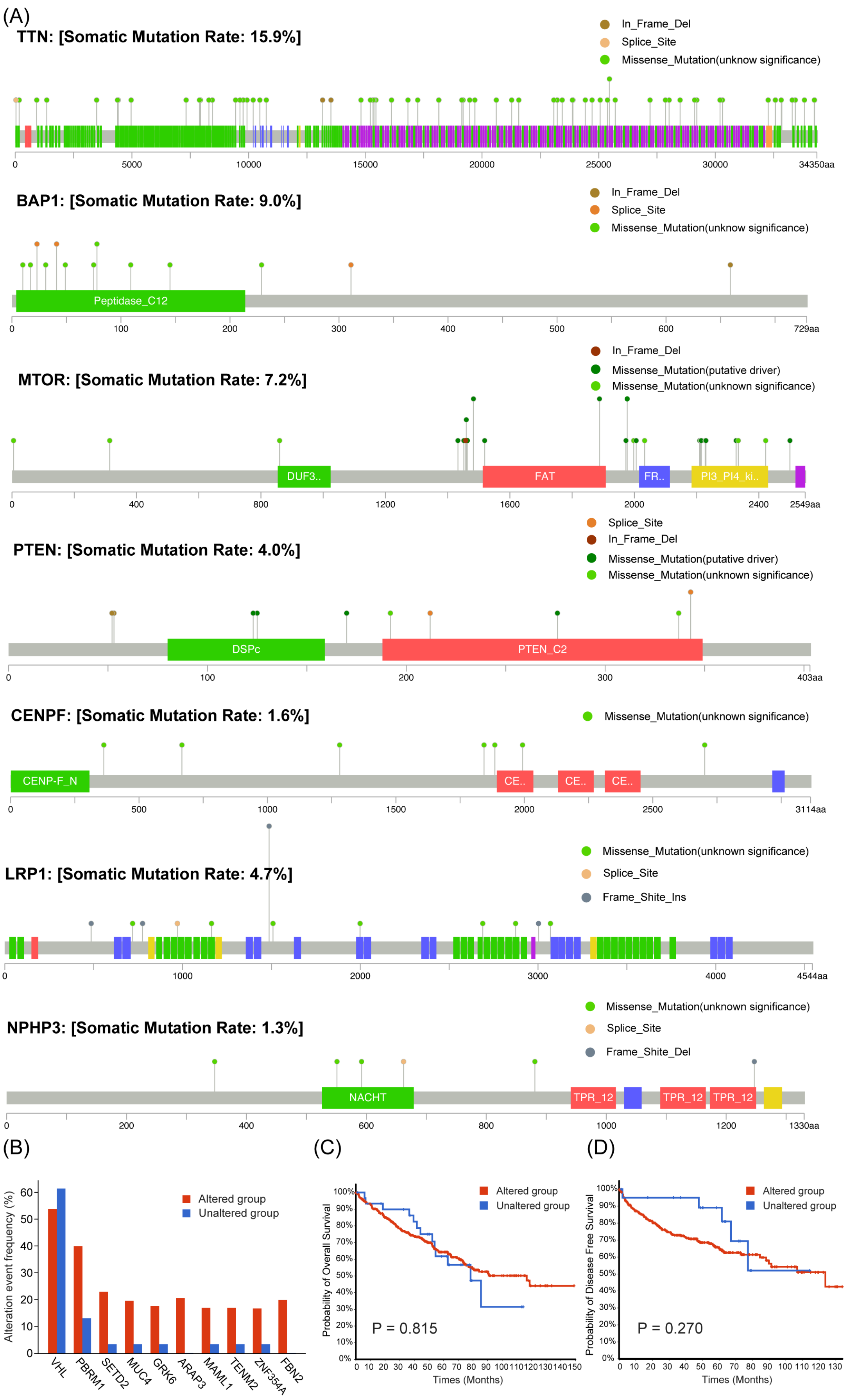

(A)
TTN: [Somatic Mutation Rate: 15.9%]
In_Frame_Del
Splice_Site
Missense_Mutation(unknow significance)
0
5000
10000
15000
20000
25000
30000
34350aa
BAP1: [Somatic Mutation Rate: 9.0%]
In_Frame_Del
Splice_Site
Missense_Mutation(unknow significance)
Peptidase_C12
0
100
200
300
400
500
600
729aa
MTOR: [Somatic Mutation Rate: 7.2%]
In_Frame_Del
Missense_Mutation(putative driver)
Missense_Mutation(unknown significance)
DUF3..
FAT
FR..
PI3_PI4_ki..
0
400
800
1200
1600
2000
2400
2549aa
PTEN: [Somatic Mutation Rate: 4.0%]
Splice_Site
In_Frame_Del
Missense_Mutation(putative driver)
Missense_Mutation(unknown significance)
DSPc
PTEN_C2
0
100
200
300
403aa
CENPF: [Somatic Mutation Rate: 1.6%]
Missense_Mutation(unknown significance)
CENP-F_N
CE..
CE..
CE..
0
500
1000
1500
2000
2500
3114aa
LRP1: [Somatic Mutation Rate: 4.7%]
Missense_Mutation(unknown significance)
Splice_Site
Frame_Shite_Ins
0
1000
2000
3000
4000
4544aa
NPHP3: [Somatic Mutation Rate: 1.3%]
Missense_Mutation(unknown significance)
Splice_Site
Frame_Shite_Del
NACHT
TPR_12
TPR_12
TPR_12
0
200
400
600
800
1000
1200
1330aa
(B)
Alteration event frequency (%)
Altered group
Unaltered group
VHL
PBRM1
SETD2
MUC4
GRK6
ARAP3
MAML1
TENM2
ZNF354A
FBN2
(C)
Probability of Overall Survival
Altered group
Unaltered group
P = 0.815
Times (Months)
(D)
Probability of Disease Free Survival
Altered group
Unaltered group
P = 0.270
Times (Months)

Figure S1 Mutation landscapes of TTCRGs in RCC patients.

(A) Somatic mutations of some top genes in the OncoPrint.

(B) Histogram of the proportion of different mutation groups in RCC.

(C-D) RCC patients' overall survival and relapse-free survival between altered and unaltered mutation groups.

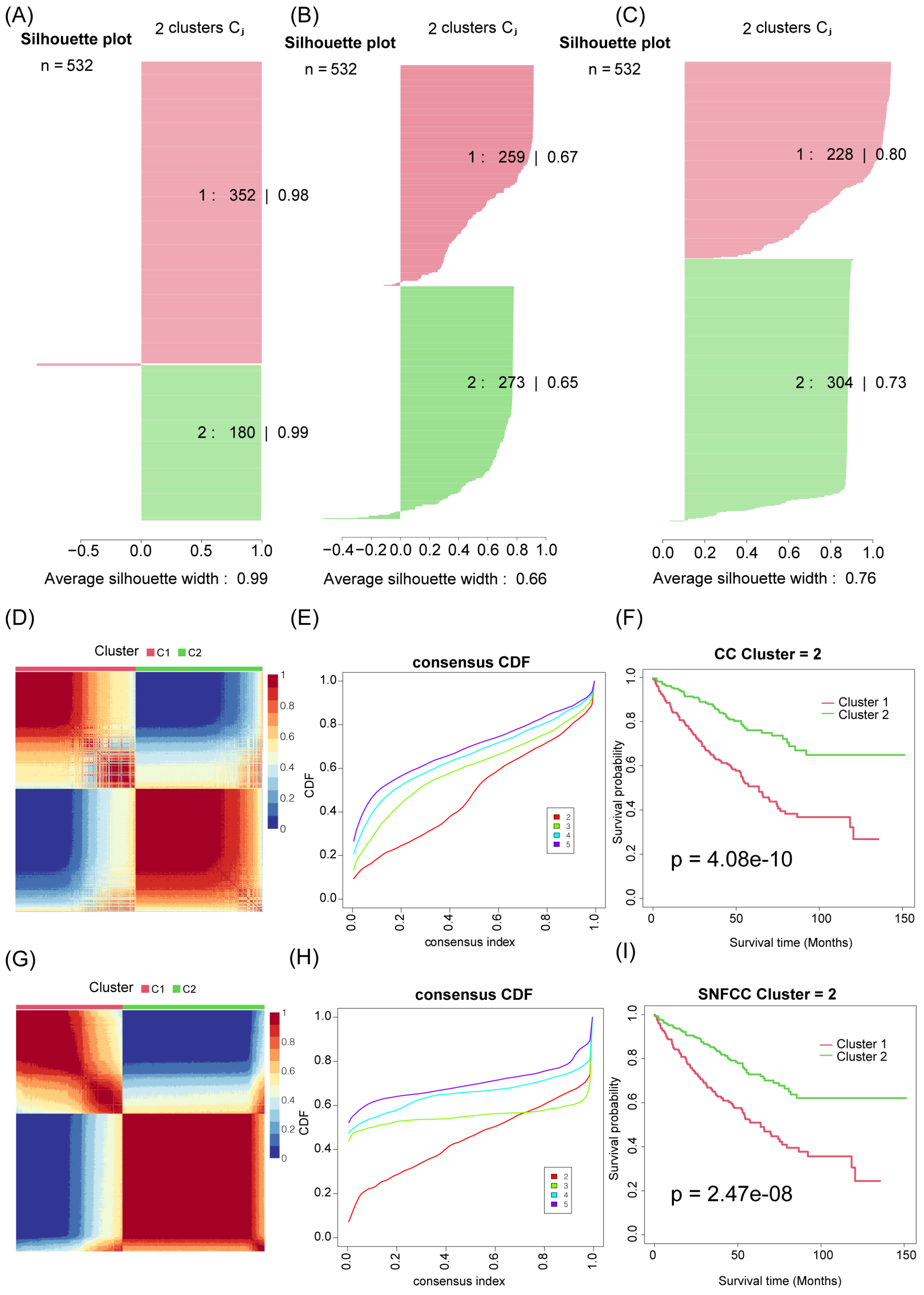

Figure S2 Unsupervised cluster analysis of the RCC patients based on 3 algorithms (CC, SNFCC and CNMF).

(A-C) Average silhouette width plots represent the coherence of clusters. Similar samples in each cluster via 3 algorithms are gathered and a high value of average silhouette width means the correlation between

samples more accurately and felicitously isolates the subgroups.

(D,G) The clustering heatmap visualizes the degree of the partitioning of the sample clusters with CC and SNFCC algorithms, respectively.

(E,H) The CDF curves of the consensus matrix for each k (2-9) using the CC and SNFCC algorithms.

(F,I) Log-rank test p-value for Kaplan–Meier survival analysis between two clusters isolated with CC and SNFCC algorithms.

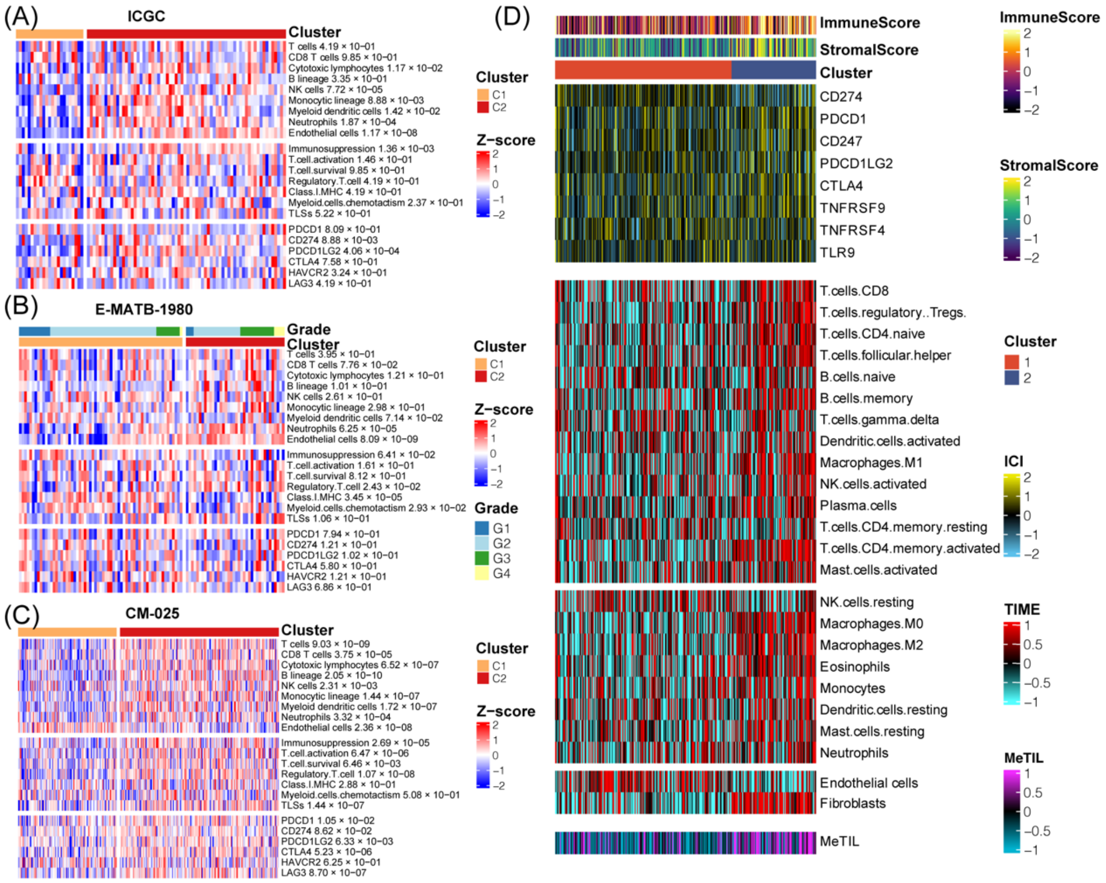

Figure S3 Immune characteristics between two clusters in different independent cohorts.

(A-C) The Correlation between the composition of the TME, expression of gene signatures related to the functional orientation of the immune TME, expression of genes related to immune checkpoints defined by the MCP-counter Z- scores and TTC clusters in the ICGC, E-MTAB-1980, and CM-025 cohorts. Adjusted P values are obtained from Benjamini–Hochberg correction of two-sided Kruskal–Wallis tests P values.

(D) Heatmap shows RCC patients' immune profiles across TTC subtypes. The top panel shows the expression of genes involved in immune checkpoint targets and the bottom panel shows the enrichment

level of 24 microenvironment cell types. The immune enrichment score, stromal enrichment score and DNA methylation of tumor-infiltrating lymphocytes (MeTILs) were annotated at the top of the heatmap.

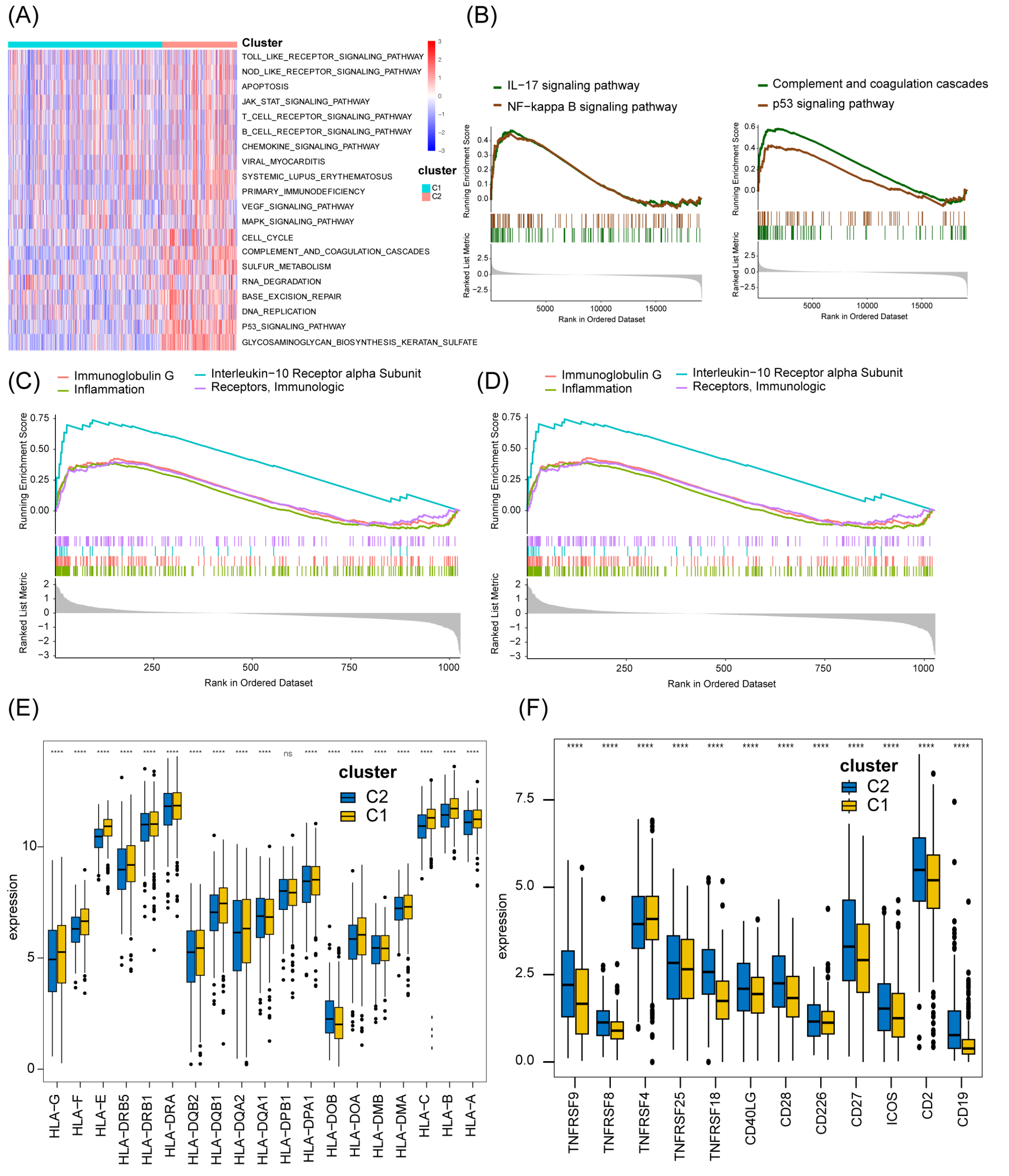

Figure S4 Clinical significance and immune landscape of TTCRRS subtypes in the TCGA cohort.

(A) The biological pathways of two TTC clusters inferred with the GSVA algorithm. Red represents the activation of biological pathways and blue represents the inhibition of biological pathways.

(B) The significant enrichment of biological pathways inferred with the GSEA algorithm. Genes are ranked by logFC of two TTC clusters.

(C-D) GSEA analysis delineated the MeSH terms associated with TTCRRS by using terms of gendoo

and gene2pubmed in the TCGA cohort.

(E-F) Gene expression of HLA and MHC gene sets between two distinct TTC clusters. Statistical significance at the level of ns ≥ 0.05, **** < 0.0001.

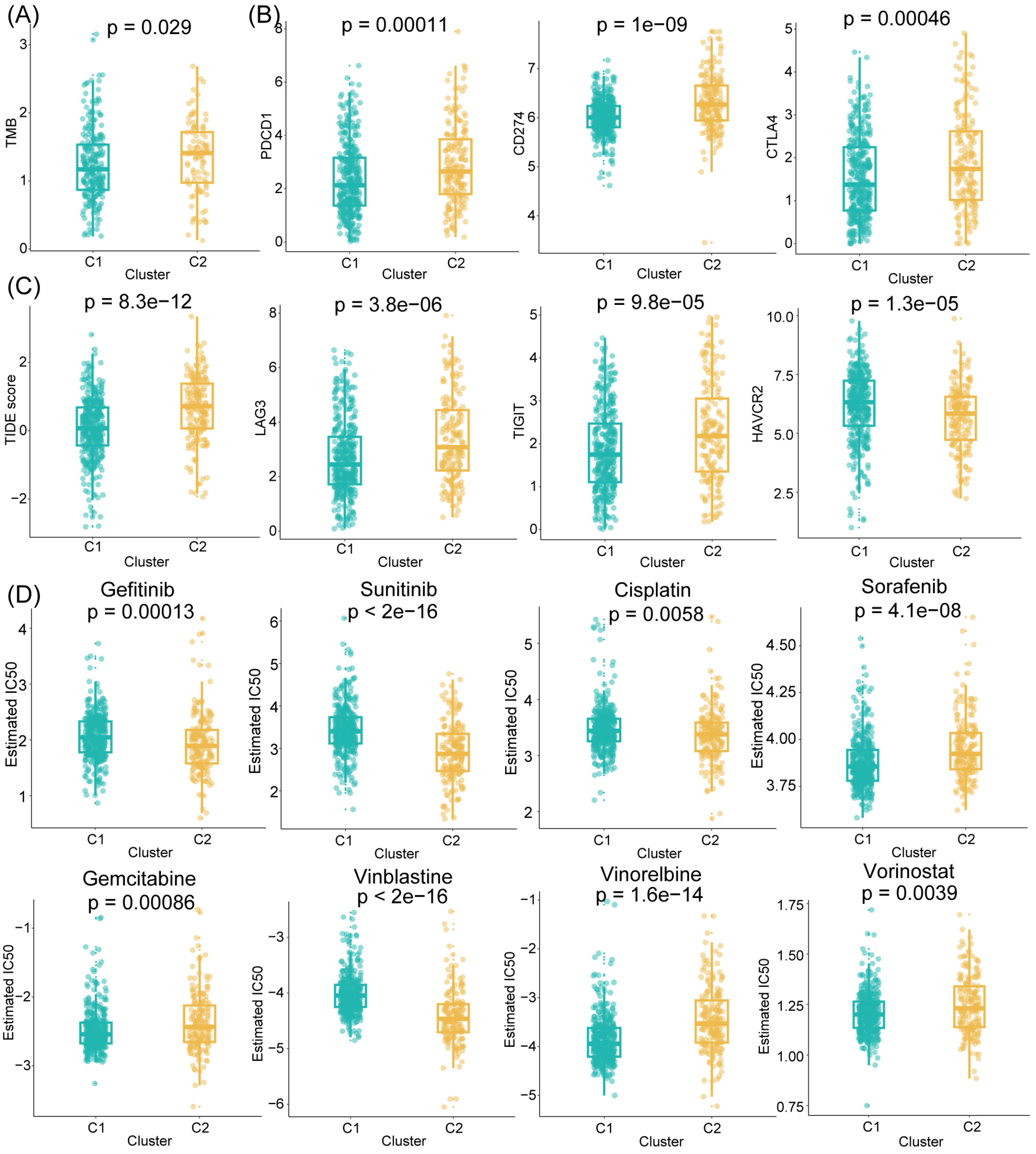

Figure S5 The assessment of two TTC clusters in immune characteristics and chemotherapy.

(A,C) The TMB score and TIDE score between two TTC clusters.

(B) The expression of immune checkpoint molecules in two TTC clusters.

(D) The chemotherapy response of two TTC clusters for eight frequently used drugs.

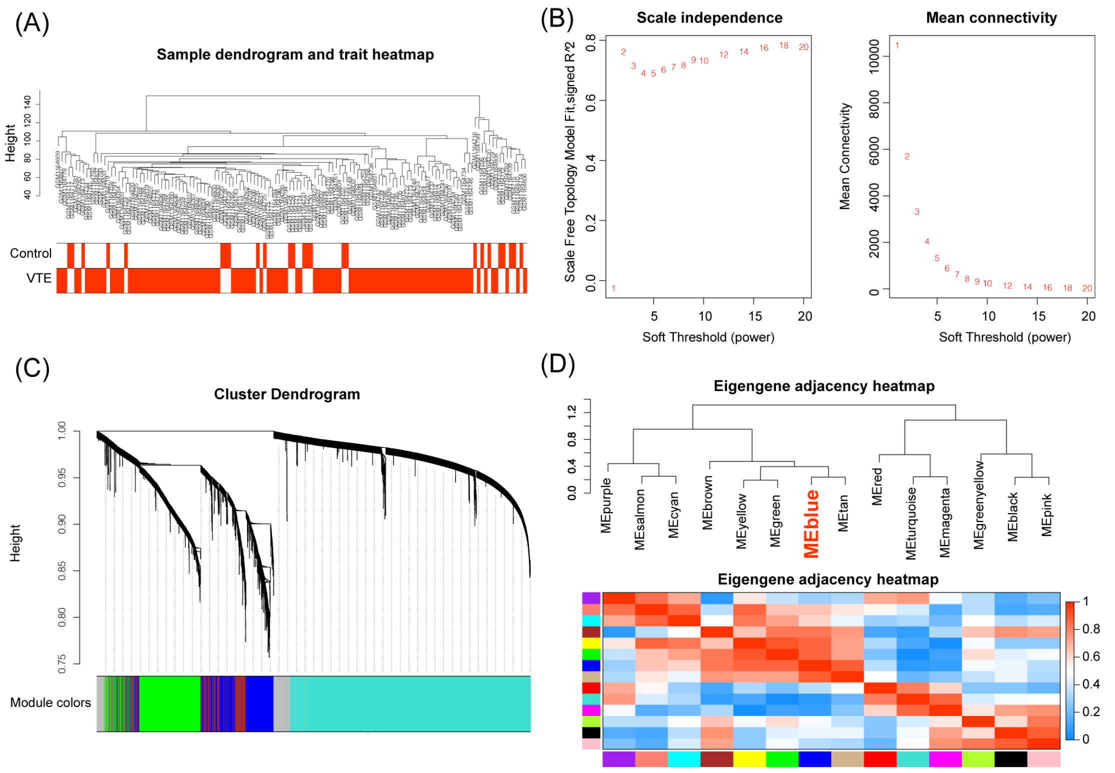

Figure S6 The procedure of WGCNA analysis.

(A) Clustering analysis of samples from the GSE48000 cohort based on the mRNA expression profile to detect outliers. Each branch represents a sample, and the y-axis represents the cluster distance.

(B) Analysis of the scale-free fit index (R2) and the mean connectivity with different soft-thresholding powers in the GSE48000 cohort.

(C) Gene dendrograms obtained by average linkage hierarchical clustering in the GSE48000 cohort. The modules of expressed genes were assigned colors and numbers as indicated by the horizontal bar beneath each dendrogram (dynamic tree cut).

(D) Hierarchical cluster dendrograms and heatmaps of the correlation between ME values and VTE.

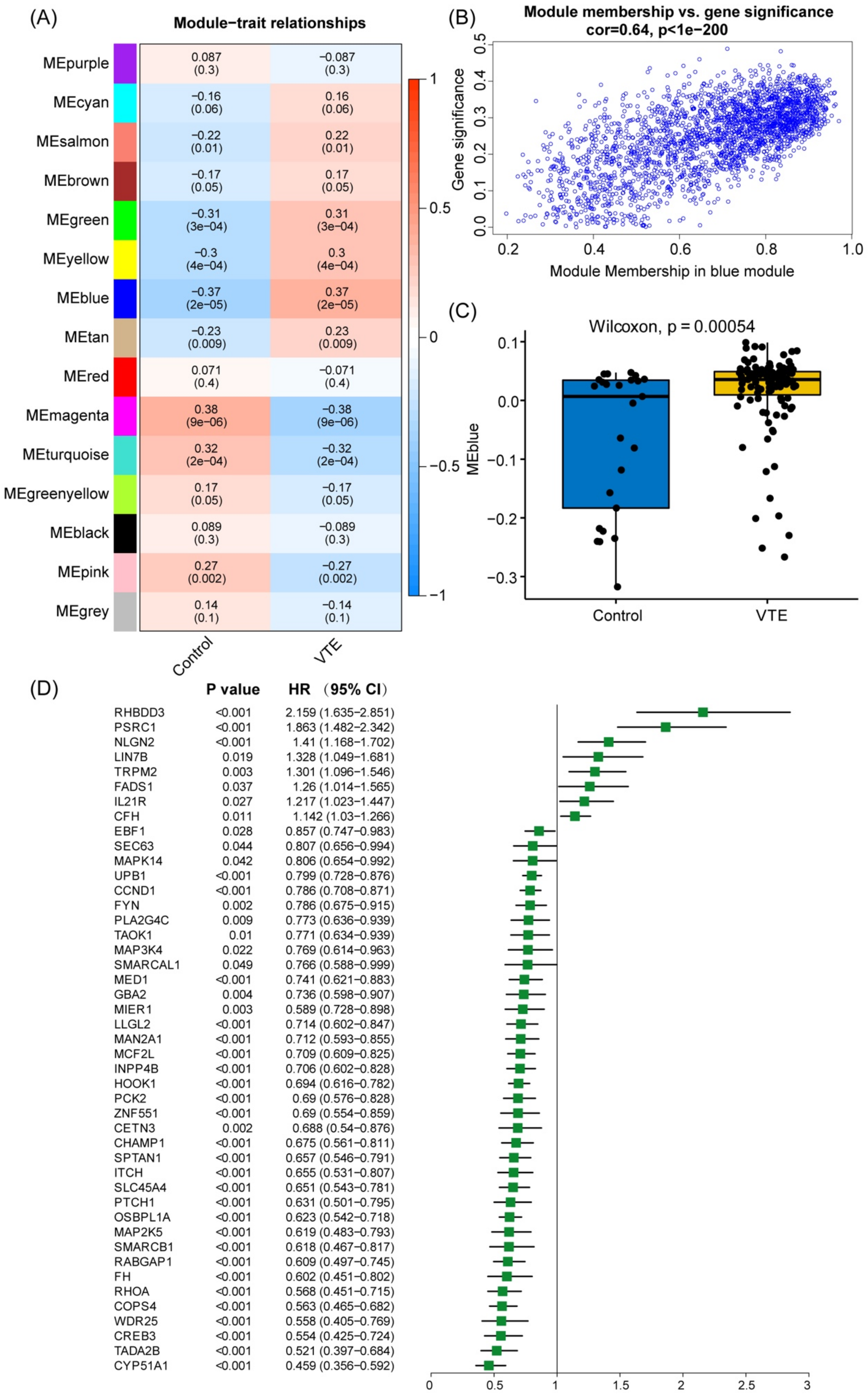

Figure S7 The process of TTCRGs.

(A) Correlation analysis between module eigengenes and clinical traits. The cells were color-coded by the correlation between the module and clinical information according to the color legend on the right with red representing a strong positive correlation and green representing a strong negative correlation.

(B) Scatterplot reveals the high correlation between GS and MM in the blue module with Pearson's correlation coefficient. The dot indicated all genes within the blue module and was defined as TTCRGs in RCC patients.

(C) Boxplot illustrates the relationship of module trait between the control and VTE group in the blue module based on the TCGA cohort. Each dot represents a gene within the blue module.

(D) Univariate Cox analysis identified 45 TTCRGs in the TCGA cohort. Data are visualized as hazard ratio (HR) ± 95% confidence interval (CI).

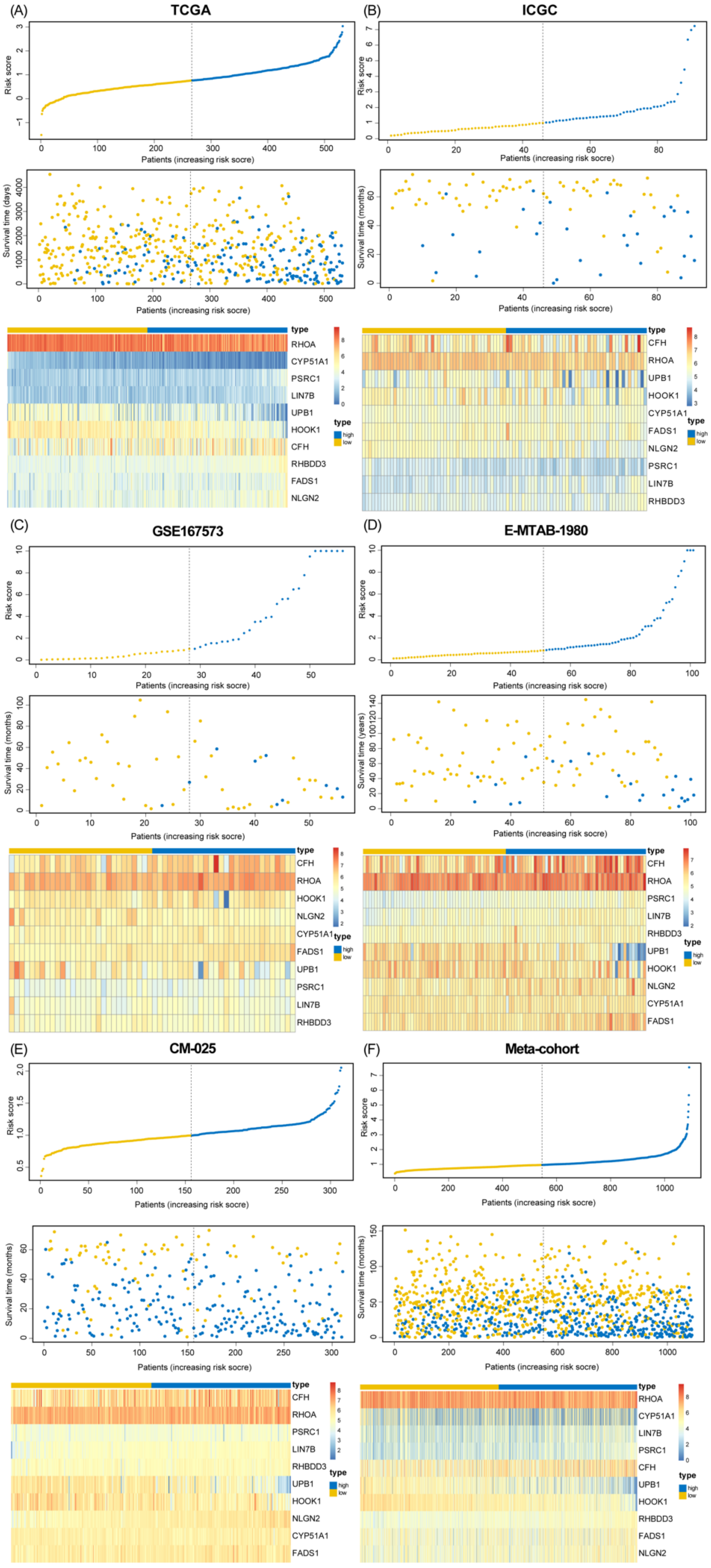

(A)
TCGA
Risk score
Patients (increasing risk socre)
Survival time (days)
type
RHOA
CYP51A1
PSRC1
LIN7B
UPB1
HOOK1
CFH
RHBDD3
FADS1
NLGN2
high
low
(B)
ICGC
Survival time (months)
CFH
RHOA
UPB1
HOOK1
CYP51A1
FADS1
NLGN2
PSRC1
LIN7B
RHBDD3
(C)
GSE167573
CFH
RHOA
HOOK1
NLGN2
CYP51A1
FADS1
UPB1
PSRC1
LIN7B
RHBDD3
(D)
E-MTAB-1980
Survival time (years)
CFH
RHOA
PSRC1
LIN7B
RHBDD3
UPB1
HOOK1
NLGN2
CYP51A1
FADS1
(E)
CM-025
CFH
RHOA
PSRC1
LIN7B
RHBDD3
UPB1
HOOK1
NLGN2
CYP51A1
FADS1
(F)
Meta-cohort
RHOA
CYP51A1
LIN7B
PSRC1
CFH
UPB1
HOOK1
RHBDD3
FADS1
NLGN2

Figure S8 Establishment of the TTCRRS signatures in multiple cohorts.

(A-F) The distribution of TTCRRS signature, the vital status of patients, and the expression of TTCRGs in the TCGA-KIRC, ICGC, GSE167573, E-MTAB-1980, CM-025 and Meta cohorts.

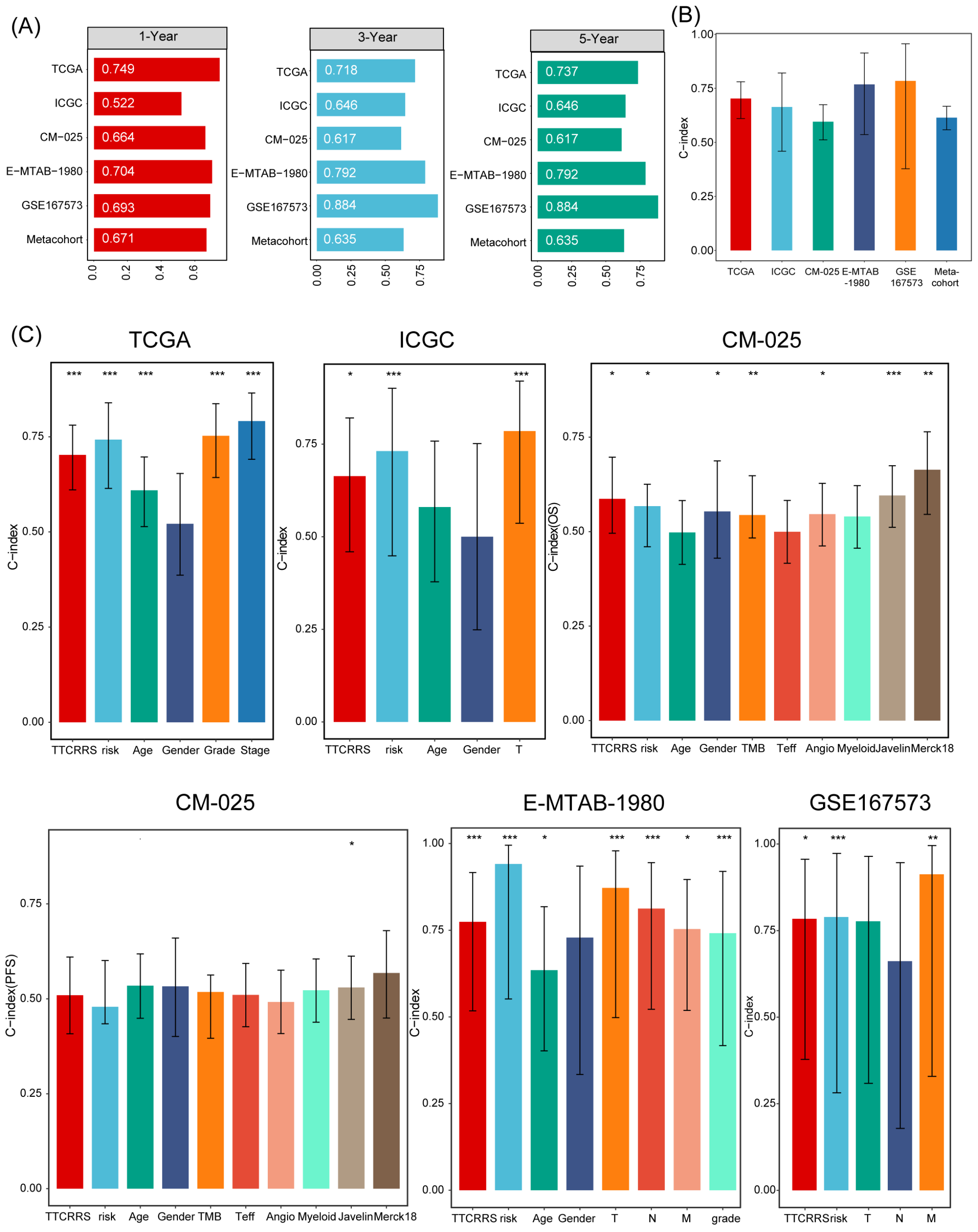

Figure S9 Evaluation of the TTCRRS signature.

(A) Time-dependent ROC analysis for predicting OS at 1, 3, and 5 years across all cohorts.

(B) C-index of TTCRRS across all cohorts.

(C) The performance of TTCRRS was compared with other clinical and molecular variables in predicting survival prognosis. Data are presented as mean ± 95% confidence interval [CI]. *P < 0.05; **P < 0.01; ***P < 0.001.

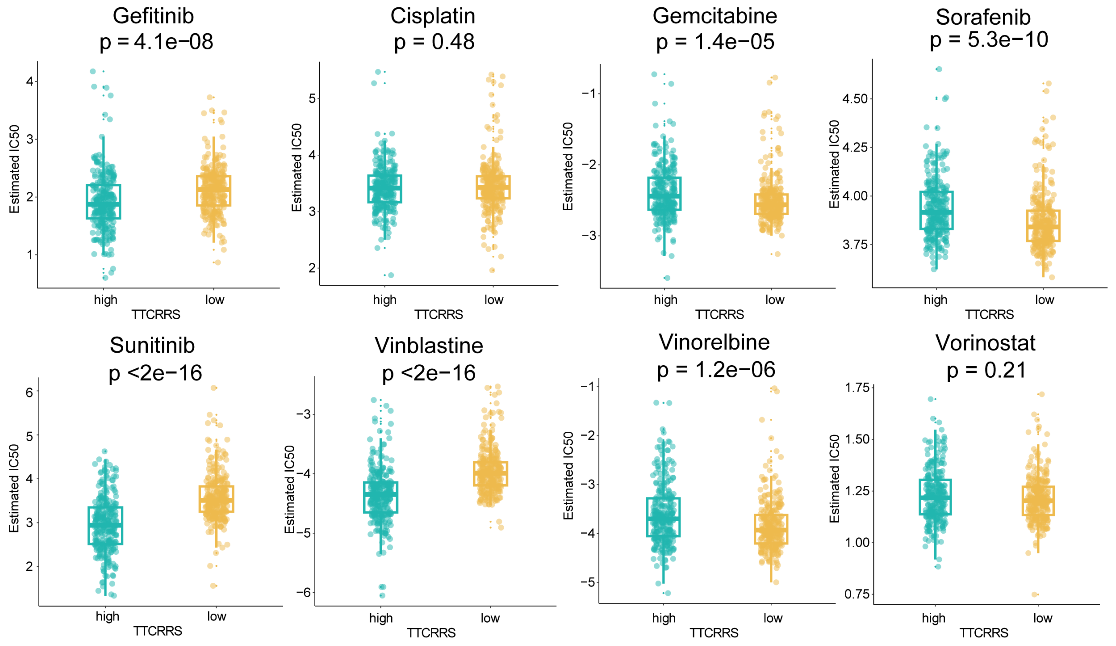

Figure S10 The assessment of two TTCRRS subtypes in chemotherapy for eight common drugs.

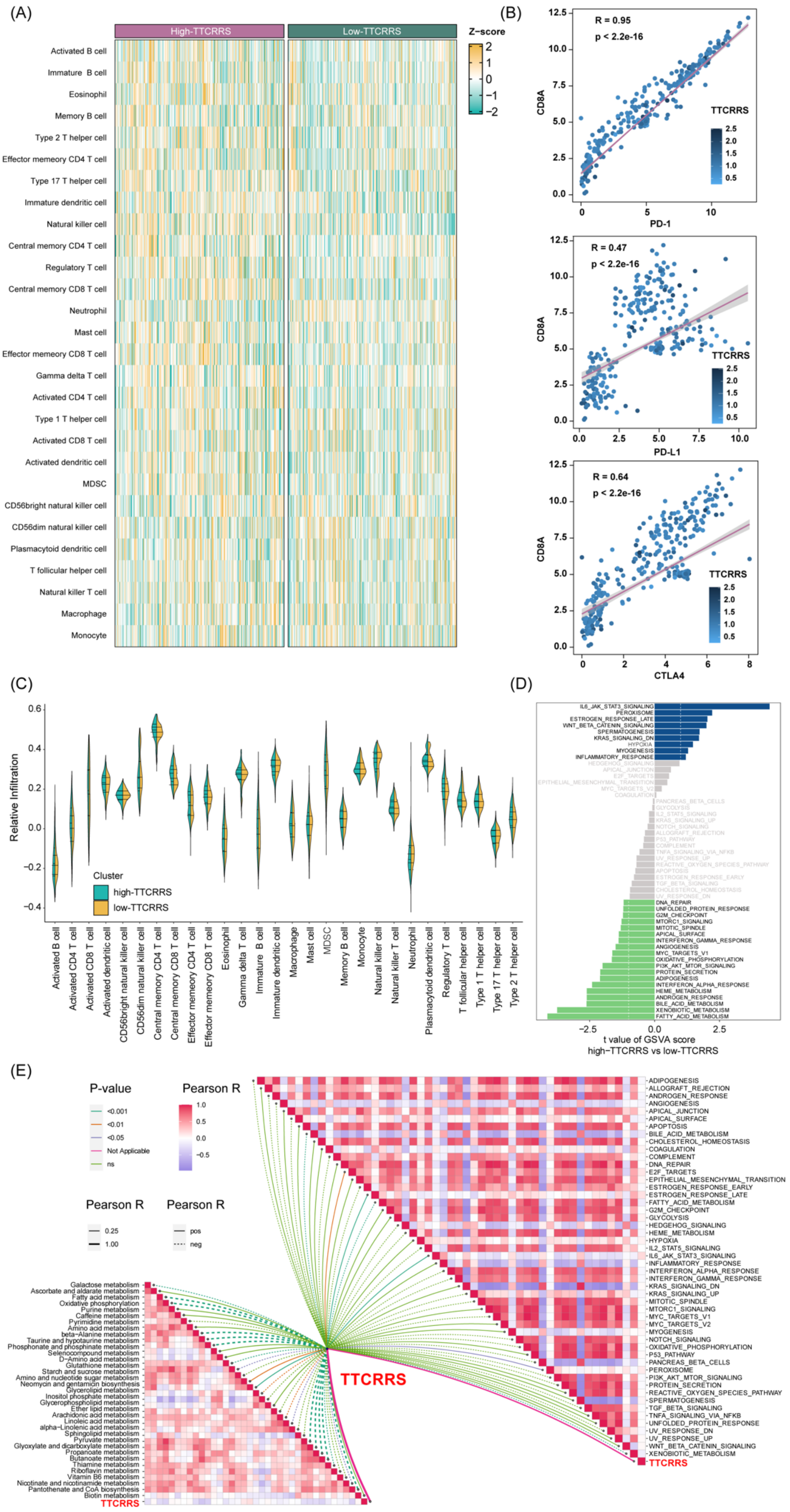
(A)
High-TTCRRS
Low-TTCRRS
Z-score
Activated B cell
Immature B cell
Eosinophil
Memory B cell
Type 2 T helper cell
Effector memeory CD4 T cell
Type 17 T helper cell
Immature dendritic cell
Natural killer cell
Central memory CD4 T cell
Regulatory T cell
Central memory CD8 T cell
Neutrophil
Mast cell
Effector memeory CD8 T cell
Gamma delta T cell
Activated CD4 T cell
Type 1 T helper cell
Activated CD8 T cell
Activated dendritic cell
MDSC
CD56bright natural killer cell
CD56dim natural killer cell
Plasmacytoid dendritic cell
T follicular helper cell
Natural killer T cell
Macrophage
Monocyte
(B)
R = 0.95
p < 2.2e-16
CD8A
PD-1
TTCRRS
R = 0.47
p < 2.2e-16
PD-L1
R = 0.64
p < 2.2e-16
CTLA4
(C)
Relative Infiltration
Cluster
high-TTCRRS
low-TTCRRS
(D)
t value of GSVA score
high−TTCRRS vs low−TTCRRS
(E)
P-value
<0.001
<0.01
<0.05
Not Applicable
ns
Pearson R
pos
neg
TTCRRS

Figure S11 Function annotation of the TTCRRS signature based on the Meta-cohort.

(A) The relationship between two TTCRRS subtypes and 28 immune cell infiltrations.

(B) Scatterplots between CD8A and PD0-1, PD-L1, and CTLA4 with the TTCRRS were shown in the Meta-cohort.

(C) Violin plot shows the relationship between 28 immune cell infiltrations and the TTCRRS subtype.

(D) Difference in pathway activities scored per patient by GSVA between high- and low-TTCRRS. Shown are t values from a linear model.

(E) Butterfly plot illustrates the correlation between the TTCRRS and metabolic pathways, the enrichment pathways based on GSVA of GO and KEGG terms.

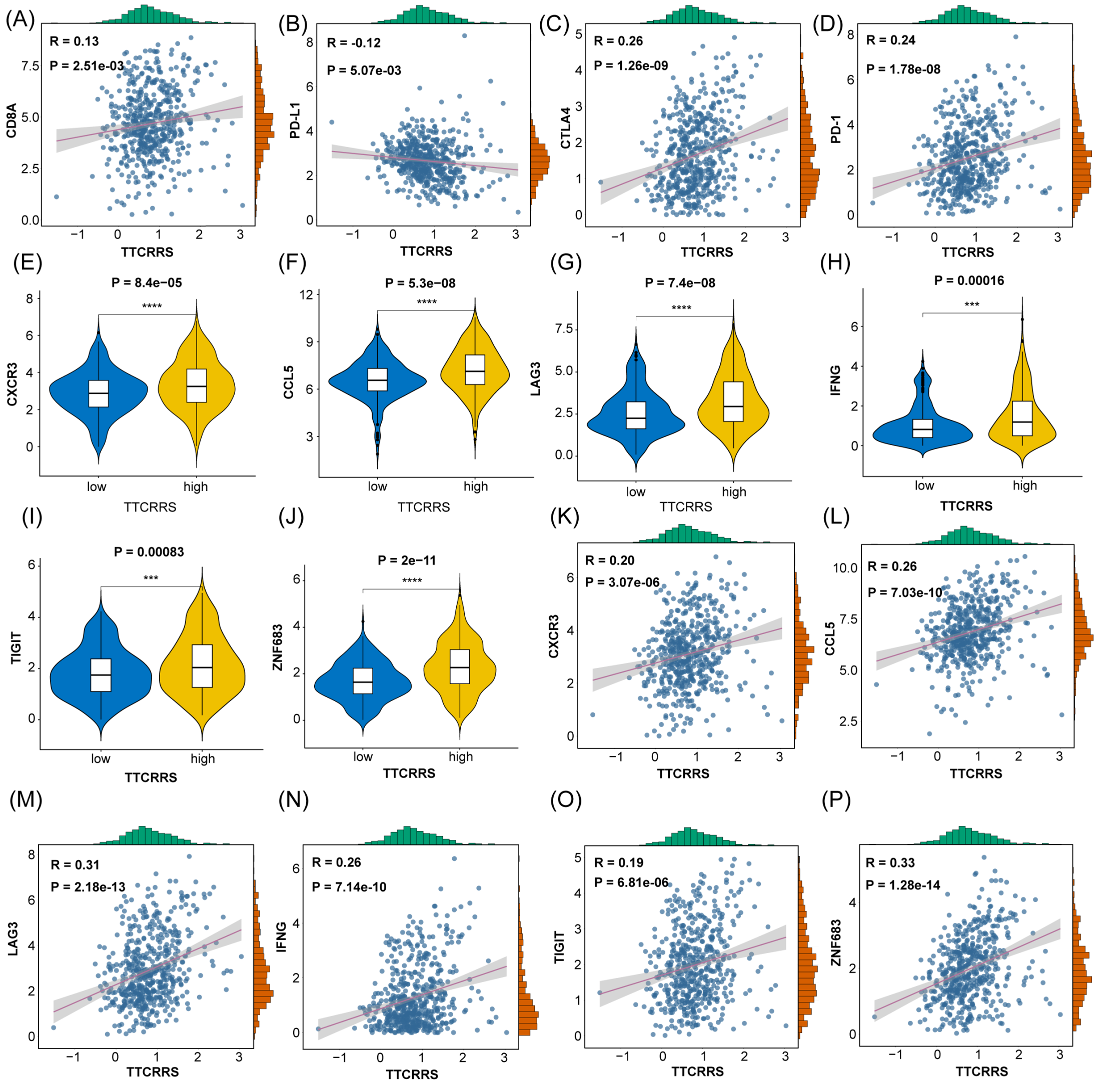

Figure S12 The correlation of TTCRRS with antitumor immunity.

(A-D) Scatterplots show the correlation between TTCRRS and CD8A, PD-L1, CTLA4 and PD-1.

(E-J) The violin plots depict the association between some immune response signatures(CXCR3, CCl5,

LAG3, IFNG, TIGIT, ZNF683) and TTCRRS.

(K-P) Scatterplots show the correlation between TTCRRS and immune response signatures (CXCR3, CCl5, LAG3, IFNG, TIGIT, ZNF683).

Statistic test: Pearson's correlation coefficient, two-sided unpaired t-test. Data are presented as mean ± 95% confidence interval [CI].

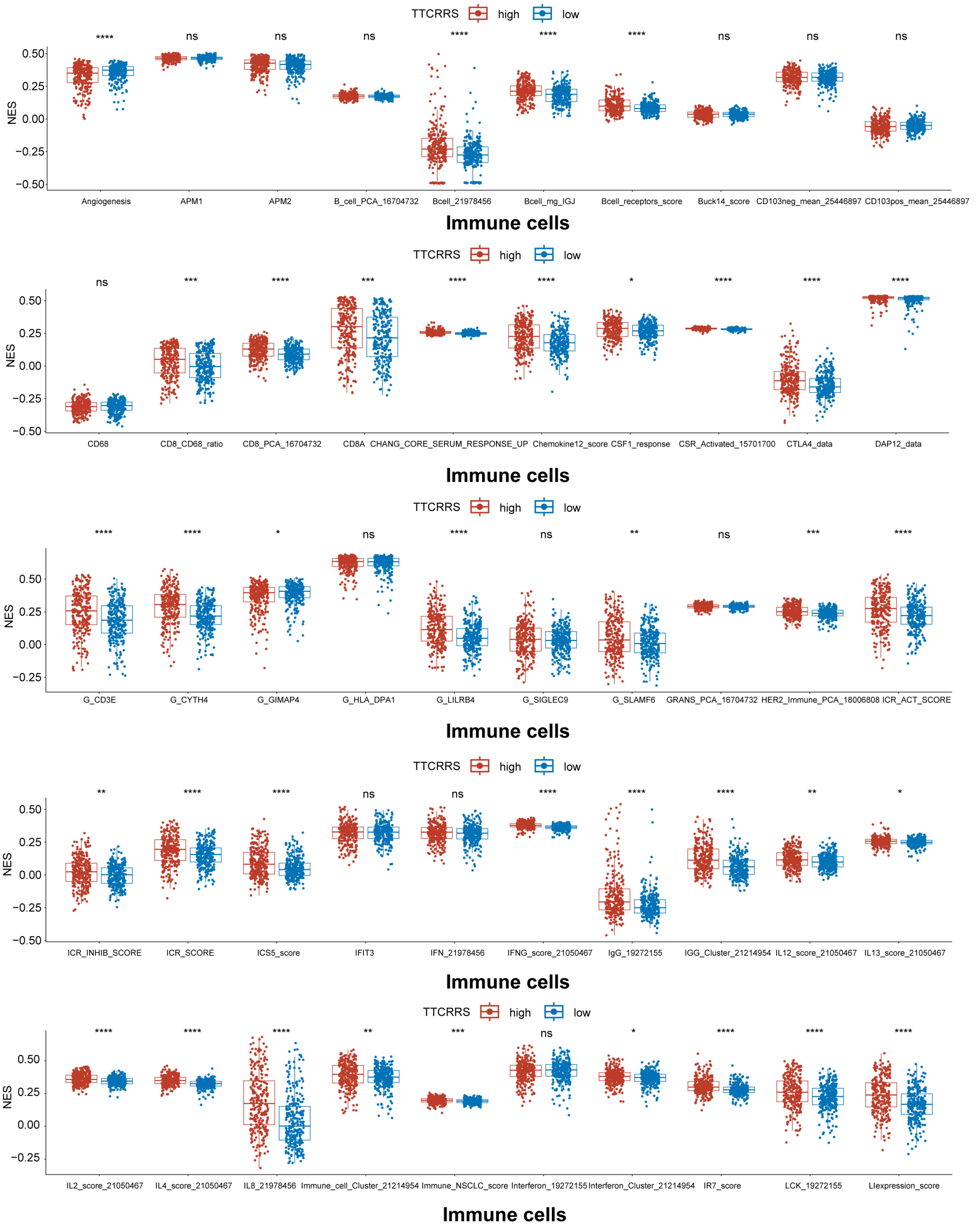

Figure 13 The 181 immune cell infiltrations between two TTCRRS subtypes.

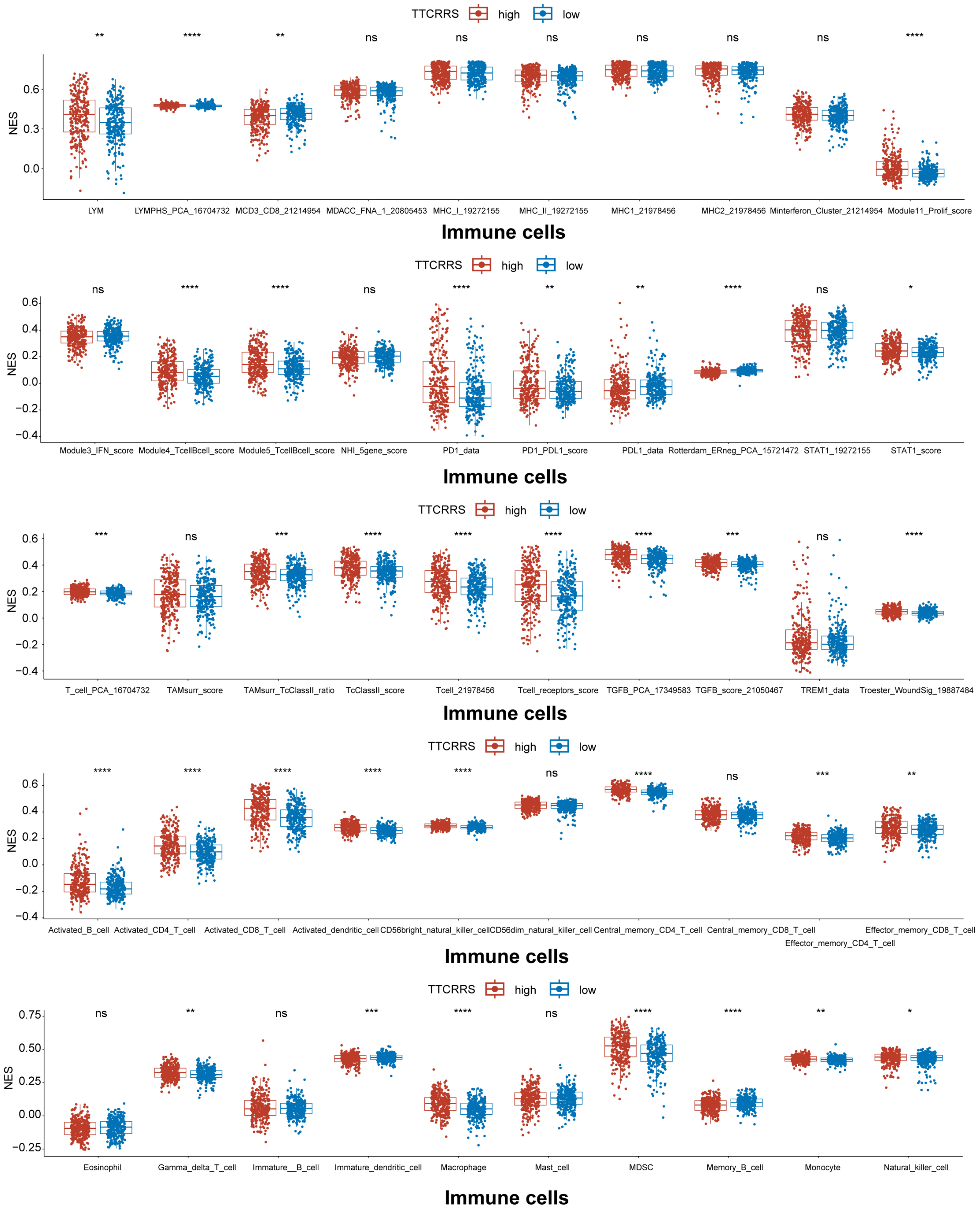

Figure S14 The 181 immune cell infiltrations between two TTCRRS subtypes.

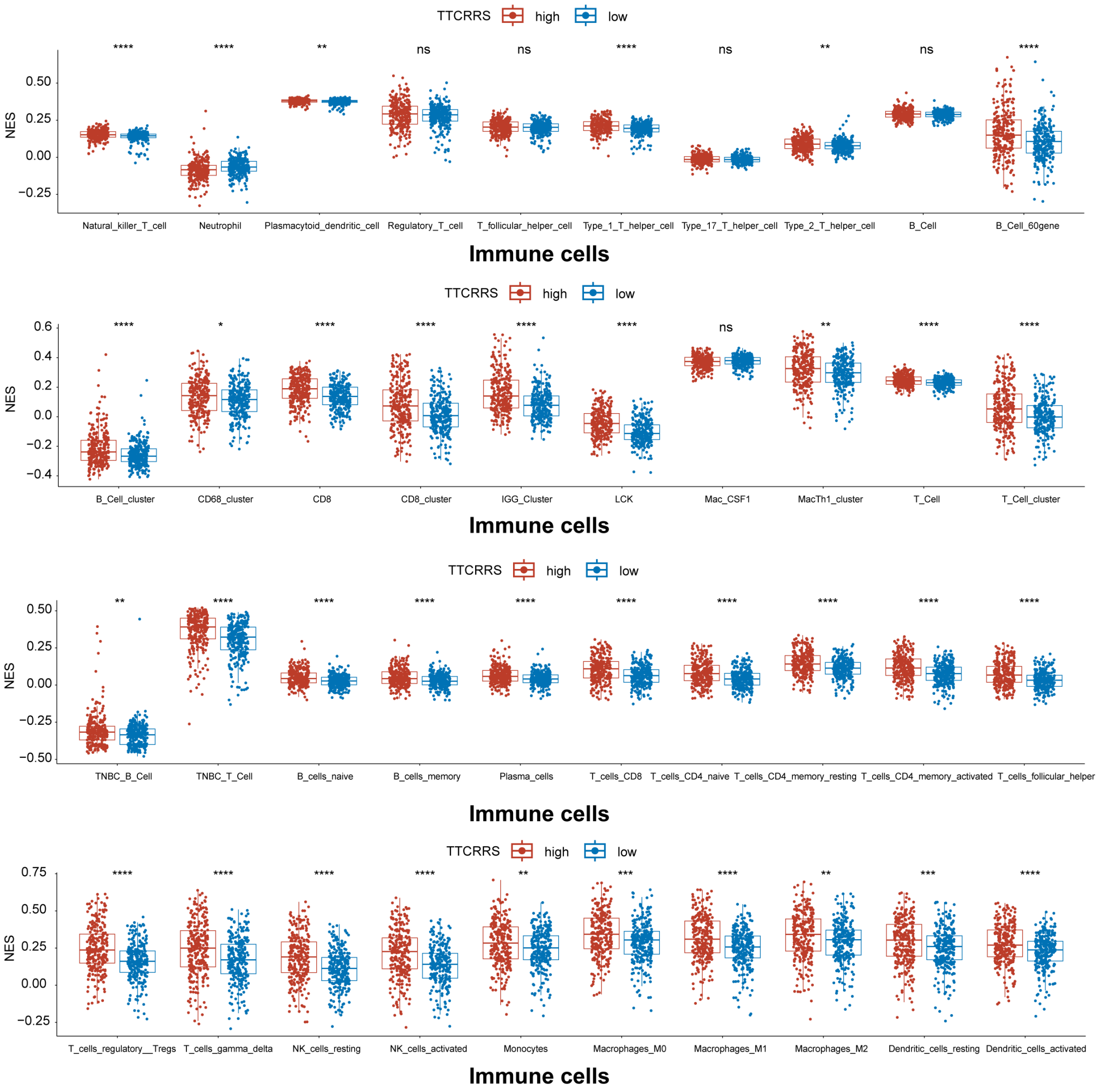

Figure S15 The 181 immune cell infiltrations between two TTCRRS subtypes.

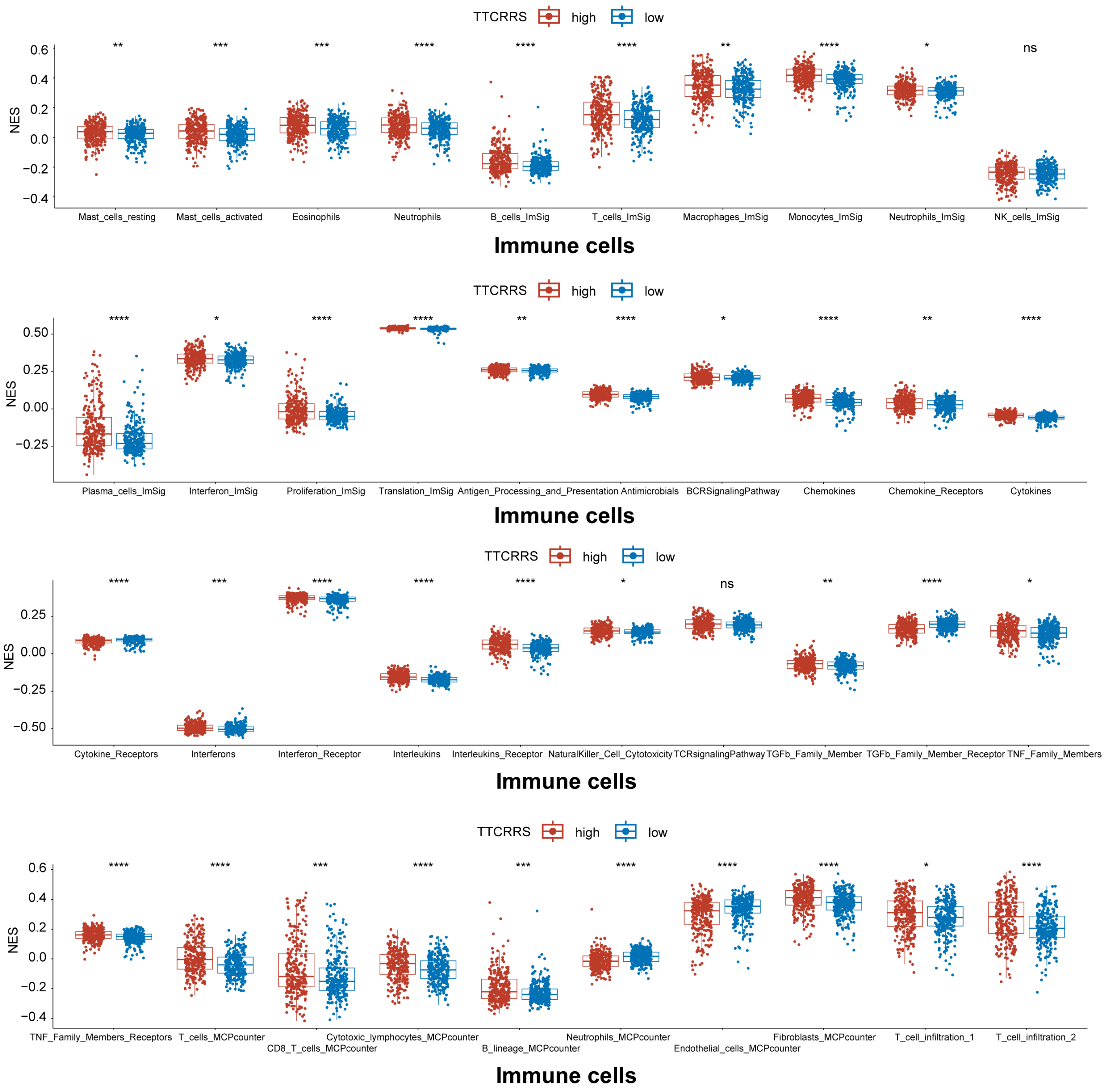

Figure S16 The 181 immune cell infiltrations between two TTCRRS subtypes.

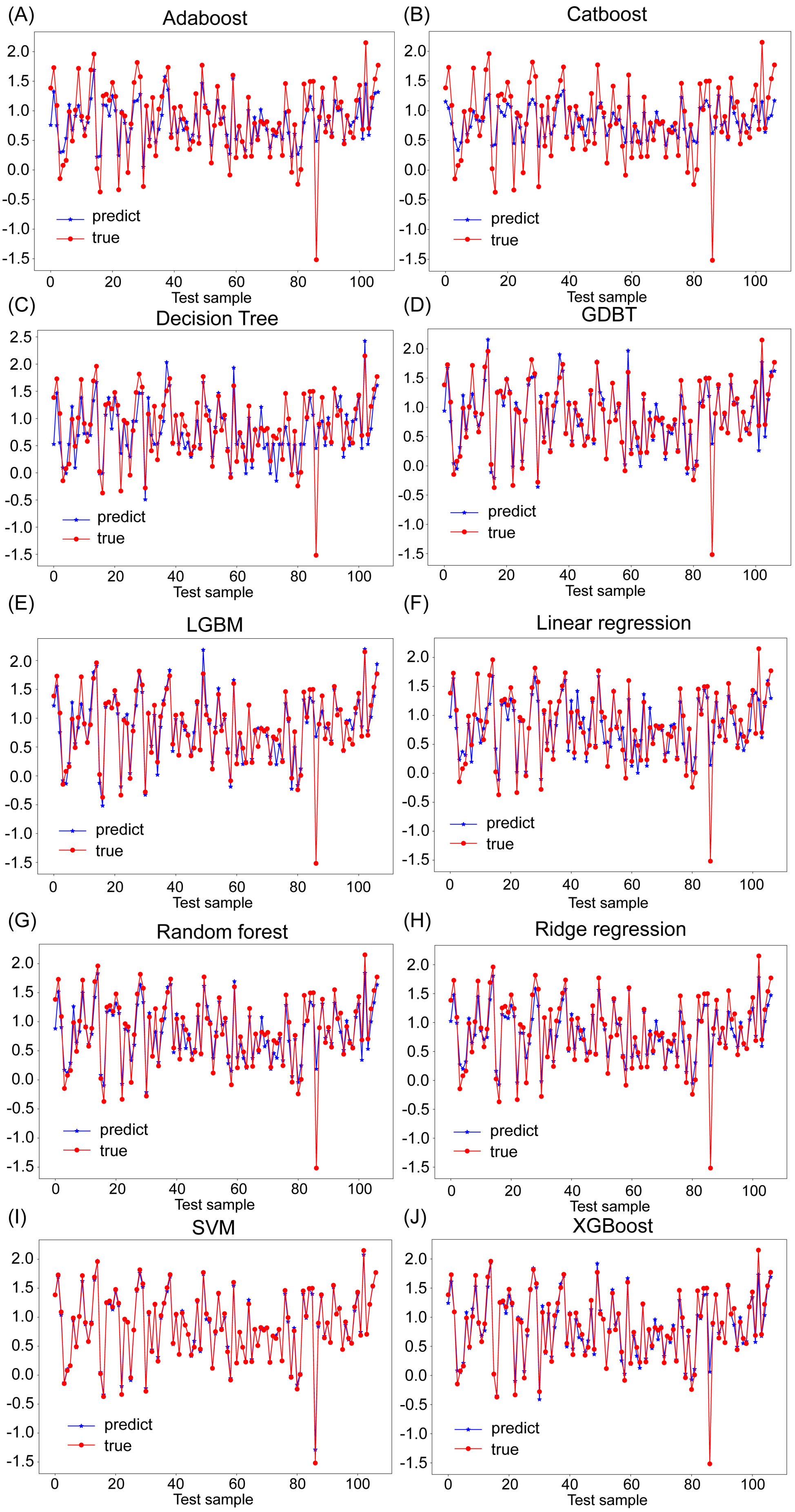
(A)
Adaboost
(B)
Catboost
(C)
Decision Tree
(D)
GDBT
(E)
LGBM
(F)
Linear regression
(G)
Random forest
(H)
Ridge regression
(I)
SVM
(J)
XGBoost
predict
true
Test sample

Figure S17 Accuracy of 10 machine learning classifiers.

(A-J) 10 machine learning classifier accuracy (Adaboost, Catboost, Decision Tree, GDBT, LGBM, Linear regression, random forest, Ridge regression, SVM and XGBoost). Red lines represent true data and blue lines represent predicted data.

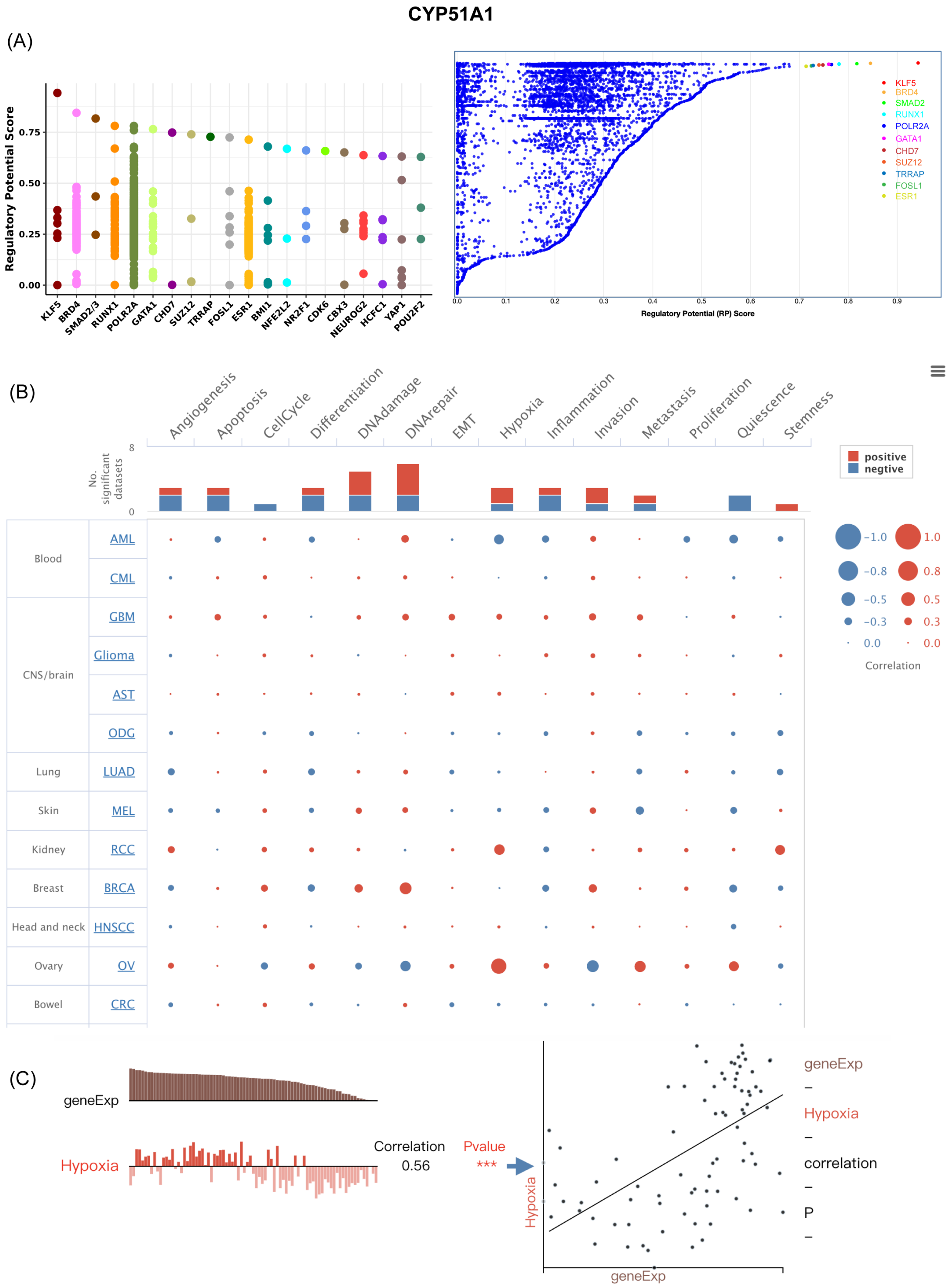

Figure S18 Identification of hub gene-related Transcription factors (TFs).

(A, D) CYP51A1 and PSRC1 were regulated by those most likely TFs in human cancers.

(B) The functional state of CYP51A1 in ccRCC based on CancerSEA. The red plots indicated that CYP51A1 is positively correlated with the functional state while the blue plots indicated that CYP51A1 was negatively correlated with the functional state identified by CancerSEA.

(C) Single-cell analysis indicated that CYP51A1 is primarily involved in Hypoxia in ccRCC and the correlation between CYP51A1 and Hypoxia is shown in the scatter plot.

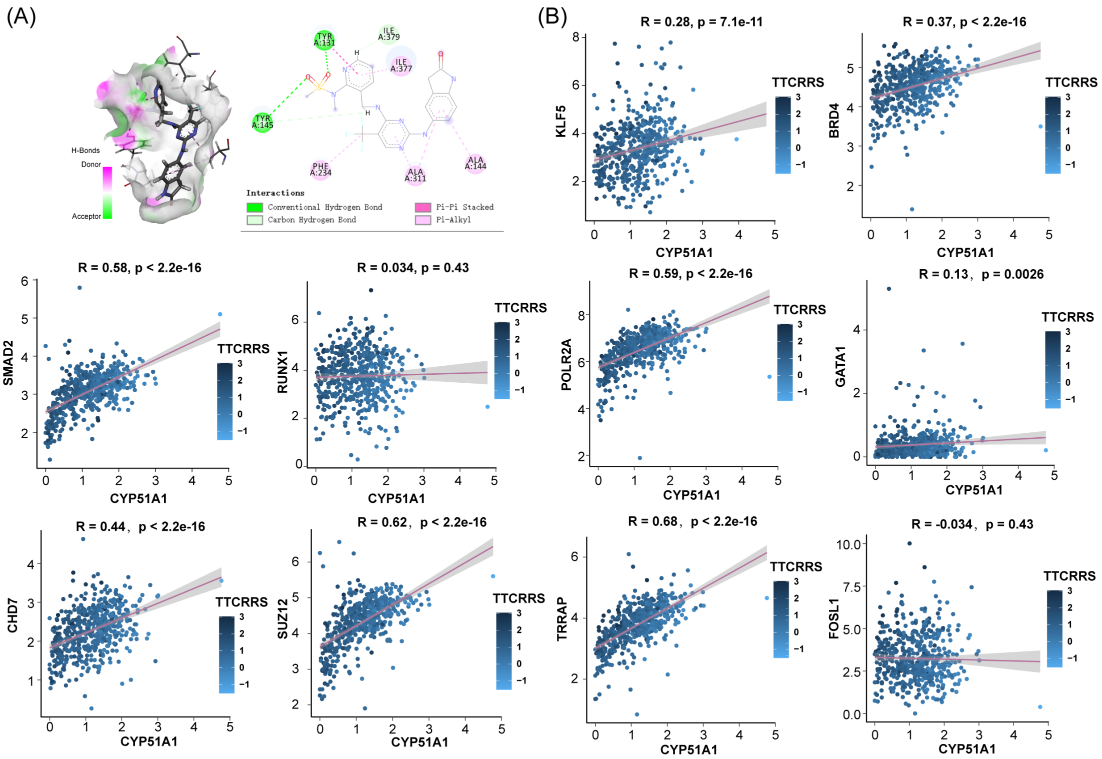

Figure S19 The correlation between hub genes and TFs.

(A) Three-dimensional structure diagram of CYP51A1-PF-562271.

(B) The correlation between CYP51A1 and TFs (KLF5, BRD4, SMAD2, RUNX1, POLR2A, GATA1, CHD7, SUZ12, TRRAP and FOSL1).

Statistic test: Pearson's correlation coefficient, two-sided unpaired t-test. Data are presented as mean ± 95% confidence interval [CI].

Supplementary Tables:

Because some tables are too big, so we provide individually in excel files.
